# Sex-dependent neurobiological and metabolic dysfunction in an aged rat model of post-traumatic stress disorder

**DOI:** 10.1038/s42003-026-10536-x

**Published:** 2026-06-19

**Authors:** Yubo Hu, Wentao Hu, Yueru Zhong, Yu Liu, Peng Xiao

**Affiliations:** Department of Biomedical Engineering, Huazhong University of Science and Technology, Wuhan, PR China

**Keywords:** Neuroscience, Post-traumatic stress disorder

## Abstract

The neurobiological underpinnings of post-traumatic stress disorder (PTSD) in the elderly remain poorly characterized, particularly regarding sex-dependent vulnerabilities. This study systematically investigated sex-specific behavioral and metabolic alterations in 15-month-old rats using a single prolonged stress and footshock (SPS + FS) paradigm. A multimodal approach integrating behavioral analysis, 2-deoxy-2-[¹⁸F]fluoro-D-glucose positron emission tomography (FDG-PET) imaging, plasma endocrine profiling, and brain transcriptomics was employed to assess PTSD-related dysfunction. Behavioral assessments revealed that female rats exhibited more severe PTSD-like manifestations, indicating a higher susceptibility compared to males. Macroscopically, FDG-PET imaging identified prominent sex-specific alterations in glucose metabolism within the core fear circuitry, including the prefrontal cortex (PFC), hippocampus (Hipp), and amygdala (Amyg). In females, these metabolic shifts were more pronounced and strongly correlated with behavioral phenotypes. Dynamic PET further confirmed a general decline in the cerebral metabolic rate of glucose (CMRglu) across PTSD groups. Transcriptomic analysis validated these observations, revealing a systemic reprogramming of central metabolic networks in core brain regions. These neuroenergetic shifts appeared potentially associated with the dysregulation of key metabolic signaling networks, primarily the insulin and AMPK pathways, which exhibited notable sex-dependent spatial heterogeneity. These findings elucidate sex-specific metabolic and molecular abnormalities in aged PTSD models, advancing our understanding of elderly pathophysiology.

## Abstract (web summary)

Multimodal imaging and transcriptomic profiling in a 15-month-old rat model reveal distinct sex-dependent abnormalities in core fear circuitry glucose metabolism, driven by spatial tuning of insulin-AMPK signaling networks.

## Introduction

Post-traumatic stress disorder (PTSD) is a debilitating psychiatric condition that develops following exposure to an extreme traumatic event. Its core clinical manifestations include intrusive memories, avoidance behaviors, negative alterations in cognition and mood, and heightened arousal^1,2^. Globally, PTSD severely impairs patients’ quality of life, and epidemiological evidence consistently shows that the lifetime prevalence of PTSD is substantially higher in women than in men^3^. This sex disparity reflects a complex interplay between biological factors (e.g., gonadal hormones) and psychosocial elements, underscoring the critical need to systematically integrate sex differences into PTSD research^4^. Furthermore, while significant advances have been made, most clinical and preclinical studies have focused on young or middle-aged populations^5,6^. Given the unique physiological baselines of older demographics, including natural endocrine transitions and altered neuroplasticity, the pathological mechanisms underlying PTSD in older populations may present distinct characteristics^7^. Consequently, investigating the sex-specific pathogenesis of PTSD within an older demographic context remains a critical clinical priority.

To elucidate the neurobiological underpinnings of PTSD, research has primarily focused on the core fear circuit, which comprises the prefrontal cortex (PFC), hippocampus (Hipp), and amygdala (Amyg)^8^. Typically, the PFC plays a crucial role in regulating emotions and inhibiting fear responses by modulating amygdala activity^9–11^, while the hippocampus remains central to contextual memory processing^12,13^. In individuals with PTSD, however, this vital circuit is often profoundly disrupted. Positron emission tomography (PET), particularly using the 2-deoxy-2-[¹⁸F] fluoro-D-glucose (FDG) radiotracer, has emerged as a powerful non-invasive tool to quantify glucose metabolism within these core brain regions^14–17^. Given that glucose consumption directly reflects regional neural activity, FDG-PET provides a critical in vivo window into the macroscopic neuroenergetic abnormalities associated with PTSD.

While neuroimaging offers essential macroscopic insights, the pathophysiology of PTSD involves complex and multi-level processes^18^. A critical knowledge gap remains in connecting these region-specific metabolic alterations to their underlying microscopic molecular mechanisms. Consequently, the integration of macroscopic neuroimaging with high-throughput molecular approaches, such as transcriptomics, has emerged as a valuable strategy^19,20^. Rather than focusing on isolated gene markers, a holistic transcriptomic approach can facilitate the systematic mapping of how traumatic stress disrupts central metabolic networks. Specifically, identifying the transcriptional reprogramming of key neuroenergetic cascades, including the central insulin and AMPK signaling networks, helps to illuminate the molecular shifts that occur in response to stress-induced energy deficits^21–26^.

To bridge the gap between macroscopic metabolic phenotypes and microscopic molecular architectures, the present study aimed to evaluate sex-dependent alterations in brain glucose metabolism following traumatic exposure using a 15-month-old rat model. By employing a multimodal approach that integrates FDG-PET imaging, plasma endocrine profiling, and targeted brain transcriptomics, we hypothesize that this integrated methodology will reveal a clear mechanistic link between macroscopic brain metabolic abnormalities and the underlying molecular regulatory pathways. Ultimately, these findings are expected to identify novel molecular targets and therapeutic strategies tailored to address the metabolic pathology and prominent sex differences observed in PTSD.

## Materials and methods

### Animals

A total of 24 fifteen-month-old Sprague–Dawley (SD) rats, including both males and females (Wuhan Luobin Life Science Co., Ltd.), were used as an aged animal model. To ensure environmental consistency and eliminate confounding variables associated with social hierarchy or social buffering, all rats, including the controls, were individually housed in a controlled environment with a temperature of 22 ± 2 °C, humidity of 50 ± 5%, and a 12-h light–dark cycle (lights on at 07:00). All animals had ad libitum access to food and water.

Before the initiation of experimental procedures, all rats underwent a minimum 1-week acclimatization period. The study protocols adhered to the guidelines of the National Animal Ethics Committee and received formal approval from the Ethics Committee of Huazhong University of Science and Technology. Based on sex and experimental objectives, the rats were allocated into four groups (*n* = 6 per group): male control (CK male), male PTSD model (PTSD male), female control (CK female), and female PTSD model (PTSD female). Control animals remained in the same housing room and were handled daily for 5 min to habituate them to the experimenter. During the stress induction period, control rats were transported to the experimental rooms alongside the PTSD groups to account for non-specific stressors such as environmental novelty and transportation, although they did not undergo the SPS + FS protocol.

### Animal modeling and behavioral/neuroimaging timeline

**Fig. 1:**
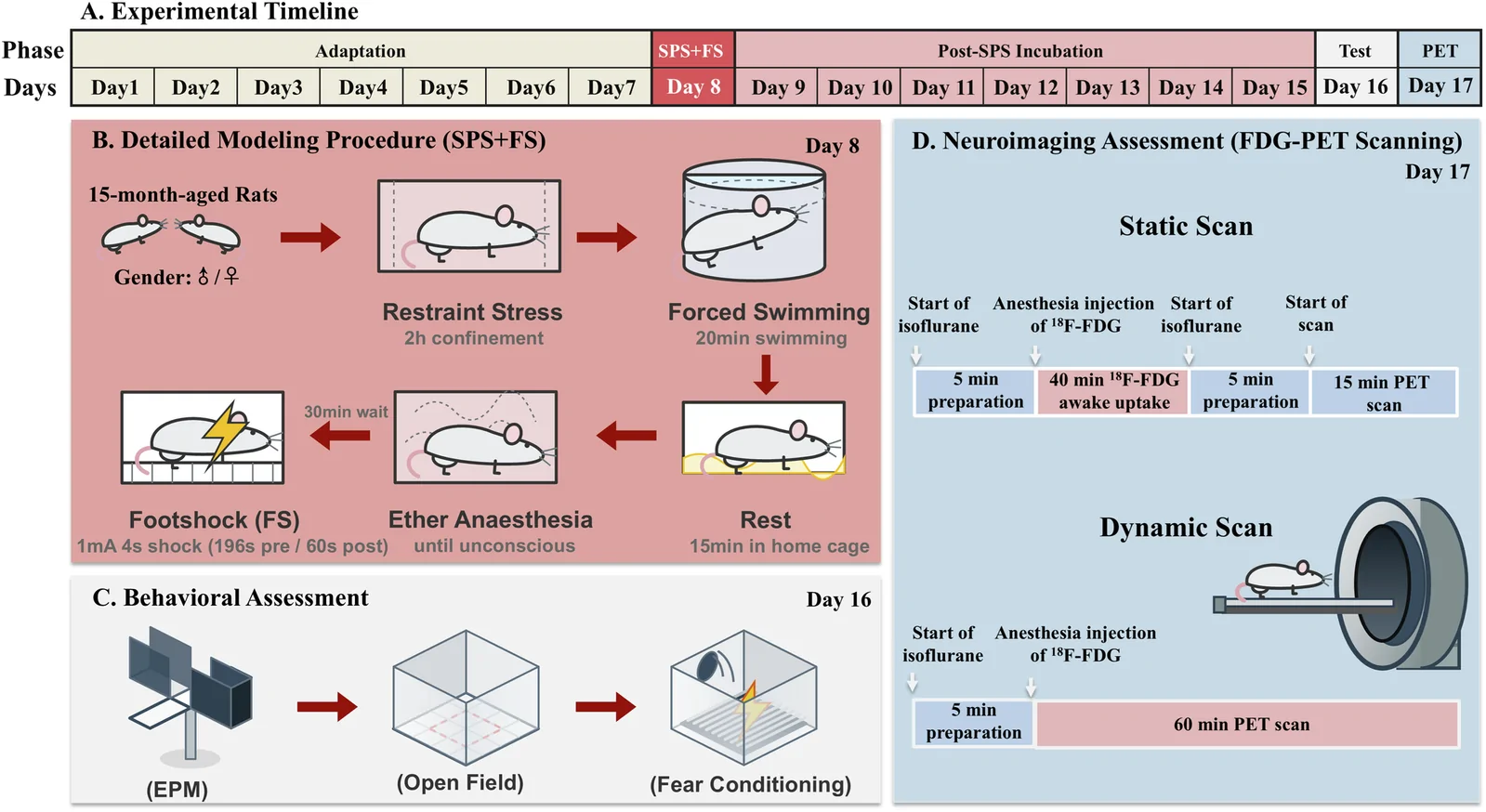
Schematic overview of the experimental design and timeline. **A** The 17-day overall experimental timeline encompasses the adaptation period, the single prolonged stress and footshock (SPS + FS) modeling paradigm, post-trauma incubation, behavioral testing, and PET neuroimaging. **B** Detailed schematic of the SPS + FS induction procedure performed on 15-month-old male and female rats on Day 8. The paradigm sequentially includes 2 h of restraint stress, 20 min of forced swimming, 15 min of rest, and ether anesthesia followed by a single inescapable footshock (1 mA, 4 s) after a 30-min recovery interval. **C** Sequence of behavioral assessments conducted on Day 16, including the elevated plus maze (EPM), open field test (OFT), and fear conditioning evaluation. **D** Protocols for the static and dynamic ¹⁸F-FDG PET neuroimaging assessments conducted on Day 17.

The overall experimental paradigm, encompassing PTSD model induction, behavioral assessments, and in vivo neuroimaging, is systematically illustrated in Fig. 1. Following a 7-day adaptation period, a PTSD rat model was established on Day 8 using a modified Single Prolonged Stress and footshock (SPS + FS) paradigm^27^. Briefly, 15-month-old male and female rats were first immobilized in a restraint cylinder for 2 h. Immediately thereafter, they were placed in a transparent cylindrical tank containing water at 22–24 °C for a 20-min forced swim. Following the swim, the rats were dried and allowed to recover in a resting cage for 15 min, after which they were subjected to ether anesthesia until complete loss of consciousness.

Thirty minutes after recovery from anesthesia, the footshock (FS) procedure was performed to induce contextual fear conditioning. Rats were placed in a conditioning chamber for a 196-s habituation period, followed by a single inescapable footshock (1 mA, 4 s). The rats remained in the chamber for an additional 60 s post-shock before being returned to their home cages. Following a 7-day post-trauma incubation period (Days 9–15), a comprehensive battery of behavioral tests was conducted on Day 16, followed by dynamic and static ¹⁸F-FDG PET neuroimaging assessments on Day 17.

### Behavioral testing

To systematically assess the impact of trauma, a battery of behavioral tests was conducted within one week of model establishment. Contextual fear conditioning was employed to evaluate trauma-associated fear memory, while the open field test (OFT) and elevated plus maze (EPM) were utilized to assess general anxiety-like behavior and locomotor activity. All tests were performed in a dedicated, soundproofed room with uniform lighting. Animal movements and freezing behaviors were automatically recorded and analyzed using the DigiBehv professional video tracking system (Shanghai Jiliang Software Technology Co., Ltd., China).

The OFT evaluated baseline anxiety and locomotor activity^28^. Rats were individually placed in the center of a white, square open-field arena (100 × 100 × 50 cm). Following a 3-min acclimation period, their movements were recorded for 10 min. Key metrics included total distance traveled, distance traveled in the central area, and time spent in the central area.

The EPM was used to assess avoidance behavior in a high-risk environment^29^. The maze consists of two open arms and two closed arms, elevated 50 cm above the floor. Rats were placed at the intersection of the open and closed arms, facing one of the open arms. Rats were placed at the central intersection facing an open arm and recorded for 5 min. Parameters analyzed included the number of open arm entries, time spent in open arms, and closed arm entries. To quantify anxiety levels, the percentage of time spent in open arms (OT%) and the percentage of open arm entries (OE%) were calculated.

The fear box test is used to assess contextual fear memory and freezing behavior in rats^30^. Rats were placed back into the fear acclimation box and allowed to freely explore for 5 min. The total distance traveled and the duration of freezing during this period were recorded and analyzed using a video tracking system. Freezing behavior is defined as a stereotyped behavior characterized by the absence of all muscle movements except for respiratory movements. The percentage of freezing time is considered a core indicator for quantifying contextual fear memory.

### ELISA analysis

Upon completion of all behavioral assessments, all subjects were anesthetized for blood collection during the light phase (between 09:00 and 11:00) to minimize the impact of circadian fluctuations on hormonal levels. For endocrine analysis, blood was collected via the retro-orbital venous plexus. Samples were centrifuged at 1006×*g* for 15 min at 4 °C to separate plasma. Plasma concentrations of testosterone (T), estradiol (E2), corticosterone (CORT), and brain-derived neurotrophic factor (BDNF) were quantified using specific ELISA kits from Shanghai Affandi Industrial Co., Ltd., China (Catalog Nos: T, A109234; E2, A108219; CORT, A107689; BDNF, A107739).

Briefly, 10 µL of plasma sample was added to 40 µL of sample diluent (5-fold dilution) in pre-coated microplates. Following the sequential addition of HRP-conjugated antibodies and TMB substrate, the reaction was terminated. Absorbance was measured at 450 nm using a microplate reader. Concentrations were determined by interpolating the OD values into a standard curve generated using a four-parameter logistic (4PL) regression model. The concentrations of T, E2, CORT, and BDNF were expressed in nmol/L, ng/L, µg/mL, and ng/mL, respectively, in accordance with the manufacturer’s specifications.

### PET imaging and data analysis

Prior to the PET scan, all rats were fasted for 12 h. 2-Deoxy-2-[¹⁸F]fluoro-D-glucose (FDG) was administered via the tail vein at a dose of 50 MBq/kg. After injection, the rats were kept in a quiet environment for a 40 min tracer uptake period to allow FDG accumulation in the brain. Subsequently, the animals were anesthetized with 3% isoflurane and positioned in a Discoverist 180 small-animal PET/CT scanner for imaging.

A 15-min static PET scan was conducted immediately after the tracer uptake period. Static images were reconstructed using the 3D ordered subset expectation maximization (OSEM) algorithm and subsequently spatially normalized with the statistical parametric mapping (SPM) toolbox^31^. For accurate quantification of regional glucose metabolism, voxel-wise analysis was performed using the SPM-based Anatomical Toolbox (SAMIT) within SPM. This toolbox automatically aligns images to a standard template and extracts ROI values for core brain regions (prefrontal cortex (PFC), hippocampus (Hipp), amygdala (Amyg)) based on the template. Finally, each target brain region’s standardized uptake value (SUV) was calculated from the extracted ROI values.

Immediately after the static PET scan, blood glucose levels were measured via tail vein sampling (Table S1). This was followed by a 60-min dynamic PET scan to acquire time–activity data reflecting tracer uptake kinetics^32^. Dynamic images were reconstructed using the 3D OSEM algorithm (2 iterations, 12 subsets) with attenuation correction applied. The resulting dynamic time–activity curves were processed using software, including Amide and MATLAB. The cerebral metabolic rate of glucose (CMRglu) was then calculated from the dynamic data using well-established kinetic models, particularly the Patlak analysis.

### Tissue processing and RNA extraction

Immediately after the completion of PET imaging, rats were euthanized, and their brains were rapidly removed and placed on an ice-cold surface. Following the rat brain atlas of Paxinos and Watson, the prefrontal cortex (PFC), hippocampus (Hipp), and amygdala (Amyg) were precisely dissected on ice. The isolated tissues were immediately snap-frozen in liquid nitrogen and stored at −80 °C until further processing.

For RNA extraction, approximately 30–50 mg of frozen brain tissue from each specific region was homogenized in 1 mL of TRNzol Universal Reagent (TIANGEN, Beijing, China, Cat. DP424) using a high-throughput tissue homogenizer (60 Hz, 120 s). All processing steps were performed on ice or in a 4 °C environment. To ensure complete dissociation of nucleoprotein complexes, the homogenates were incubated at room temperature for 5 min. Subsequently, 0.2 mL of chloroform was added, and the mixture was shaken for 15 s, followed by a 3-min incubation. The samples were centrifuged at 13,400×*g* for 15 min at 4 °C. The upper aqueous phase was collected and mixed with an equal volume of isopropanol, then incubated for 10 min. After centrifugation at 13,400×*g* for 10 min, the RNA pellet was washed with 1 mL of 75% ethanol and centrifuged at 9391×*g* for 5 min. Total RNA integrity was verified (RIN > 8.0, 28S/18S > 1.8) using an Agilent 2100 Bioanalyzer (Agilent Technologies, USA) before library construction.

### Transcriptomics analysis

#### Library preparation and sequencing

Messenger RNA (mRNA) was enriched from qualified total RNA using the poly(A) mRNA Capture Module (ABclonal, China, Cat. RK20340). Strand-specific sequencing libraries were constructed using the Fast RNA-seq Lib Prep Kit V2 (ABclonal, Cat. RK20306). This protocol consolidated second-strand cDNA synthesis, end repair, and dA-tailing into a single reaction to ensure high efficiency. High-throughput sequencing was performed on the Illumina NovaSeq 6000 platform (Illumina, San Diego, CA, USA) in a 150-bp paired-end sequencing mode.

#### Bioinformatics and differential expression analysis

Raw sequencing data (fastp files) underwent quality control using FastQC to remove low-quality reads and adapter sequences. The remaining clean reads were aligned to the Rattus norvegicus reference genome (RGSC 6.0/rn6) using the HISAT2 alignment tool. Gene expression levels were quantified as counts using Feature-Counts. Differential expression analysis was performed using the DESeq2 package in R. To control the false discovery rate (FDR) associated with multiple hypothesis testing, raw *P* values were adjusted using the Benjamini-Hochberg (BH) procedure. Differentially expressed genes (DEGs) were identified based on the following stringent criteria: an adjusted *P* value (*P*adj) < 0.05 and an absolute log₂(fold change) >1 (Volcano plots of the identified DEGs are provided in Supplementary Fig. 1).

To specifically investigate the molecular basis of the macroscopic metabolic deficits observed in PET imaging, a targeted background gene set encompassing “glucose metabolism” was constructed. Rat-specific gene lists associated with glycolysis, gluconeogenesis, the TCA cycle, and key metabolic signaling cascades (insulin, glucagon, and AMPK signaling) were retrieved from the QuickGO and KEGG databases. The identified DEGs were strictly intersected with this targeted metabolic gene set to filter for functionally relevant targets. Functional enrichment analysis was then performed on these refined DEGs^33,34^.

Notably, to pinpoint the regulatory core of the highly enriched insulin signaling pathway, its mapped genes were extracted to construct a protein–protein interaction (PPI) network via the STRING database. To delineate distinct functional modules across different groups, network clustering was executed directly within the STRING platform, utilizing both MCL (Markov Cluster Algorithm) and K-means clustering algorithms. Finally, the core hub genes orchestrating the insulin signaling network were isolated from these functional clusters and subjected to gene expression clustering analysis to visualize their precise regulatory patterns^35–39^.

### Statistics and reproducibility

Data are presented as mean ± SEM. To ensure maximum data transparency, individual biological replicate data points are fully displayed overlying the summary statistics in all corresponding continuous plots. All statistical analyses were performed using GraphPad Prism (v9.0) and R (v4.2.1). The sample size was defined as *n* = 6 independent biological replicates derived from distinct animal subjects per experimental group. Normality and variance homogeneity of the continuous datasets were verified using the Shapiro–Wilk test and Levene’s test, respectively. A two-way analysis of variance (ANOVA) with Sex (male vs. female) and Group (control vs. PTSD) as the two main factors was applied to evaluate behavioral, PET imaging, and biochemical datasets, with the systematic reporting of F values and corresponding degrees of freedom (d*f*). Sex-restricted hormones (testosterone and estradiol) were analyzed within single-sex cohorts using unpaired two-tailed Student’s *t*-tests. Pearson’s correlation analysis was utilized to evaluate the relationships between metabolic, behavioral, and molecular metrics. For all representative molecular graphics and imaging cross-sections lacking direct statistical plots, the presented snapshots were selected as typical profiles derived from multiple independent experimental replications exhibiting consistent biological trends. Statistical significance was defined a priori at *P* < 0.05.

## Results

### Behavioral and physiological changes

**Fig. 2:**
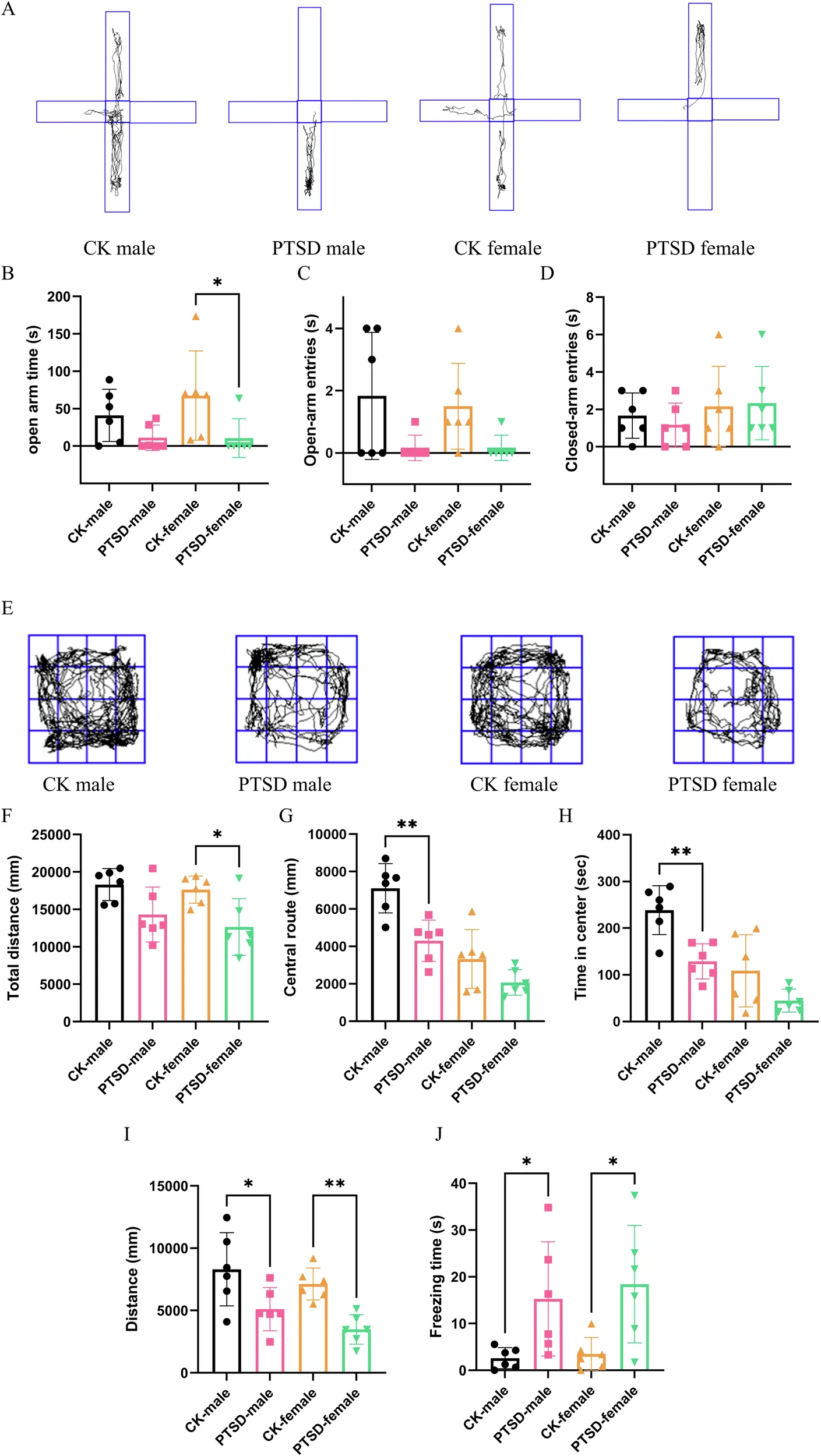
Sex-specific behavioral phenotypes in aged PTSD rats. Behavioral assessments of aged male and female rats (*n* = 6 per group). **A** Representative movement trajectories in the EPM. **B**–**D** Quantification of **B** percentage of time spent in open arms (OT%), **C** percentage of open arm entries (OE%), and **D** closed arm entries (CAE) in the EPM. **E** Representative trajectories in the OFT. **F**–**H** Quantification of **F** total distance traveled (TD), **G** central area distance (CR), and **H** time spent in the center (TIC) in the OFT. **I**, **J** FC test results: **I** distance traveled during fear learning and **J** freezing time during fear memory recall. Data are presented as mean ± SEM. *P < 0.05, **P < 0.01.

To evaluate the efficacy of the SPS + FS model and identify sex-specific vulnerabilities, a comprehensive battery of behavioral tests was performed (Fig. 2). Two-way ANOVA was utilized to dissect the main effects of Sex, Group (traumatic stress), and their Interaction.

Representative trajectories in the elevated plus maze (EPM) are shown in Fig. 2A. Anxiety-like behavior was assessed by the percentage of time spent in open arms (OT%) and the percentage of open arm entries (OE%). For OT% (Fig. 2B), statistical analysis showed that there was a significant main effect of Group (*F*_(1, 20)_ = 7.866, *P* = 0.0109), while the main effect of Sex (*F*_(1, 20)_ = 0.7025, *P* = 0.4118) and the Sex × Group interaction (*F*_(1, 20)_ = 0.7525, *P* = 0.3960) were non-significant. Despite the lack of a global stress effect, Tukey’s post-hoc analysis revealed that female PTSD rats showed a strong trend toward reduced open-arm time compared to their controls (*P* = 0.0750), a trend absent in male rats (*P* = 0.5317). Similarly, for OE% (Fig. 2C), a significant main effect of Group was observed (*F*_(1, 20)_ = 8.438, *P* = 0.0088). Importantly, analysis of closed arm entries (CAE, Fig. 2D) showed no significant effects for Group (*P* = 0.8103), Sex (P = 0.2380), or Interaction (*P* = 0.6319), confirming that the observed avoidance was not confounded by differences in general locomotor ability.

In the Open Field Test (OFT, Fig. 2E–H), traumatic stress significantly impacted exploratory behavior. For total distance traveled (TD, Fig. 2F), statistical analysis showed that there was a significant main effect of Group (*F*_(1, 20)_ = 13.65, *P* = 0.0014), while the Sex effect (*P* = 0.3492) and Interaction (*P* = 0.6922) were non-significant. Notably, post-hoc testing demonstrated that stress significantly reduced total distance only in female rats (CK-female vs. PTSD-female, *P* = 0.0409) but not in males (*P* = 0.1249). In contrast, central exploration indices showed robust Sex effects: for central route distance (Fig. 2G), there were significant main effects for Sex (*F*_(1, 20)_ = 36.73, *P* < 0.0001) and Group (*F*_(1, 20)_ = 16.74, *P* = 0.0006). Similarly, for time in center (TIC, Fig. 2H), significant main effects were found for Sex (*F*_(1, 20)_ = 25.51, *P* < 0.0001) and Group (*F*_(1, 20)_ = 16.81, *P* = 0.0006). These results indicate that traumatic stress induces consistent anxiety-like avoidance in rats, with females exhibiting unique locomotor sensitivity to the protocol.

Fear conditioning (FC) performance and memory recall were evaluated in Fig. 2I, J. For the distance traveled during learning (Fig. 2I), statistical analysis showed that there was a significant main effect of Group (*F*_(1, 20)_ = 19.08, *P* = 0.0003) and a strong trend for the Sex effect (*F*_(1, 20)_ = 3.221, *P* = 0.0878). Post-hoc comparisons confirmed that traumatic stress significantly impacted distance in both males (*P* = 0.0416) and females (*P* = 0.0177).

Finally, contextual fear memory was assessed via freezing time during the recall test (Fig. 2J). Statistical analysis showed that there was a significant main effect of Group (*F*_(1, 20)_ = 14.07, *P* = 0.0013), although the global main effect of Sex did not reach significance (*F*_(1, 20)_ = 0.3047, *P* = 0.5871). However, Tukey’s post-hoc analysis revealed that traumatic stress significantly increased freezing time specifically in female PTSD rats (CK-female vs. PTSD-female, *P* = 0.0433), whereas the increase in male rats was non-significant (*P* = 0.1022). Collectively, these behavioral data consistently demonstrate that female rats exhibit heightened susceptibility to traumatic stress, manifesting as more severe and persistent PTSD-like behavioral deficits, particularly regarding the intensity of fear memory retention.

### ELISA analysis

**Fig. 3:**
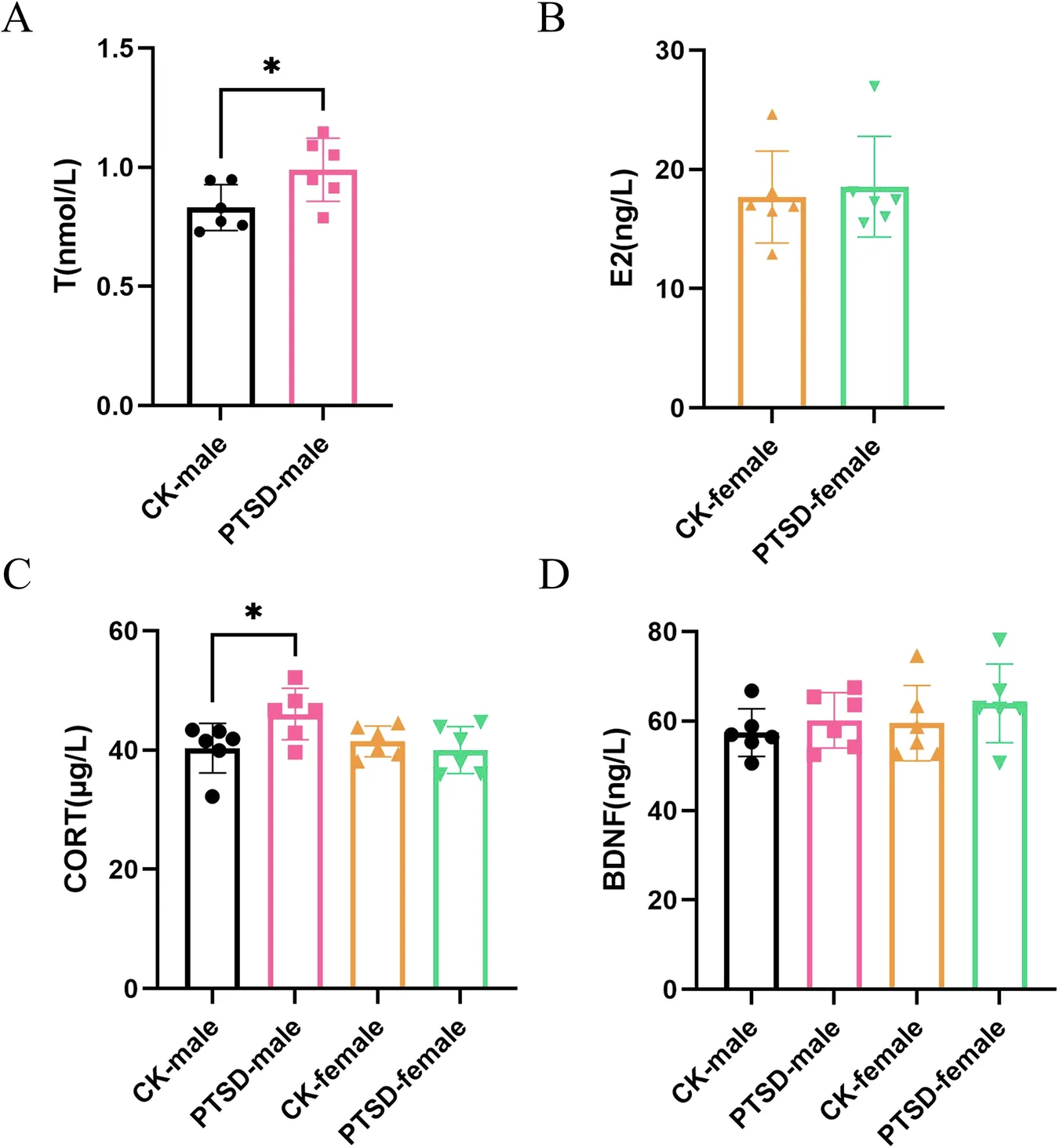
Systemic endocrine and neurotrophic factor profiles following traumatic stress. Plasma endocrine and biomarker profiles of rats (*n* = 6 per group). **A** Plasma testosterone (T) levels in male rats. **B** Plasma estradiol (E2) levels in female rats. **C** Plasma corticosterone (CORT) levels across the four experimental groups. **D** Circulating brain-derived neurotrophic factor (BDNF) levels in both male and female rats. Data are presented as mean ± SEM. *P < 0.05. Statistical significance was determined by unpaired Student’s *t*-test (**A**, **B**) or Two-way ANOVA followed by Tukey’s post-hoc test (**C**, **D**).

To assess systemic physiological shifts, we quantified plasma levels of stress hormones, gonadal steroids, and neurotrophic factors (Fig. 3).

Analysis of gonadal hormones, plasma testosterone (T) in males and estradiol (E_2_) in females, was evaluated independently (Fig. 3A, B). Due to the senescence-associated cessation of regular estrous cycling characteristic of 15-month-old female rats (typically representing a peri- or post-reproductive state) and the inherently negligible concentrations of cross-sex hormones (i.e., T in females and E_2_ in males) in this aged cohort, cross-sex comparisons and opposite-sex hormone assays were not performed. In males, traumatic exposure resulted in a significant elevation of plasma T levels (*t*_(10)_ = 2.384, *P* = 0.0383). Conversely, E_2_ levels in female rats remained stable following PTSD induction (*t*_(10)_ = 0.3749, *P* = 0.7156).

Regarding plasma Corticosterone (CORT) (Fig. 3C), a significant Sex × Group interaction (*F*(1, 20) = 5.260, *P* = 0.0328), indicating that the neuroendocrine response to traumatic stress was modulated by biological sex. Specifically, post-hoc comparisons revealed that CORT levels were notably elevated in male PTSD rats compared to their respective controls (*P* = 0.0762), whereas no significant stress-induced change was observed in female rats (*P* = 0.9125). Besides, systemic BDNF levels (Fig. 3D) were assessed across all experimental groups. Statistical analysis revealed no significant main effects for Group (*F*_(1, 20)_ = 1.414, *P* = 0.2483) or Sex (*F*_(1, 20)_ = 0.9674, *P* = 0.3371) and no significant Sex × Group interaction (*F*_(1, 20)_ = 0.08032, *P* = 0.7798). Although a slight numerical increase was noted in the PTSD groups compared to controls (62.08 ng/mL vs. 58.51 ng/mL), these differences did not reach statistical significance, indicating that peripheral BDNF levels in rats remain statistically stable following this traumatic paradigm.

### PET imaging quantitative analysis

**Fig. 4:**
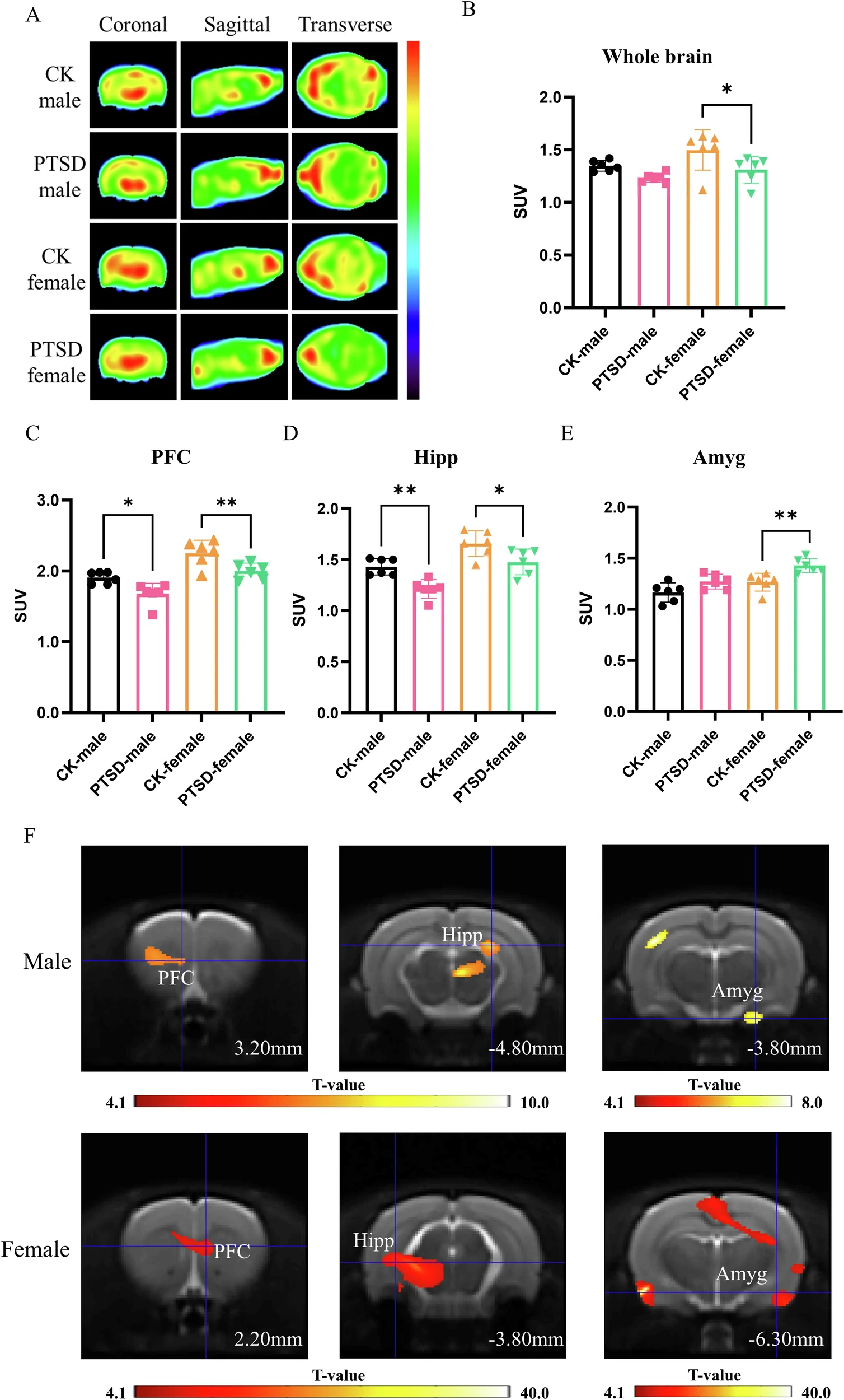
PET analysis of brain glucose metabolism in aged rats with sex-specific PTSD. **A** Representative coronal, sagittal, and transverse PET images of brain glucose metabolism; color coding indicates glucose metabolism levels (black = low, red = high). **B**–**E** Quantitative analysis of standardized uptake values (SUVs) in the whole brain, prefrontal cortex (PFC), hippocampus (Hipp), and amygdala (Amyg), respectively. **F** Voxel-wise statistical comparison of FDG uptake between PTSD and control rats (*T*-value = 4.1, *P* < 0.001 uncorrected); white numbers denote anteroposterior coordinates (Paxinos and Watson rat brain atlas). Data are expressed as mean ± SEM. * *P* < 0.05, ** *P* < 0.01.

To investigate the neurobiological basis of the observed behavioral phenotypes, we quantified regional brain glucose metabolism via FDG-PET. Qualitative assessment of the representative coronal, sagittal, and transverse planes (Fig. 4A) suggested a general reduction in radiotracer uptake following traumatic stress, a pattern that appeared more pronounced in the female cohort.

Quantitative analysis of whole-brain SUV (Fig. 4B) via statistical analysis showed a significant main effect of Group (*F*(_1, 20)_ = 9.643, *P* = 0.0056) and a significant main effect of Sex (*F*_(1, 20)_ = 5.420, *P* = 0.0305). While post-hoc comparisons indicated a notable trend toward whole-brain metabolic suppression primarily in females (*P* = 0.0576; Males: *P* = 0.3684), more profound disruptions were localized at the circuit level (Fig. 4C–E). Specifically, statistical analysis showed that there was for the PFC (Fig. 4C) a robust main effect of Sex (*F*_(1, 20)_ = 36.07, *P* < 0.0001) and a significant main effect of Group (*F*_(1, 20)_ = 18.55, *P* = 0.0003), with post-hoc testing confirming reduced metabolism in both female (*P* = 0.0223) and male PTSD rats (*P* = 0.0399). Similarly, for the Hipp (Fig. 4D), a significant Sex effect was observed (*F*_(1, 20)_ = 30.68, *P* < 0.0001), with both sexes exhibiting metabolic hypoactivity post-trauma (Females: *P* = 0.0441; Males: *P* = 0.0118). In contrast, the Amyg (Fig. 4E) exhibited a distinct metabolic divergence characterized by a significant main effect of Group (*F*_(1, 20)_ = 16.38, *P* = 0.0006). Crucially, post-hoc multiple comparisons revealed that traumatic stress-induced significant metabolic hyperactivation specifically in female PTSD rats (*P* = 0.0151), whereas no significant alteration was detected in males (*P* = 0.1876).

These voxel-level spatial differences are further intuitively illustrated in the statistical parameter maps and glass brain visualizations (Fig. 4F, Supplementary Fig. 2 and Supplementary Fig. 3), with the specific coordinates and cluster details of the metabolic alterations systematically documented in Table S2 (males) and Table S3 (females).

### Dynamic PET imaging and glucose metabolic rate analysis

**Fig. 5:**
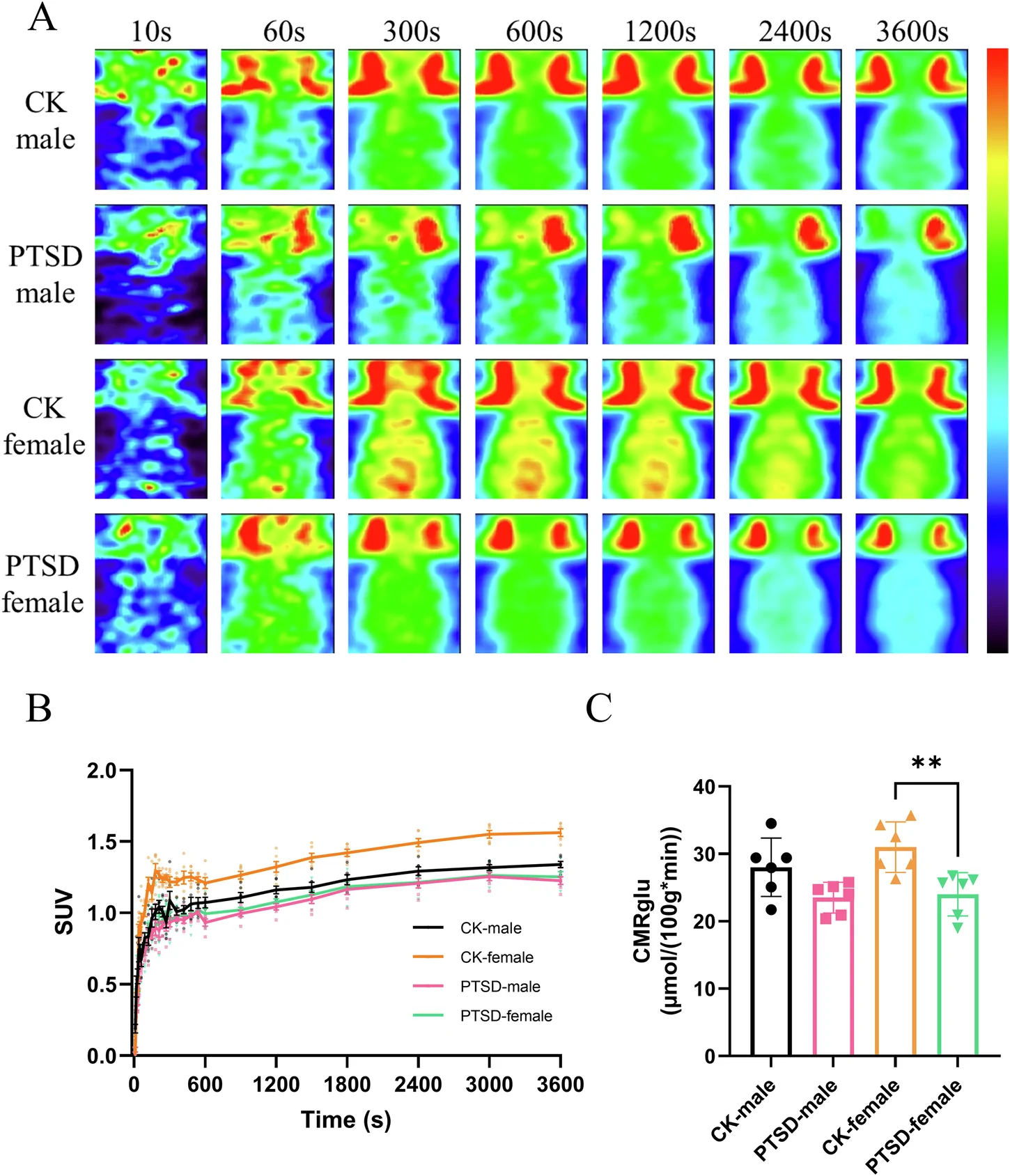
Sex differences in brain glucose metabolism of PTSD rats: dynamic PET analysis. **A** Representative dynamic PET image displays changes in coronal cerebral glucose metabolism at multiple time points (10 s, 60 s, 300 s, 600 s, 1200 s, 2400 s, 3600 s) over the 60-min scan for all four rat groups. Image color coding indicates glucose metabolism levels. **B** Curves illustrate time-dependent changes in the whole-brain glucose standardized uptake value (SUV) for each rat group during the 60-min dynamic scan. These curves visually demonstrate how the radiotracer is taken up and reaches equilibrium in the brain. **C** Quantitative analysis presents the whole-brain glucose metabolic rate (CMRglu) for each rat group. This metric reflects the steady-state level of glucose consumption in the brain. Data are presented as mean ± SEM. *P < 0.05.

To capture the temporal dynamics of brain glucose utilization, we analyzed the time–activity curves (TACs) and calculated the cerebral glucose metabolic rate (CMRglu). The dynamic sequence images (Fig. 5A) and the corresponding whole-brain TACs (Fig. 5B) captured the specific temporal kinetics of tracer uptake across the Control and PTSD groups of both sexes. Two-way repeated measures ANOVA for the TACs identified a highly significant effect of Time (*F*_(25, 520)_ = 208.4, *P* < 0.0001) and Group (*F*_(3, 520)_ = 228.4, *P* < 0.0001). Qualitative assessment indicated that the female control group maintained the highest overall uptake levels, while traumatic stress shifted the curves downward in both sexes. This suggests a sustained state of cerebral hypometabolism following trauma, with the divergence being more visually apparent in the female cohort.

Further CMRglu quantification (Fig. 5C) confirmed a significant stress-induced metabolic decline (Main effect of Group: *F*_(1, 20)_ = 16.42, *P* = 0.0006). At baseline, although female controls exhibited a slight numerical elevation in cerebral energy metabolism compared to male controls, the difference did not reach statistical significance (*P* = 0.4636). Regarding PTSD-induced alterations, while male PTSD rats exhibited a downward trend in CMRglu compared to their respective controls, this change did not reach statistical significance (*P* = 0.1450). In contrast, female PTSD rats exhibited a more pronounced reduction in whole-brain CMRglu, with statistical significance (*P* = 0.0115).

### Metabolic-behavioral correlation analysis

**Fig. 6:**
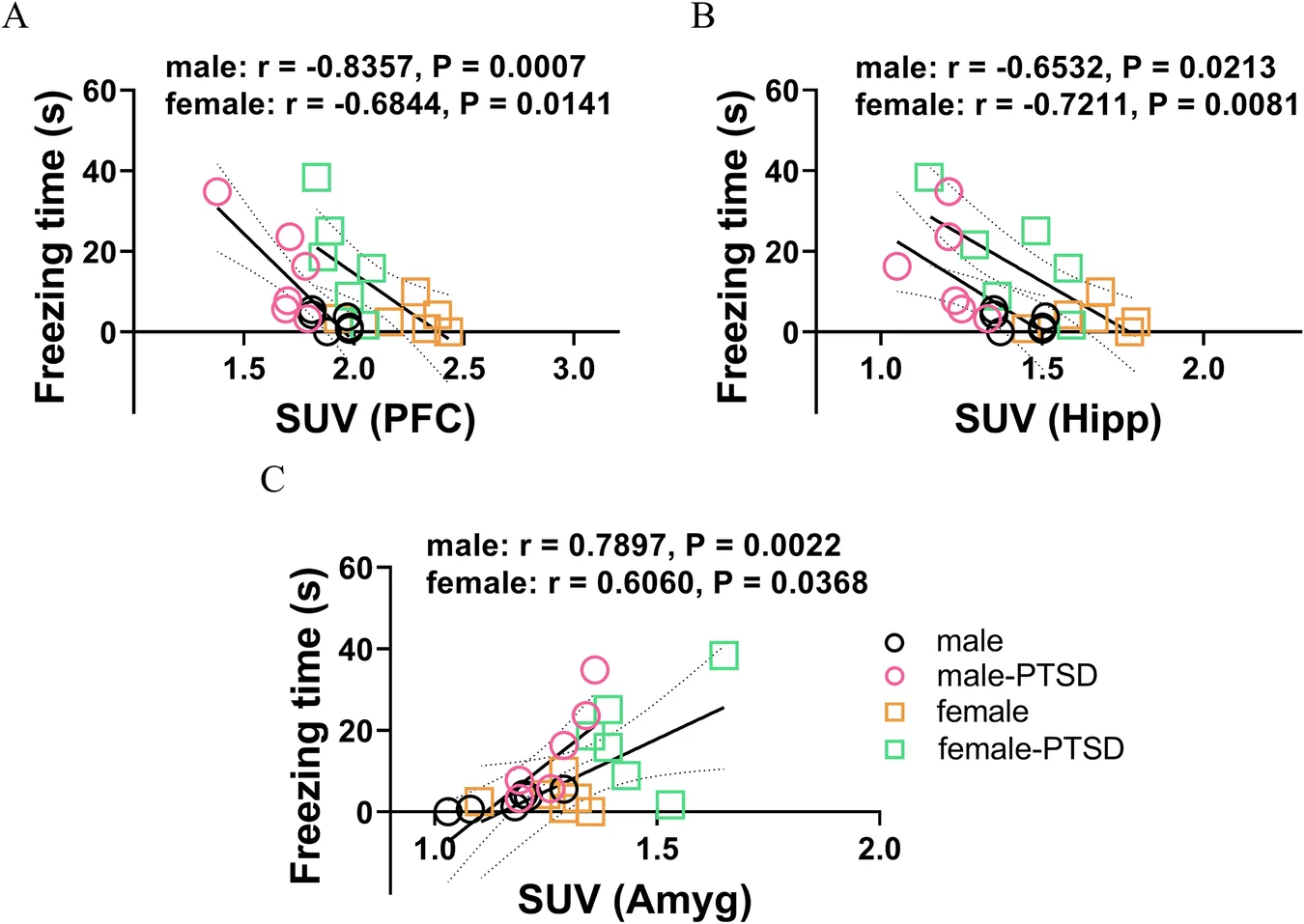
Pearson correlation analysis between regional brain glucose metabolism and fear memory deficits in aged rats. **A**–**C** Quantitative correlation analysis between standardized uptake values (SUVs) and freezing time in the PFC (**A**), Hipp (**B**), and Amyg (**C**). Significant correlations between regional metabolic activity and freezing behavior were identified in the PFC, Hipp, and Amyg across both sexes. Pearson’s correlation coefficients (*r*) and *P*-values for each sex are indicated within the respective panels. Solid lines represent linear regression, and dotted lines indicate the 95% confidence intervals. Data from control and PTSD groups within each sex were pooled to capture the full spectrum of metabolic and behavioral variation (*n* = 12 per sex).

To determine the functional relevance of the observed metabolic dysregulation, we performed correlation analyses between regional SUV values and freezing behavior. In the PFC (Fig. 6A) and Hipp (Fig. 6B), metabolic activity exhibited a robust negative correlation with freezing time in both sexes (PFC: Male *r* = -0.83, *P* < 0.001, Female *r* = -0.68, *P* < 0.05; Hipp: Male *r* = -0.65, *P* < 0.05, Female *r* = -0.72, *P* < 0.01). Conversely, metabolic activity in the amygdala (Fig. 6C) showed a significant positive correlation with freezing time (Male *r* = 0.78, *P* < 0.01; Female *r* = 0.60, *P* < 0.05). Notably, no significant correlations were observed between non-core metrics, such as PFC SUV and open-arm time, or whole-brain CMRglu and freezing behavior (Supplementary Fig. 4).

### Transcriptomic analysis

#### GO enrichment analysis of differentially expressed genes

**Fig. 7:**
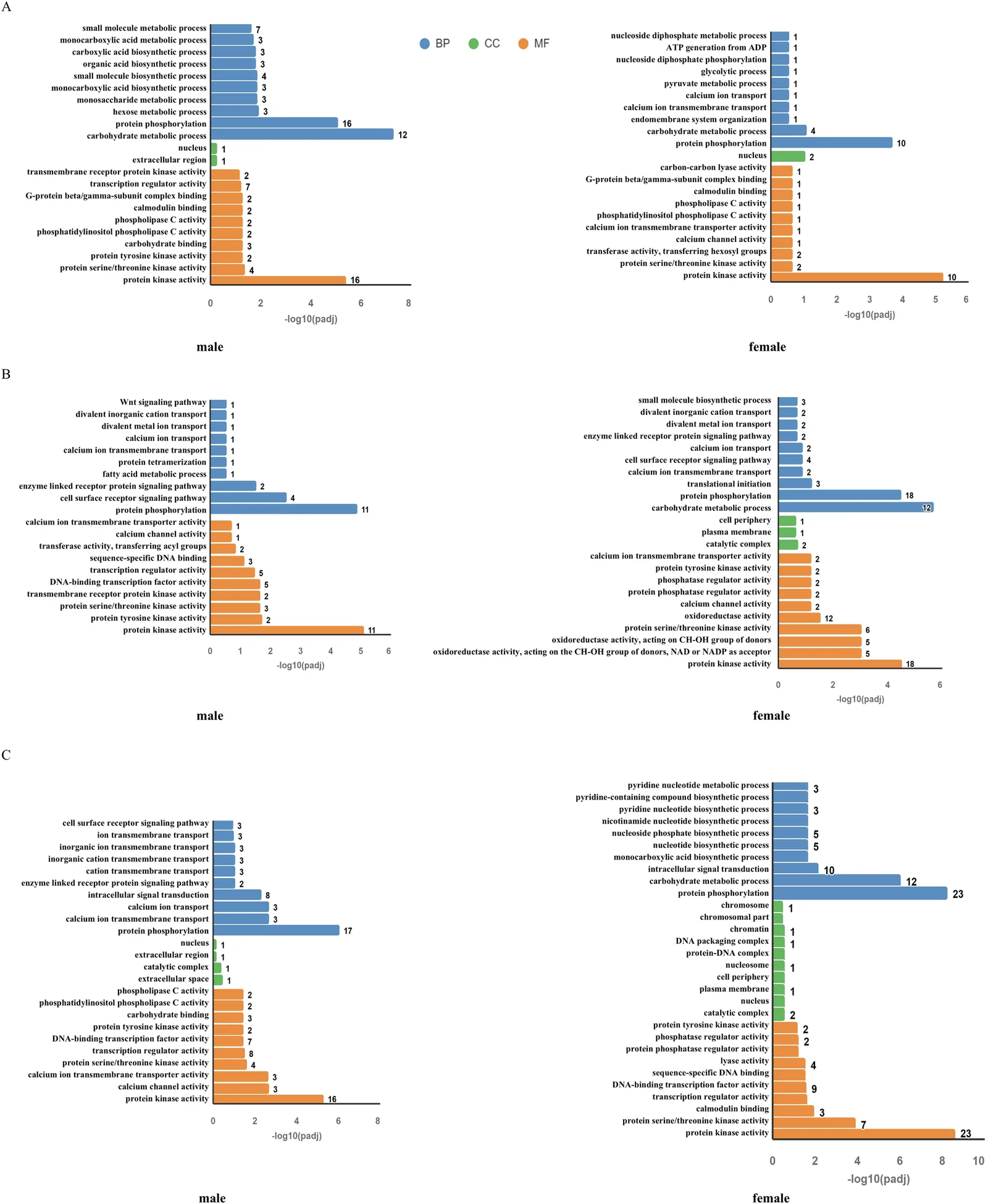
GO functional enrichment analysis of genes related to different brain regions of PTSD model rats. **A**–**C** Enrichment results in the PFC (**A**), Hipp (**B**), and Amyg (**C**). The analysis incorporates differentially expressed genes from both male and female samples, categorized into biological process (BP), cellular component (CC), and molecular function (MF). The *x*-axis represents −log_10_(pad*j*), where larger values indicate more significant enrichment.

As illustrated in Fig. 7, GO functional enrichment analysis revealed spatial heterogeneity in the distribution of metabolism-related genes across distinct brain regions and sex-specific cohorts.

Specifically, in the PFC (Fig. 7A), DEGs in both males and females were predominantly enriched in biological processes (BP) such as protein phosphorylation and carbohydrate metabolic processes, alongside molecular functions (MF) including protein kinase activity. Regarding the Hipp (Fig. 7B), both sex-specific cohorts displayed a pronounced enrichment in protein phosphorylation and transmembrane transporter activity, with females additionally exhibiting specific enrichment associated with the cell periphery (CC). In the Amyg (Fig. 7C), both groups maintained high significance in intracellular signal transduction and protein kinase activity.

Across the examined regions, the enrichment profiles consistently involved cellular components and molecular functions related to kinase-driven signaling and energy regulation. The degree of enrichment varied by region and sex, with the most prominent metabolic enrichment observed in the PFC for the male cohort and in the amygdala for the female cohort.

#### KEGG pathway enrichment analysis of differentially expressed genes

**Fig. 8:**
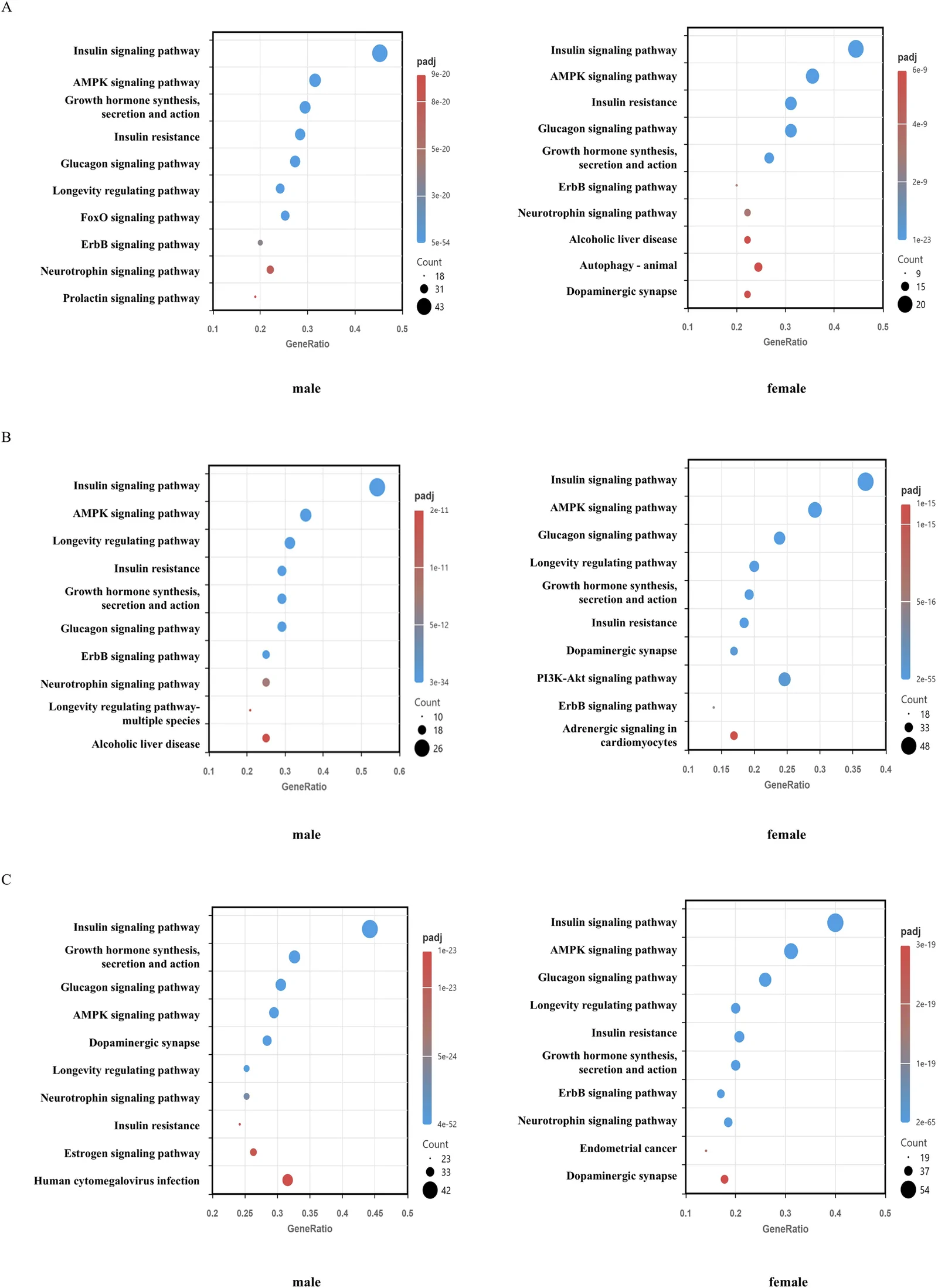
KEGG pathway enrichment analysis for differentially expressed genes across distinct brain regions in traumatic stress model rats. **A**–**C** KEGG enrichment profiles in the PFC (**A**), Hipp (**B**), and Amyg (**C**). The analysis incorporates differentially expressed genes from both male and female samples. The size of each dot represents the number of DEGs in the corresponding pathway, and the color indicates the enrichment significance (pad*j*).

As illustrated in Fig. 8, KEGG pathway enrichment analysis revealed distinct sex- and brain region-specific regulatory profiles across the three examined areas. Across all experimental cohorts, the “Insulin signaling pathway” consistently displayed the highest degree of enrichment.

In the PFC (Fig. 8A), both male and female cohorts exhibited significant enrichment in the insulin, AMPK, and glucagon signaling pathways, alongside pathways associated with insulin resistance. This pattern of metabolic pathway enrichment was largely maintained in the Hipp (Fig. 8B) and Amyg (Fig. 8C), where the insulin signaling pathway ranked at the top of the enrichment profiles in terms of both significance and gene ratio.

Additionally, secondary networks, such as dopaminergic synapses and longevity-regulating pathways, showed varying degrees of enrichment that differed by sex and brain region. In the female hippocampus, for instance, a notable enrichment in the PI3K–Akt signaling pathway was observed, while the male amygdala showed relatively higher significance in pathways related to estrogen signaling.

#### Protein–protein interaction (PPI) network and differential gene clustering

**Fig. 9:**
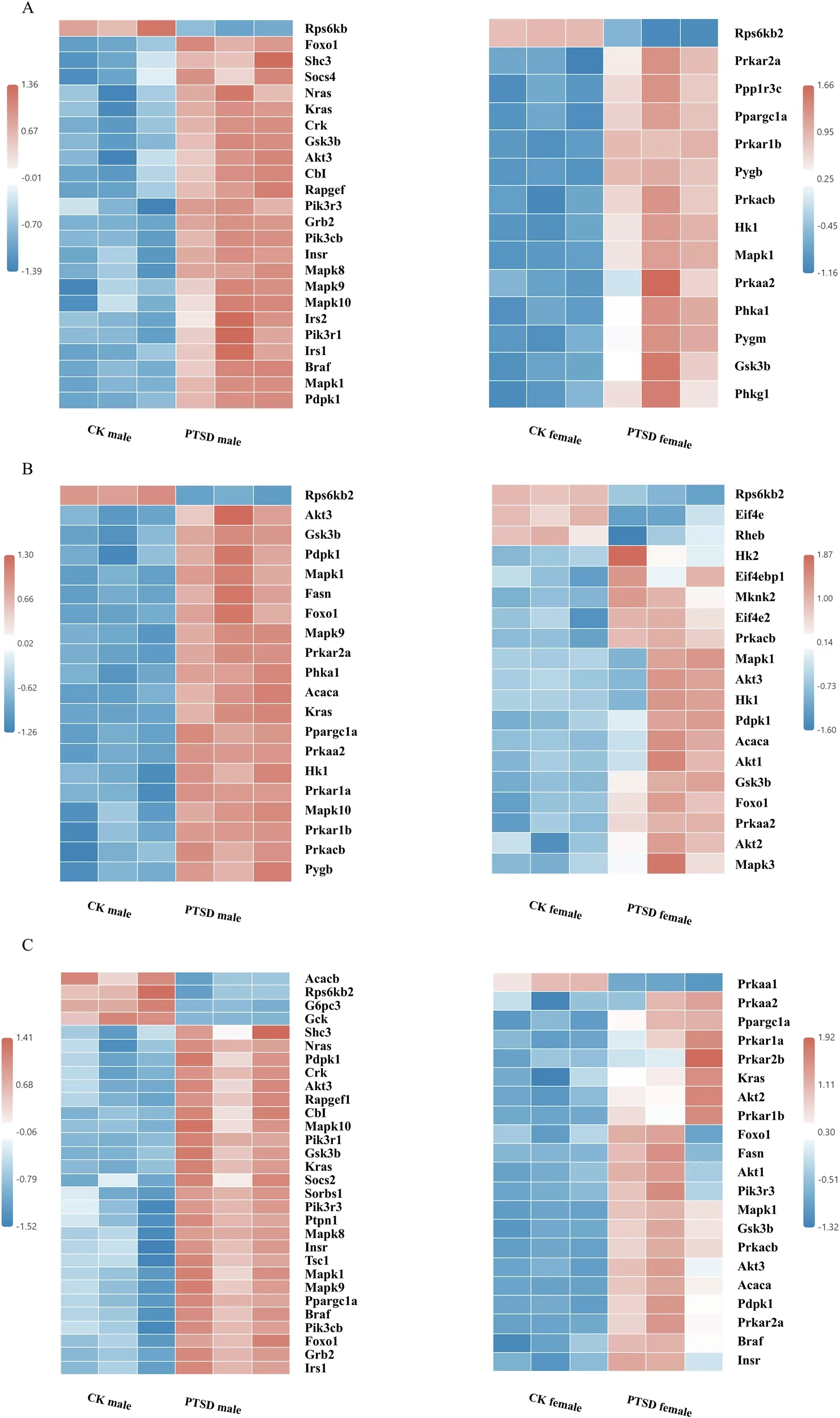
Expression clusters of core genes across distinct brain regions in traumatic stress model rats. **A**–**C** Heatmaps illustrating differential expression patterns of core genes in the PFC (**A**), Hipp (**B**), and Amyg (**C**). Within each individual heatmap, the dataset is stratified by biological sex, sequentially displaying male groups and female groups as designated by the horizontal axis labels. The color scale bars define the expression levels, utilizing a color gradient from blue to red to represent the transitions from downregulated to upregulated gene expression z-scores.

Based on the pronounced and universal enrichment of the insulin signaling pathway identified in the KEGG analysis, genes mapped to this specific cascade were extracted to construct targeted protein–protein interaction (PPI) networks (Supplementary Fig. 5). Topological clustering within these PPI networks successfully isolated the core hub genes orchestrating the insulin signaling network, encompassing essential receptors and adaptors (e.g., *Insr*, *Irs1/2*), critical regulatory kinases (e.g., *Akt* isoforms, *Pik3* subunits, *Gsk3b*, *Mapk* family), and downstream metabolic effectors and regulators (e.g., *Hk1*, *Pygb*, *Ppargc1a*). Figure 9 illustrates the expression heatmaps of these identified hub genes. Although the core insulin signaling network was targeted in both sexes, the precise expression patterns of these hub genes exhibited spatial and sex-dependent divergence. In the PFC (Fig. 9A), Hipp (Fig. 9B), and Amyg (Fig. 9C), male and female cohorts displayed distinct up and downregulatory profiles among specific kinase cascades and metabolic enzymes, highlighting a divergent transcriptional response within the identical metabolic framework following trauma.

## Discussion

By integrating macro-scale neuroimaging with transcriptomics, this study offers a potential framework for understanding the neuroenergetic basis of sex-specific PTSD phenotypes in the aged brain. Our observations suggest that 15-month-old female rats may exhibit heightened behavioral vulnerability, potentially linked to pronounced regional metabolic dysregulation and molecular shifts that appear to function independently of peripheral hormonal status.

Several preclinical animal studies have demonstrated a precise correlation between behavioral impairments and metabolic imbalances in brain regions^17,40,41^. This association is also evident in our PTSD rat model. As shown in Fig. 2, PTSD rats exhibited typical anxiety-like behaviors, hypervigilance, and decreased exploratory behavior. These behavioral phenotypes are closely associated with the dysregulation of stress endocrine pathways, which may subsequently impact synaptic function and neuroplasticity. Significant sex differences exist in this dysregulation. Regarding the core stress hormone corticosterone (CORT), plasma CORT levels were significantly elevated in male PTSD rats (Fig. 3C). Combined with previous research^42^, this change suggests a hyper-responsive hypothalamic–pituitary–adrenal (HPA) axis in males. Unlike males, no significant differences in plasma CORT levels were observed in female PTSD rats. Previous studies suggest that older female rats are relatively resilient to chronic stress and better able to maintain stable levels of CORT and gonadal steroids; this may account for the lack of significant CORT fluctuations during PTSD^43^. In addition to stress hormones, the body attempts to promote neuronal repair and survival by increasing systemic circulating brain-derived neurotrophic factor (BDNF) following traumatic stress^44^. This is consistent with our observation of elevated BDNF levels in both male and female PTSD rats (Fig. 3D). Notably, while estrogen is conventionally recognized for its neuroprotective properties in younger cohorts^45^, this protective efficacy appears to diminish significantly in the aged brain due to reproductive senescence and the potential downregulation of estrogenic signaling pathways. In this study, the stable but low serum estradiol (E2) levels (Fig. 3B) in female PTSD rats failed to buffer against traumatic injury, likely reflecting an age-related attenuation of classical neuroprotection. This contrasts with the significant elevation of testosterone observed in males (Fig. 3A), which may represent a compensatory adaptive response aimed at mitigating traumatic impacts^46^. This divergence in peripheral endocrine flexibility further underscores the unique bioenergetic vulnerability of the aged female brain.

Beyond peripheral endocrine shifts, our neuroimaging data revealed a characteristic pattern of metabolic dysregulation within the fear circuitry, particularly in the female cohort. The observed hypoactivity in the PFC and hippocampus coupled with amygdala hypermetabolism (Fig. 4) aligns with the core behavioral phenotypes observed in our PTSD model. In this framework, the PFC serves as a high-level cognitive center, and its diminished metabolic state likely reflects a failure in top-down inhibitory control over fear responses. This functional link is further supported by our Pearson correlation analysis (Fig. 6), which identified a robust negative coupling between PFC and hippocampal metabolism and freezing duration. Conversely, the amygdala acts as the emotional alarm system of the brain. Its hypermetabolic state positively correlated with the severity of anxiety-like manifestations (Fig. 6C) and suggests an overactive substrate driving excessive fear responses^47^. Furthermore, the metabolic suppression within the hippocampus may be associated with impaired consolidation and retrieval of traumatic memories^48,49^. These divergent correlation patterns indicate that the metabolic integrity of this specific PFC–Hipp–Amyg circuit, rather than global brain energetics, serves as a primary determinant of PTSD-like symptomatology. While our dynamic PET analysis also identified a significant decrease in the whole-brain cerebral glucose metabolic rate (CMRglu) (Fig. 5C), which is consistent with clinical reports of global metabolic decline in human PTSD patients^16^, the localized disruptions within the fear-regulatory network appear to be the more direct predictors of the observed behavioral alterations.

The regional alterations in cerebral glucose metabolism observed via FDG-PET imaging appear to be intimately linked to the dysregulation of the central insulin and AMPK signaling networks. In the central nervous system, beyond facilitating basic glucose uptake, insulin signaling acts as a profound modulator of neuroenergetics, synaptic plasticity, and neuronal survival^50–52^. While multi-species transcriptomic meta-analyses confirm that PTSD is characterized by a conserved disruption of bioenergetic and synaptic pathways^53^, and hippocampal-specific profiling has further linked these transcriptional shifts directly to PTSD-like behavioral deficits^54^, our data in 15-month-old rats uniquely underscore a systemic reprogramming of these metabolic signaling cascades. Specifically, we identified a coordinated response among key nodes within the insulin-AMPK axis. Mechanistically, the dysregulation of the glycolytic rate-limiting enzyme *Hk1* likely reflects an adaptive attempt to accelerate glycolytic flux, potentially to compensate for trauma-induced mitochondrial insufficiency^23,55,56^. This is further supported by alterations in *Ppargc1a*, a master regulator of mitochondrial biogenesis, reflecting cellular efforts to recalibrate the balance between glycolysis and oxidative phosphorylation^57^. At the signaling level, *Gsk3b* acts as a central metabolic-behavioral nexus. As a primary downstream inhibitor of the insulin cascade, its overactivation is a recognized hallmark of neuronal insulin resistance and drives a blockade of glycogen synthesis via the inhibitory phosphorylation of glycogen synthase^58^. Parallel to this, alterations in the brain-type glycogen phosphorylase *Pygb* reflect a prioritized mobilization of existing glycogen reserves to bypass impaired oxidative pathways^59^. This dual shift redirects glucose substrates toward immediate glycolysis to meet the urgent energy demands under mitochondrial strain. Such a state of “metabolic emergency” is further corroborated by the involvement of *Prkaa2* (AMPKα2), a critical energy sensor that stimulates downstream catabolism upon detecting cellular ATP deficits^60^. Significantly, the biological cost of this metabolic redirection appears to be sexually dimorphic. While this overarching insulin and AMPK framework is universally targeted by traumatic stress, the adaptive strategies within this network exhibit profound sex-dependent spatial heterogeneity. Rather than utilizing completely divergent pathways, males and females demonstrate distinct regulatory “tuning” of specific kinase subunits and metabolic enzymes within the identical signaling cascade. In female rats, this transcriptional tuning likely reflects a more exhaustive compensatory mode. In this state, the accelerated depletion of metabolic reserves may lead to earlier synaptic compromise and lower the threshold for behavioral dysfunction.

In conclusion, our multimodal study highlights a potential link between the observed behavioral phenotypes and central metabolic alterations in the 15-month-old rat model of PTSD. Female rats exhibited more pronounced PTSD-like manifestations, which appeared to be independent of major peripheral hormone fluctuations. Macroscopically, FDG-PET imaging revealed widespread glucose metabolic changes across the core fear circuitry. At the molecular level, targeted transcriptomics suggests that these neuroenergetic shifts are closely associated with the modulation of central insulin and AMPK signaling networks. While this overarching metabolic response involving key hubs such as *Gsk3b*, *Hk1*, and *Prkaa2* was observed in both sexes, the specific transcriptional patterns exhibited notable sex-dependent spatial heterogeneity. Despite these findings, certain limitations of the current study must be acknowledged. The reliance on bulk tissue transcriptomics and a cross-sectional design restricts our ability to resolve cell-type-specific contributions and dynamic longitudinal changes. Furthermore, the identified molecular pathways warrant future protein-level functional validation. Ultimately, addressing these limitations in future research will further elucidate the underlying neurobiological mechanisms and aid in the development of precision, sex-specific therapeutic strategies for PTSD.

## Supplementary information

Transparent Peer Review file

Supplementary Information

Supplementary materials description

Supplementary Data 1

nr-reporting-summary

## Data Availability

The RNA-sequencing datasets (both raw reads and processed quantification files) generated during this study have been mandatorily deposited in the Gene Expression Omnibus (GEO) repository under accession number GSE327211. Other foundational raw tracking data supporting the statistical findings, including longitudinal behavioral matrices and subregional micro-PET neuroimaging datasets, are available from the corresponding author upon reasonable request. The complete individual continuous datasets and raw numerical source data underlying all quantitative graphs and charts presented within the main figures (Figs. 2–6) and Supplementary Figs. (Supplementary Fig. 4A, B) are fully disclosed within Supplementary Data 1.

## References

[1] Villamor, A. & Sáez de Adana, E. in *Psychopathology in Women: Incorporating Gender Perspective into Descriptive Psychopathology*. (Springer, 2014), pp 587–609.

[2] Yehuda, R. et al. Post-traumatic stress disorder. *Nat. Rev. Dis. Prim.* **1**, 15057 (2015). 10.1038/nrdp.2015.57 27189040

[3] Ramikie, T. S. & Ressler, K. J. Mechanisms of sex differences in fear and posttraumatic stress disorder. *Biol. Psychiatry* **83**, 876–885 (2018). 10.1016/j.biopsych.2017.11.016 29331353

[4] Olff, M. Sex and gender differences in post-traumatic stress disorder: an update. *Eur. J. Psychotraumatol.* **8**, 1351204 (2017).

[5] Cook, J. M. & Simiola, V. Trauma and PTSD in older adults: prevalence, course, concomitants and clinical considerations. *Curr. Opin. Psychol.* **14**, 1–4 (2017). 10.1016/j.copsyc.2016.08.003 28813305

[6] Van Zelst, W. H., de Beurs, E., Beekman, A. T., Deeg, D. J. & van Dyck, R. Prevalence and risk factors of posttraumatic stress disorder in older adults. *Psychother. Psychosom.* **72**, 333–342 (2003). 10.1159/000073030 14526136

[7] Baltjes, F., Cook, J. M., van Kordenoordt, M. & Sobczak, S. Psychiatric comorbidities in older adults with posttraumatic stress disorder: a systematic review. *Int. J. Geriatr. Psychiatry* **38**, e5947 (2023). 10.1002/gps.5947 37303126

[8] Shin, L. M. & Liberzon, I. The neurocircuitry of fear, stress, and anxiety disorders. *Neuropsychopharmacology* **35**, 169–191 (2010). 10.1038/npp.2009.83 PMC3055419 19625997

[9] Alexandra Kredlow, M., Fenster, R. J., Laurent, E. S., Ressler, K. J. & Phelps, E. A. Prefrontal cortex, amygdala, and threat processing: implications for PTSD. *Neuropsychopharmacology* **47**, 247–259 (2022). 10.1038/s41386-021-01155-7 PMC8617299 34545196

[10] Bukalo, O. et al. Prefrontal inputs to the amygdala instruct fear extinction memory formation. *Sci. Adv.* **1**, e1500251 (2015). 10.1126/sciadv.1500251 PMC4618669 26504902

[11] Etkin, A. & Wager, T. D. Functional neuroimaging of anxiety: a meta-analysis of emotional processing in PTSD, social anxiety disorder, and specific phobia. *Am. J. Psychiatry* **164**, 1476–1488 (2007). 10.1176/appi.ajp.2007.07030504 PMC3318959 17898336

[12] de Araujo Junior, D. A. et al. The association between post-traumatic stress disorder (PTSD) and cognitive impairment: a systematic review of neuroimaging findings. *J. Psychiatr. Res.* **164**, 259–269 (2023). 10.1016/j.jpsychires.2023.06.016 37390621

[13] Isaac, C. L., Cushway, D. & Jones, G. V. Is posttraumatic stress disorder associated with specific deficits in episodic memory? *Clin. Psychol. Rev.* **26**, 939–955 (2006). 10.1016/j.cpr.2005.12.004 16481082

[14] Buchsbaum, M. S., Simmons, A. N., DeCastro, A., Farid, N. & Matthews, S. C. Clusters of low 18F-fluorodeoxyglucose uptake voxels in combat veterans with traumatic brain injury and post-traumatic stress disorder. *J. Neurotrauma* **32**, 1736–1750 (2015). 10.1089/neu.2014.3660 25915799

[15] Yehuda, R. et al. Changes in relative glucose metabolic rate following cortisol administration in aging veterans with posttraumatic stress disorder: an FDG-PET neuroimaging study. * J. Neuropsychiatry Clin. Neurosci.* **21**, 132–143 (2009). 10.1176/jnp.2009.21.2.132 19622684

[16] Zandieh, S. et al. Analysis of the metabolic and structural brain changes in patients with torture-related post-traumatic stress disorder (TR-PTSD) using 18F-FDG PET and MRI. *Medicine* **95**, e3387 (2016). 10.1097/MD.0000000000003387 PMC4839854 27082610

[17] Zhu, Y. et al. PET mapping of neurofunctional changes in a posttraumatic stress disorder model. *J. Nucl. Med.* **57**, 1474–1477 (2016). 10.2967/jnumed.116.173443 26985058

[18] Pinna, G., Ponomareva, O., Stalcup, G. L. & Rasmusson, A. M. Neuroactive steroids and the pathophysiology of PTSD: biomarkers for treatment targeting. *Neurosci. Biobehav. Rev.* **172**, 106085 (2025). 10.1016/j.neubiorev.2025.106085 PMC12160560 40024353

[19] Nisar, S. et al. Genetic and neuroimaging approaches to understanding post-traumatic stress disorder. *Int. J. Mol. Sci.* 10.3390/ijms21124503 (2020). PMC7352752 32599917

[20] Prasad, A., Chaichi, A., Kelley, D. P., Francis, J. & Gartia, M. R. Current and future functional imaging techniques for post-traumatic stress disorder. *RSC Adv.* **9**, 24568–24594 (2019). 10.1039/c9ra03562a PMC9069787 35527877

[21] Reis, A. L. M. et al. Sex-specific transcriptomic and epitranscriptomic signatures of PTSD-like fear acquisition. *iScience* **25**, 104861 (2022). 10.1016/j.isci.2022.104861 PMC9418440 36039298

[22] Xie, G., Qin, Y., Wu, N., Han, X. & Li, J. Single-nucleus transcriptome profiling from the hippocampus of a PTSD mouse model and CBD-treated cohorts. *Genes*10.3390/genes15040519 (2024). PMC11050643 38674453

[23] Amorim, F. E., Rye, C. S. & Milton, A. L. Mitochondrial dysfunction in PTSD: A mechanism to understand trauma susceptibility? *Psychopharmacology*10.1007/s00213-025-06900-0 (2025). PMC13242461 41071211

[24] Plum, L. & Belgardt, B. F. & Brüning, J. C. Central insulin action in energy and glucose homeostasis. * J. Clin. Investig.* **116**, 1761–1766 (2006). 10.1172/JCI29063 PMC1483153 16823473

[25] Garcia, D. & Shaw, R. J. AMPK: mechanisms of cellular energy sensing and restoration of metabolic balance. *Mol. Cell* **66**, 789–800 (2017). 10.1016/j.molcel.2017.05.032 PMC5553560 28622524

[26] Wang, J., Zhao, H. & Girgenti, M. J. Posttraumatic stress disorder brain transcriptomics: convergent genomic signatures across biological sex. *Biol. Psychiatry* **91**, 6–13 (2022). 10.1016/j.biopsych.2021.02.012 33840456

[27] Chang, S. H., Chen, H. Y., Shaw, F. Z. & Shyu, B. C. Early- and late-phase changes of brain activity and early-phase neuromodulation in the posttraumatic stress disorder rat model. *Neurobiol. Stress* **26**, 100554 (2023). 10.1016/j.ynstr.2023.100554 PMC10415797 37576348

[28] Carter, M. & Shieh, J. C. in *Guide to Research Techniques in Neuroscience* (eds Matt Carter & Jennifer C. Shieh) pp 39–71 (Academic Press, 2010).

[29] Pawlak, C. R., Karrenbauer, B. D., Schneider, P. & Ho, Y.-J. The elevated plus-maze test: differential psychopharmacology of anxiety-related behavior. *Emot. Rev.* **4**, 98–115 (2012).

[30] Russo, A. S. & Parsons, R. G. Behavioral expression of contextual fear in male and female rats. *Front. Behav. Neurosci.* **15**, 671017 (2021). 10.3389/fnbeh.2021.671017 PMC8249797 34220462

[31] Parkinson, F. E., Paul, S., Zhang, D., Mzengeza, S. & Ko, J. H. The effect of endogenous adenosine on neuronal activity in rats: an FDG PET study. *J. Neuroimaging* **26**, 403–405 (2016). 10.1111/jon.12349 PMC5021151 27082948

[32] Tang, Y. et al. The evaluation of dynamic FDG-PET for detecting epileptic foci and analyzing reduced glucose phosphorylation in refractory epilepsy. *Front. Neurosci.* **12**, 2018 (2019). 10.3389/fnins.2018.00993 PMC6333859 30686968

[33] Yang, M. S., Xu, X. J., Zhang, B., Niu, F. & Liu, B. Y. Comparative transcriptomic analysis of rat versus mouse cerebral cortex after traumatic brain injury. *Neural Regen. Res.* **16**, 1235–1243 (2021). 10.4103/1673-5374.301028 PMC8284282 33318400

[34] Dergunova, L. V. et al. Genome-wide transcriptome analysis using RNA-Seq reveals a large number of differentially expressed genes in a transient MCAO rat model. *BMC Genomics* **19**, 655 (2018). 10.1186/s12864-018-5039-5 PMC6125876 30185153

[35] Rediti, M. et al. Integrating molecular imaging and transcriptomic profiling in advanced HER2-positive breast cancer receiving Trastuzumab Emtansine: an analysis of the ZEPHIR clinical trial. *Clin. Cancer Res.* **31**, 110–121 (2025). 10.1158/1078-0432.CCR-24-1007 39470686

[36] Zhang, Y., Liu, Y., Meng, W., Yu, X. & Xu, X. Integrative transcriptomic analysis reveals microglial metabolic-inflammatory crosstalk of HK2-HSPA5-TNF axis after intracerebral hemorrhage. *Front. Bioinforma.* **5**, 1740715 (2025). 10.3389/fbinf.2025.1740715 PMC12833071 41601478

[37] Liu, C. et al. Protein-protein interaction networks and different clustering analysis in Burkitt’s lymphoma. *Hematology* **23**, 391–398 (2018). 10.1080/10245332.2017.1409947 29189103

[38] Ruderman, N. B., Carling, D., Prentki, M. & Cacicedo, J. M. AMPK, insulin resistance, and the metabolic syndrome. * J. Clin. Investig.* **123**, 2764–2772 (2013). 10.1172/JCI67227 PMC3696539 23863634

[39] Li, Y. et al. Integrating causal human genetics and in vivo transcriptomics to uncover a shared lipid-centric architecture in metabolic and neurocognitive disease. *Front. Mol. Biosci.* **12**, 1712198 (2025). 10.3389/fmolb.2025.1712198 PMC12887701 41676339

[40] Muhie, S. et al. Molecular signatures of post-traumatic stress disorder in war-zone-exposed veteran and active-duty soldiers. *Cell Rep. Med.* **4**, 101045 (2023). 10.1016/j.xcrm.2023.101045 PMC10213980 37196634

[41] Luft, C. et al. Sex differences in the effects of acute stress on cerebral glucose metabolism: a microPET study. *Brain Res.* **1722**, 146355 (2019). 10.1016/j.brainres.2019.146355 31356782

[42] Notaras, M. & van den Buuse, M. Neurobiology of BDNF in fear memory, sensitivity to stress, and stress-related disorders. *Mol. Psychiatry* **25**, 2251–2274 (2020). 10.1038/s41380-019-0639-2 31900428

[43] Bowman, R. E., Maclusky, N. J., Diaz, S. E., Zrull, M. C. & Luine, V. N. Aged rats: sex differences and responses to chronic stress. *Brain Res.* **1126**, 156–166 (2006). 10.1016/j.brainres.2006.07.047 16919611

[44] Colucci-D’Amato, L., Speranza, L. & Volpicelli, F. Neurotrophic factor BDNF, physiological functions and therapeutic potential in depression, neurodegeneration and brain cancer. *Int. J. Mol. Sci.* 10.3390/ijms21207777 (2020). PMC7589016 33096634

[45] Shvetcov, A. et al. The neuroprotective effects of estrogen and estrogenic compounds in spinal cord injury. *Neurosci. Biobehav. Rev.* **146**, 105074 (2023). 10.1016/j.neubiorev.2023.105074 36736846

[46] Fenchel, D. et al. Beyond the HPA-axis: the role of the gonadal steroid hormone receptors in modulating stress-related responses in an animal model of PTSD. *Eur. Neuropsychopharmacol.* **25**, 944–957 (2015). 10.1016/j.euroneuro.2015.02.004 25771251

[47] Koenigs, M. & Grafman, J. Posttraumatic stress disorder: the role of medial prefrontal cortex and amygdala. * Neuroscientist* **15**, 540–548 (2009). 10.1177/1073858409333072 PMC2771687 19359671

[48] Acheson, D. T., Gresack, J. E. & Risbrough, V. B. Hippocampal dysfunction effects on context memory: possible etiology for posttraumatic stress disorder. *Neuropharmacology* **62**, 674–685 (2012). 10.1016/j.neuropharm.2011.04.029 PMC3175276 21596050

[49] Hayes, J. P. et al. Reduced hippocampal and amygdala activity predicts memory distortions for trauma reminders in combat-related PTSD. *J. Psychiatr. Res.* **45**, 660–669 (2011). 10.1016/j.jpsychires.2010.10.007 PMC3081889 21047644

[50] Lee, S.-H., Zabolotny, J. M., Huang, H., Lee, H. & Kim, Y.-B. Insulin in the nervous system and the mind: functions in metabolism, memory, and mood. *Mol. Metab.* **5**, 589–601 (2016). 10.1016/j.molmet.2016.06.011 PMC5021669 27656397

[51] Ferrario, C. R. & Reagan, L. P. Insulin-mediated synaptic plasticity in the CNS: anatomical, functional and temporal contexts. *Neuropharmacology* **136**, 182–191 (2018). 10.1016/j.neuropharm.2017.12.001 PMC5988909 29217283

[52] Sharma, M., Yadav, Y. & Dey, C. S. Neuronal insulin signaling and resistance: a balancing act of kinases and phosphatases. *J. Endocrinol.* 10.1530/JOE-23-0151 (2023). 37930675

[53] Zhang, J., Kaye, A. P., Wang, J. & Girgenti, M. J. Transcriptomics of the depressed and PTSD brain. *Neurobiol. Stress* **15**, 100408 (2021). 10.1016/j.ynstr.2021.100408 PMC8524242 34703849

[54] He, Z., Qin, Y., Xie, G., Li, F. & Wu, N. Hippocampal transcriptome profiling associated with post-traumatic stress disorder-like behaviors in male mice. *Biochem. Biophys. Res. Commun.* **776**, 152205 (2025). 10.1016/j.bbrc.2025.152205 40540947

[55] Pilic, J. et al. Hexokinase 1 forms rings that constrict mitochondria during energy stress. *bioRxiv* 10.1101/2023.03.20.533440 (2023).

[56] Somvanshi, P. R. et al. Mechanistic inferences on metabolic dysfunction in posttraumatic stress disorder from an integrated model and multiomic analysis: role of glucocorticoid receptor sensitivity. *Am. J. Physiol. Endocrinol. Metab.* **317**, E879–e898 (2019). 10.1152/ajpendo.00065.2019 PMC6879860 31322414

[57] Summermatter, S. et al. PGC-1α improves glucose homeostasis in skeletal muscle in an activity-dependent manner. *Diabetes* **62**, 85–95 (2013). 10.2337/db12-0291 PMC3526021 23086035

[58] Krishnankutty, A. et al. In vivo regulation of glycogen synthase kinase 3β activity in neurons and brains. *Sci. Rep.* **7**, 8602 (2017). 10.1038/s41598-017-09239-5 PMC5561119 28819213

[59] Li, M. et al. Brain glycogen: a key to revealing the pathology of mental diseases. *Brain Res.* **1844**, 149194 (2024). 10.1016/j.brainres.2024.149194 39182899

[60] Muraleedharan, R. & Dasgupta, B. AMPK in the brain: its roles in glucose and neural metabolism. * FEBS J.* **289**, 2247–2262 (2022). 10.1111/febs.16151 34355526

