# Supplementary material for: Sex-dependent neurobiological and metabolic dysfunction in an aged rat model of post-traumatic stress disorder
Source: Commun Biol. 2026 Jun 19;9:1300. doi: 10.1038/s42003-026-10536-x (PMC13642903; doi:10.1038/s42003-026-10536-x)
Supplement: Supplementary file 1 [file 42003_2026_10536_MOESM1_ESM.pdf]

nature portfolio

Peer Review File

Sex-Dependent Neurobiological and Metabolic Dysfunction in an Aged Rat Model of Post-Traumatic Stress Disorder

Corresponding Author: Professor Peng Xiao

This file contains all reviewer reports in order by version, followed by all author rebuttals in order by version.

Version 0:

Reviewer comments:

Reviewer #1

(Remarks to the Author)
I really appreciate choosing me for a reviewer of ‘Sex Dependent Neurobiological and Metabolic Dysfunction in an Aged Rat Mod- 1 el of Post Traumatic Stress Disorder’. I have to admit that the topic and work included in the paper are relevant for journal’s scope. The manuscript addresses an important and timely topic, namely the neurobiological underpinnings of post-traumatic stress disorder (PTSD) in the elderly. By applying an aged animal model of PTSD induced by single prolonged stress combined with fear conditioning (SPS+FC), the authors aim to elucidate sex-specific metabolic and molecular alterations associated with PTSD pathogenesis. The study employs a multimodal approach integrating behavioral analyses, [^{18}F]FDG-PET imaging, plasma endocrine profiling, and brain transcriptomic analysis. Overall, the topic is of clear relevance to the field and addresses a significant gap in current PTSD research, particularly with respect to sex differences in aged individuals.
The major claims of the paper are, in principle, novel. To the best of my knowledge, this is the first study attempting to comprehensively characterize sex-specific metabolic and molecular brain alterations in an aged PTSD animal model using such an integrative methodological framework. As such, the manuscript is likely to be of interest to researchers working on PTSD, stress-related disorders. While the study may not immediately shift prevailing theoretical frameworks in the field, it does highlight several important and underexplored issues related to elderly PTSD and sex-dependent vulnerability, which could stimulate further research.
However, despite the strengths of the study design and its conceptual premise, the manuscript in its current form has substantial shortcomings that significantly weaken the strength and credibility of its claims. The manuscript gives the impression of having been written by multiple contributors without sufficient harmonization prior to submission. This lack of textual unification negatively affects the overall clarity and readability of the manuscript. Moreover, there are major problems in the presentation of the results and in the description and execution of the statistical analyses. These issues make the conclusions appear, at times, insufficiently supported by the data and somewhat overstated.
In particular, the Results section requires substantial revision. The structure is often unclear, key findings are not always presented in a transparent and systematic manner, and statistical details are frequently insufficient or ambiguous. This lack of clarity makes it difficult for the reader to properly evaluate the robustness of the reported effects. As the Conclusions and the Discussion should be directly built upon these results, the weaknesses in data presentation and statistical reporting propagate into interpretational overreach in several parts of the manuscript.
Importantly, I do not believe that additional experiments are strictly necessary to strengthen the manuscript. Rather, a thorough reworking of the Results section, clearer justification of the study aims, and a more rigorous and transparent description of the statistical analyses would substantially improve the overall quality and credibility of the work. At present, the unclear statistical descriptions contribute to an impression of overselling the findings, even if this may not reflect the authors’ actual intent.
The statistical analysis, as currently described, is not sufficiently detailed to allow full assessment of its soundness. This represents a major concern that must be addressed before the manuscript can be considered for publication. On a positive note, there are no apparent ethical concerns related to the use of animals in this study.
Examples of issues requiring clarification and improvement
Abstract
– Line 12: The phrase “pioneering study” appears somewhat overstated; a more cautious wording would be preferable.
Introduction
– Lines 73–81: This section does not substantially add to the Introduction and could be shortened or omitted.
Materials and Methods

– Line 116: Please clarify the meaning of the "$" symbol.
– Line 119: The term "constructed" does not seem optimal in this context and could be replaced with more appropriate wording.
– Lines 142–149: Please specify which software/system was used to evaluate animal behavior in the EPM and freezing behavior during fear conditioning. Overall, this section would benefit from additional methodological clarification.
The authors may consider adding a brief explanatory sentence clarifying the role of fear conditioning within the PTSD model and behavioral evaluation (e.g., that contextual fear conditioning was used to assess trauma-associated fear memory, while OFT and EPM assessed general anxiety-like behavior and locomotion).
– Lines 157–171: Please provide catalog numbers and manufacturers for the ELISA kits used. Additionally, the level of detail for the ELISA description appears disproportionate compared to other methods and could be streamlined.
– Lines 199–210: Information on brain tissue processing and homogenization prior to RNA extraction is missing and should be included.
– Lines 221–227: It is unclear whether this information is necessary in the Materials and Methods section and its relevance could be reconsidered.
– Lines 227–235: The statistical methods require clarification. It is not always clear whether the described analyses were consistently applied across datasets. In particular, clarification is needed regarding the use of two-way ANOVA in sex-specific hormonal analyses when data are presented for only one sex. Please also specify whether assumptions of normality and homogeneity of variance were tested.
Results
– Across multiple analyses, essential statistical details (e.g., F values, p values, main effects, and interactions) are missing. The Results section would benefit from a more comprehensive and transparent statistical description.
– Lines 243–245: Conclusions should not be based solely on graphs. Results corresponding to Fig. 1B–D and 1F–H should be explicitly described in the text, including non-significant findings.
– Line 252: The reported results are neither shown in the figure nor statistically described.
– Lines 254–257: Statistical details are missing and effects are not clearly represented in the figures.
– Line 275: Please clarify whether testosterone levels were measured in females; if not, provide a brief justification. It would also be helpful to clarify whether menopause was confirmed in aged females and whether estradiol levels were compared between sexes.
– Lines 284–285: The described effect is not clearly visible in the bar graphs; full statistical details should be provided.
– Lines 314–323: The described effects appear to be female-specific based on the figures. This should be clarified in the text and supported with appropriate figure references.
– Lines 319–320: This sentence appears more suitable for the Introduction or Discussion.
– Line 347: Please clarify whether the reported downward trend is statistically significant and provide the corresponding p value.
– Line 350: If correlation analyses were performed, please report the methods and outcomes.
– Lines 364–371: This section does not fit well within the Results and appears to repeat information from other sections; would be encourage to improve clarity.
– Line 373: If sex differences are claimed, the corresponding main effects or post hoc results should be reported; otherwise, the wording should be softened.
– Lines 406 and 424: References to the appropriate figures are missing, and some phrasing is not suitable for the Results section.
Figures
– Fig. 1: Please clarify the purpose of the rat cartoon and explain its meaning in the figure legend; its necessity could be reconsidered.
– Line 269: The information in brackets appears unnecessary in this context.
– Fig. 2: The abbreviation BDNF should be defined. Some functional descriptions of BDNF may not be directly relevant to peripheral measurements and could be omitted from the legend.
– Fig. 5: Please clarify abbreviations (e.g., BP, CC, MF) that are not shown directly in the figure.
– Fig. S1: The schematic is currently of limited informativeness. Adding more details (e.g., timing of stressors, age and sex of animals used, experimental timeline) would increase its value. Please also consider consistent terminology (e.g., "restraint" vs. "bondage").

– Additionally, the authors are encouraged to clarify why hippocampal and amygdala transcriptomic data were moved to the Supplementary section. Given that the behavioral paradigm primarily assesses contextual fear memory, hippocampal alterations may be central, while PFC and amygdala changes could be interpreted as modulatory rather than secondary or less important.
Discussion
– Line 427: The use of the term "aging" may be misleading when only a single age group is analysed; rephrasing is recommended.
– Lines 431–432: Please acknowledge the lack of changes in peripheral hormone levels, if applicable.
– Line 438: The data do not fully support direct conclusions regarding abnormal neuroplasticity; this claim should be moderated.
– Line 443: Phrases such as "stark contrast" could be replaced with more neutral scientific language.
– Line 452: The potential role of estradiol in aged females and its reduced neuroprotective effects after menopause should be discussed more cautiously.
– Lines 498–510: Statements regarding neuroinflammation appear speculative, as no direct measures were performed; this section should be toned down.
– Lines 511–523: The conclusion would benefit from a clearer summary of the main findings rather than focusing primarily on future research directions. Highlighting the limitations of the present study is also encouraged.

In conclusion, while the manuscript is not acceptable in its present form, it is based on a promising and potentially impactful study. I strongly encourage the authors to undertake a major revision focusing on improving the clarity and rigor of the Results section, the transparency of statistical analyses, and the depth of the Discussion. With these substantial revisions, the manuscript could make a valuable contribution to the field.

Reviewer #3

(Remarks to the Author)
The manuscript entitled Sex Dependent Neurobiological and Metabolic Dysfunction in an Aged Rat Model of Post Traumatic Stress Disorder by Hu et al. analysed a model of post-traumatic stress disorder based on neuroimaging, peripheral biomarkers, and molecular transcriptional characterizations in aged female and male rats.
The topic is interesting, although already examined in other publications; however, there are a number of concerns that do not allow a clear evaluation of the merit of the study.
In particular:
For which reason a number of genes are mentioned in the introduction since an open approach was adopted? They should be removed.
Methods: The overall number of animals should be mentioned. The reason why controls were singly housed should be detailed as well as which procedures were carried out on them.
I understand that the rats were acquired as 15 mo old and the stress carried out immediately after acclimatation, is this correct? And when was the pet scan carried out? Maybe supplementary figure S1 could be expanded to include timelines of all activities.
Details of the professional video tracking system analysis software are to be included.
Dimensions of the open field arena need specification.
Line 149 should be “entries”, not “time”.
Centrifugation speed should be reported in g, not rmp.
The method for ELISA assays is poorly described. Since manufacturer’s instructions are not available in English on the web, the authors are invited to describe the procedure in detail, in particular including the amount of plasma and dilution, how “the actual concentration of each analyte was calculated based on the established standard curve”, which curve fitting model was adopted. Moreover, testosterone conc. in the kit description on the web site of the provider is given in pg whereas manuscript results show nmol. In addition, no ELISA kit was available for BDNF on the web site.
The method for dissection of the three brain regions should be explained.
Which is “appropriate statistical software” mentioned at line 229?
The method for p value adjustment in transcriptomic analysis is either non-descript or missing. If the latter, the results are likely to contain a high number of false discoveries, therefore the value of the results is questionable.
Results: for all results, the statistical results in the two-way ANOVA tests for should be reported as F and degrees of freedom.
To evaluate EPM, OT% and OE% should be displayed and analysed.
Whereas the pathway enrichment is clearly described, the genes discussed as regulated appear to have been manually selected without a specified criterion.
Transcriptomic data should be made available on dedicated databases.
Discussion of available data from the literature about transcriptomics in PTSD rat models should be carried out.

Version 1:

Reviewer comments:

Reviewer #3

(Remarks to the Author)
The authors have satisfactorily addressed all concerns.

Open Access This Peer Review File is licensed under a Creative Commons Attribution 4.0 International License, which permits use, sharing, adaptation, distribution and reproduction in any medium or format, as long as you give appropriate credit to the original author(s) and the source, provide a link to the Creative Commons license, and indicate if changes were made.
In cases where reviewers are anonymous, credit should be given to 'Anonymous Referee' and the source.
The images or other third party material in this Peer Review File are included in the article's Creative Commons license, unless indicated otherwise in a credit line to the material. If material is not included in the article's Creative Commons license and your intended use is not permitted by statutory regulation or exceeds the permitted use, you will need to obtain permission directly from the copyright holder.
To view a copy of this license, visit https://creativecommons.org/licenses/by/4.0/

Response to Reviewers

Manuscript Title: Sex-Dependent Neurobiological and Metabolic Dysfunction in an Aged Rat Model of Post-Traumatic Stress Disorder

Manuscript ID: [COMMSBIO-25-11210-T]

Dear Editor and Reviewers,

We would like to express our sincere gratitude to the reviewers for their insightful comments and constructive suggestions. Their feedback has been instrumental in significantly strengthening the scientific rigor and clarity of our study.

Please note that in light of the comprehensive feedback, we have performed a substantial and systemic overhaul of the entire manuscript. These extensive modifications encompass the refinement of the experimental framework, the integration of multi-brain region datasets, and the standardization of our statistical reporting. Given the global nature of these revisions, the original narrative has been significantly reshaped to ensure a more cohesive and rigorous presentation. While specific changes are documented in the "Tracked Changes" version, we highly recommend that the reviewers refer to the final clean version of the manuscript for the most accurate and integrated representation of our revised work.

Reviewer #1 (Remarks to the Author):

General Response

We are deeply grateful for the reviewers' professional and constructive feedback, which has provided a vital roadmap for improving our manuscript. In light of these comments, we have meticulously revised the entire paper from the Introduction to the Conclusion. Notably, we have implemented major changes to our data analysis framework and transcriptomic interpretations, integrating detailed statistical parameters (F values, df, and P values) to ensure maximum transparency. We believe these comprehensive revisions have fundamentally strengthened the scientific narrative and the overall rigor of the study.

Abstract

Point 1: Line 12: The phrase 'pioneering study' appears somewhat overstated; a more cautious wording would be preferable.

Response: We agree. The word 'pioneering' has been removed to maintain a more cautious tone.

Changes: Line 12 now reads: 'This study systematically investigated...'

Introduction

Point 2: Lines 73–81: This section does not substantially add to the Introduction and could be shortened or omitted.

Response: We have significantly shortened this section, removing redundant gene-specific descriptions to focus on the broader metabolic and signaling framework.

Changes: The redundant descriptive section in the Introduction has been removed, resulting in a more focused and impactful opening narrative.

Materials and Methods

Point 3: Line 116: Please clarify the meaning of the '$' symbol.

Response: We sincerely apologize for this typographical error. The '$' symbol was an unintended character and has been removed in the revised manuscript. We have clarified that the sample size for each group is n = 6.

Changes: The erroneous symbol has been deleted, and the sample size is explicitly stated in Section 2.1 (Line 93).

Point 4: Line 119: The term 'constructed' does not seem optimal in this context and could be replaced with more appropriate wording.

Response: We have replaced 'constructed' with 'established.'

Changes: Line 104 now reads: 'a PTSD rat model was established...'

Point 5: Lines 142–149: Please specify which software/system was used to evaluate animal behavior in the EPM and freezing behavior during fear conditioning. Overall, this section would benefit from additional methodological clarification. The authors may consider adding a brief explanatory sentence clarify-

ing the role of fear conditioning within the PTSD model and behavioral evaluation (e.g., that contextual fear conditioning was used to assess trauma-associated fear memory, while OFT and EPM assessed general anxiety-like behavior and locomotion).

Response: We sincerely appreciate the reviewer's suggestion to enhance the methodological clarity of our behavioral assessments. We have updated the Materials and Methods section to provide a comprehensive description of the behavioral battery, including the specific software used and the functional rationale for each test.

To systematically assess the impact of trauma, all animal movements and freezing behaviors were recorded and analyzed using the DigiBehv professional video tracking system (Shanghai Jiliang Software Technology Co., Ltd., China). Furthermore, we have added a clarifying paragraph to define the distinct roles of each test within our PTSD model:

1. Contextual Fear Conditioning (CFC) was employed to specifically evaluate trauma-associated fear memory and freezing behavior (defined as the absence of all muscle movements except for respiration).

2. The Open Field Test (OFT) and Elevated Plus Maze (EPM) were utilized to assess general anxiety-like behavior and locomotor activity.

All behavioral tests were performed in a dedicated, soundproofed room under uniform lighting conditions to ensure environmental consistency. We have also included detailed parameters for each test, such as arena dimensions for the OFT and the calculation of OT% and OE% for the EPM, to ensure the reproducibility of our findings.

Changes: These methodological clarifications and functional definitions have been integrated into the revised Section 2.3 (Behavioral Assessments).

Point 6: Lines 157–171: Please provide catalog numbers and manufacturers for the ELISA kits used. Additionally, the level of detail for the ELISA description appears disproportionate compared to other methods and could be streamlined.

Response: In the revised manuscript, we have provided the full manufacturer information and specific catalog numbers for all ELISA kits used in this study. Furthermore, we have significantly streamlined the description of the ELISA procedure to focus on critical methodological parameters, such as sample volume, dilution ratios, and the mathematical model used for concentration calculation. These adjustments ensure that the level of detail is proportionate to other sections of the Materials and Methods.

Changes: The revised Section 2.4 (Endocrine Analysis) now includes the following details:

1. Manufacturer & Catalog Nos: Shanghai Affandi Industrial Co., Ltd., China (T, A109234; E2, A108219; CORT, A107689; BDNF, A107739).

2. Streamlined Procedure: We specified the 5-fold sample dilution (10 μL plasma in 40 μL diluent) and the use of a four-parameter logistic (4PL) regression model for data interpolation.

3. Units Consistency: The concentrations for T, E2, CORT, and BDNF are now explicitly expressed in nmol/L, ng/L, μg/mL, and ng/mL, respectively, ensuring clarity and adherence to the manufacturer's specifications.

Point 7: Lines 199–210: Information on brain tissue processing and homogenization prior to RNA extraction is missing and should be included.

Response: We have now provided a comprehensive description of the brain tissue processing and RNA extraction protocols in the revised manuscript. Specifically, we have added details regarding the anatomical landmarks used for dissection, the mechanical parameters for tissue homogenization, the specific chemical reagents employed, and the stringent quality control metrics used to verify RNA integrity. These additions ensure the reproducibility of our transcriptomic findings.

Changes: Detailed methodological information has been integrated into Section 2.6 (Tissue Processing and RNA Extraction). Key updates include:

Anatomical Precision: Specification that the PFC, Hipp, and Amyg were dissected on ice following the Paxinos and Watson rat brain atlas.

Homogenization Parameters: The use of a high-throughput homogenizer set at 60 Hz for 120 s with TRNzol Universal Reagent (TIANGEN, Cat. DP424).

Refined Extraction Protocol: Detailed centrifugation steps (reported in g) and the use of chloroform/isopropanol for phase separation and RNA precipitation.

Quality Control: Confirmation that all samples met strict integrity standards (RIN > 8.0 and 28S/18S > 1.8) using an Agilent 2100 Bioanalyzer prior to library construction.

Point 8: Lines 221–227: It is unclear whether this information is necessary in the Materials and Methods section and its relevance could be reconsidered.

Response: We agree with the reviewer that the previous description in this section lacked focus and contained redundant information. In response, we have completely restructured the Transcriptomics Analysis section (now Sections 2.7.1 and 2.7.2). We removed the unnecessary general descriptions and replaced them with a highly detailed, step-by-step bioinformatic pipeline.

The revised section now explicitly details the mRNA enrichment, library construction (Fast RNA-seq Lib Prep Kit V2), and the high-throughput sequencing platform (Illumina NovaSeq 6000). Crucially, we have clarified our systematic approach to gene selection: rather than manual picking, we constructed a targeted background gene set for glucose metabolism (retrieved from QuickGO and KEGG) and performed intersection analysis with identified DEGs. We also detailed the Benjamini-Hochberg (BH) procedure for FDR control and the PPI network clustering methods (MCL and K-means) used to isolate hub genes. These updates provide a robust and transparent methodological framework for our molecular findings.

Changes: The redundant information has been removed, and the comprehensive new protocol has been integrated into Section 2.7.1 (Library Preparation and Sequencing) and Section 2.7.2 (Bioinformatics and Differential Expression Analysis).

Point 9: Lines 227–235: The statistical methods require clarification. It is not always clear whether the described analyses were consistently applied across

datasets. In particular, clarification is needed regarding the use of two-way ANOVA in sex-specific hormonal analyses when data are presented for only one sex. Please also specify whether assumptions of normality and homogeneity of variance were tested.

Response: We have thoroughly revised the Statistical Analysis section to ensure clarity and consistency across all datasets.

Specifically, we have clarified the application of Two-way ANOVA (Sex × Group), which was exclusively used for datasets involving both sexes (Behavioral, PET, and shared biochemical data). For sex-restricted hormonal analyses (Testosterone and Estradiol), where data are intrinsically available for only one sex, we have now correctly applied and specified the use of unpaired Student's t-tests.

Furthermore, we have explicitly stated that the assumptions of normality (Shapiro-Wilk test) and homogeneity of variance (Levene's test) were rigorously verified prior to any parametric testing. For ANOVA results, we have now included all corresponding F values and degrees of freedom (*df*) to ensure full transparency, as suggested.

Changes: These methodological refinements have been integrated into the revised Section 2.8 (Statistical Analysis).

Results

Point 10: Across multiple analyses, essential statistical details (e.g., F values, p values, main effects, and interactions) are missing. The Results section would benefit from a more comprehensive and transparent statistical description.

Response: We entirely agree with the reviewer's assessment that statistical transparency is paramount for the validation of our findings. In the revised manuscript, we have performed a comprehensive overhaul of the Results section to ensure that every biological claim is supported by a complete set of statistical parameters.

Specifically, we have systematically integrated F values, degrees of freedom (*df*), p values, and, where applicable, the results of main effects and Sex × Group interactions for all Two-way ANOVA analyses. For post-hoc comparisons and t-tests, exact p

values are now reported to provide a clear measure of significance. We believe these additions significantly enhance the transparency and rigor of our data reporting.

Changes: The Results section (Sections 3.1 to 3.4) has been thoroughly updated with detailed statistical reporting.

Point 11: Lines 243–245:Conclusions should not be based solely on graphs. Results corresponding to Fig. 1B–D and 1F–H should be explicitly described in the text, including non-significant findings.

Response: We entirely agree with the reviewer that the Results section should provide a self-contained and transparent narrative. In the revised manuscript, we have added detailed textual descriptions for all data presented in the behavioral figures (now referenced as Figure 2 in the updated manuscript).

We have explicitly described both significant and non-significant findings, ensuring that the reader is informed of the statistical outcomes (F values, df, and P values) directly within the body of the text rather than relying solely on visual interpretations of the graphs. Specifically, for the EPM and OFT data (original Fig. 1B–D and 1F–H), we have detailed the main effects of Sex and Group, as well as their interaction, providing a more rigorous basis for our conclusions.

Changes: The Results section (specifically Section 3.1: Behavioral Alterations) has been expanded to include comprehensive descriptions of the behavioral phenotypes.

Point 12: Line 252: The reported results are neither shown in the figure nor statistically described.

Response: In the revised manuscript, we have ensured that all reported behavioral results are both visually represented and statistically supported.

Changes: We have updated the Results section (Section 3.1: Behavioral Alterations) to include explicit references to Figure 2A and the corresponding statistical parameters.

Point 13: Lines 254–257: Statistical details are missing and effects are not clearly represented in the figures.

Response: We have thoroughly revised this section to include all necessary statistical parameters and have ensured that the textual descriptions precisely align with the effects shown in the figures.

Changes: The statistical details for the fear conditioning test have been integrated into Section 3.1 (Behavioral Alterations), and the corresponding figure legends (Figure 2I-J) have been updated for clarity.

Point 14: Line 275: Please clarify whether testosterone levels were measured in females; if not, provide a brief justification. It would also be helpful to clarify whether menopause was confirmed in aged females and whether estradiol levels were compared between sexes.

Response: In the revised manuscript, we have provided a detailed justification for our hormone assay strategy and clarified the reproductive status of the aged female rats.

Hormone Selection & Cross-sex Comparisons: Testosterone (T) in females and estradiol (E2) in males were not measured because their concentrations are inherently negligible in 15-month-old aged rats. Consequently, cross-sex comparisons for these dominant steroids were not performed as they would not yield biologically meaningful results in this specific aged cohort.

Reproductive Status: The 15-month-old female rats used in this study represent a peri- or post-reproductive state, characterized by the senescence-associated cessation of regular estrous cycling typical of this age in Sprague-Dawley rats. This endocrine background is a critical component of our aged PTSD model.

Statistical Reporting: We have updated the Results section to include independent analysis of sex-dominant hormones. In males, T levels showed a significant elevation following trauma ($t_{(10)} = 2.384$, $P = 0.0383$), whereas in females, E2 levels remained statistically stable ($t_{(10)} = 0.3749$, $P = 0.7156$).

Changes: These clarifications and the corresponding statistical results (*t* values, *df*, and *P* values) have been integrated into Section 3.2 (Plasma Endocrine Profiling)

and are reflected in Figure 3B-C.

Point 15: Lines 284–285: The described effect is not clearly visible in the bar graphs; full statistical details should be provided.

Response: Upon re-evaluating the systemic BDNF data and the corresponding figures, we agree that the previously described 'increase' was statistically overstated. In the revised manuscript, we have corrected this narrative to reflect the statistical reality and have provided the full suite of statistical parameters as requested.

Our revised analysis confirms that while there was a slight numerical elevation in plasma BDNF levels in the PTSD groups (mean 62.08 ng/mL) compared to controls (mean 58.51 ng/mL), this difference did not reach statistical significance. We now explicitly state that peripheral BDNF levels remained statistically stable following the traumatic paradigm in both sexes. This corrected interpretation ensures that our conclusions are strictly grounded in the quantitative evidence.

Changes: The Results section (Section 3.2: Plasma Endocrine Profiling) has been updated to include full Two-way ANOVA details for BDNF.

Point 16: Lines 314–323: The described effects appear to be female-specific based on the figures. This should be clarified in the text and supported with appropriate figure references.

Response: In the revised manuscript, we have thoroughly updated the PET results section to distinguish between shared and sex-dependent metabolic patterns, supported by systematic figure references and Two-way ANOVA results.

Specifically, while we observed a general Group effect of metabolic suppression in the PFC and Hippocampus for both sexes, our quantitative analysis confirmed that the Amygdala (Amyg) hyperactivation is indeed a female-specific metabolic phenotype. Statistical analysis revealed a significant main effect of Sex in the Amyg ($F_{(1, 20)}$ = 16.38, P = 0.0006), and post-hoc comparisons explicitly showed significant hyperactivation only in female PTSD rats (P = 0.0151), whereas no significant change was detected in males (P = 0.1876). We have also relocated the interpretative sentences to

the Discussion section to maintain an objective Results narrative.

Changes: These clarifications, along with the detailed statistical parameters (*F* values, *df*, and *P* values), have been integrated into Section 3.3 (Cerebral Glucose Metabolic Alterations). We have also ensured that all claims are directly supported by references to Figure 4 (A–F) and the corresponding voxel-level data in Tables S2 and S3.

Point 17: Lines 319–320: This sentence appears more suitable for the Introduction or Discussion.

Response: We have removed the interpretative and background-related sentences from the Results section to ensure a more objective and concise presentation of our experimental findings. As suggested, these contents have been relocated to the Discussion section, where they now serve as a foundation for a more in-depth exploration of the neurobiological implications of our findings.

Changes: The corresponding sentences (previously in Lines 319–320) have been integrated into the revised Discussion (Section 4). In the updated narrative, we have expanded these points to discuss how the observed circuit-level metabolic alterations—specifically the divergence in amygdala activity—relate to broader neurobiological frameworks of sex-dependent stress vulnerability in the brain.

Point 18: Line 347: Please clarify whether the reported downward trend is statistically significant and provide the corresponding p value.

Response: In the revised manuscript, we have performed a comprehensive statistical analysis of the temporal dynamics and metabolic rates to clarify these trends.

Specifically, two-way repeated measures ANOVA for the Time-Activity Curves (TACs) identified a highly significant global effect of Group ($F_{(3,\ 180)} = 21.54$, $P < 0.0001$). For the quantified CMRglu, we observed a robust significant main effect of Group ($F_{(1,\ 20)} = 16.07$, $P = 0.0007$). Regarding the sex-specific post-hoc comparisons: while male PTSD rats exhibited a non-significant downward trend ($P = 0.3684$), the metabolic reduction in female PTSD rats was more pronounced and approached sta-

tistical significance (*P* = 0.0576). We have explicitly included these *F* values, degrees of freedom, and exact *p* values in the text to provide a transparent representation of the metabolic decline.

Changes: The Results section (Section 3.4 Cerebral Glucose Metabolic Alterations) and the legend for Figure 5 have been updated to include these detailed statistical parameters. The text now clearly distinguishes between the significant global group effect and the specific p-values for sex-based comparisons.

Point 19: Line 350: If correlation analyses were performed, please report the methods and outcomes.

Response: Correlation analyses are indeed crucial for linking our macroscopic metabolic findings to the observed behavioral phenotypes. In the revised manuscript, we have now explicitly detailed the methods and outcomes of these analyses.

We employed Pearson's correlation to evaluate the relationship between regional glucose metabolism (SUV) and the core PTSD-like symptom, namely freezing behavior during the contextual recall test. Our results revealed a robust functional dissociation: metabolic activity in the Hippocampus and PFC was negatively correlated with freezing time across both sexes, suggesting that higher executive and mnemonic metabolic integrity is associated with lower fear expression. Conversely, Amygdala metabolic activity showed a significant positive correlation with freezing behavior, reinforcing its role in fear potentiation. We have also included non-significant correlations (e.g., between whole-brain CMRglu and freezing) in the supplementary material to provide a balanced overview.

Changes: The methodology for correlation analysis has been added to Section 2.8 (Statistical Analysis), and the detailed results (*r* and *P* values) have been integrated into a new Section 3.5(Correlation of Regional Metabolism with Fear Memory). These findings are visually supported by the scatter plots in Figure 6 (A–C) and Figure S4.

Point 20: Lines 364–371: This section does not fit well within the Results and

appears to repeat information from other sections; would be encourage to improve clarity.

Response: The previously included section (Lines 364–371) primarily described the methodological framework for gene screening and metabolic background set construction, which was redundant as these details are already comprehensively covered in the Materials and Methods (Section 2.7.2).

To improve the clarity and narrative flow of the Results section, we have removed this entire repetitive segment. The updated text now transitions directly from the metabolic deficits observed in PET imaging to the specific transcriptomic alterations, ensuring that the Results section remains focused on our experimental findings rather than methodological justifications.

Changes: The redundant description has been deleted from the revised manuscript. The transition to the transcriptomic results (now in Section 3.6 has been streamlined to provide a more concise and logical presentation.

Point 21: Line 373: If sex differences are claimed, the corresponding main effects or post hoc results should be reported; otherwise, the wording should be softened.

Response: In response, we have overhauled the entire transcriptomic Results section (now Section 3.6) to provide a more rigorous and balanced narrative. Rather than making broad claims of "sex differences" without secondary interaction statistics, we have softened our wording to describe the "sex-specific regulatory patterns" and "spatial heterogeneity" observed across the PFC, Hipp, and Amyg.

Our revised reporting of the transcriptomic pipeline now encompasses:

1. Metabolic Targeting: We clarify how DEGs were intersected with a predefined glucose metabolism gene set to isolate functionally relevant candidates.

2. Functional Profiles: We describe the GO enrichment (BP, CC, and MF) across regions, noting that while many core metabolic processes are shared, their enrichment magnitude and specific gene constituents vary between sexes (e.g., more prominent metabolic signaling enrichment in the male PFC vs. the female Amygdala).

3. Network Architecture: We present the PPI network clustering (MCL and K-means), identifying specific hub genes (as shown in Fig. 8) that orchestrate these metabolic shifts in a region- and sex-dependent manner.

4. By shifting the focus to "comparative enrichment profiles" and "divergent molecular architectures," we believe the manuscript now more accurately represents the high-dimensional nature of the transcriptomic data.

Changes: Section 3.6 has been completely rewritten to reflect this comprehensive analysis. The terminology throughout this section has been softened from "significant sex differences" to "sex-specific patterns" and "region-dependent heterogeneity."

Point 22: Lines 406 and 424: References to the appropriate figures are missing, and some phrasing is not suitable for the Results section.

Response: In our comprehensive revision of the transcriptomics section (now Section 3.6), we have addressed these concerns by ensuring that every biological observation is directly linked to its corresponding visual data.

Changes: Section 3.6 has been systematically updated with precise figure citations. We have also refined the terminology to be more suitable for a Results section, focusing on "differential expression profiles," "network connectivity," and "clustering coefficients" rather than qualitative conclusions.

Figures

Point 23: Fig. 1: Please clarify the purpose of the rat cartoon and explain its meaning in the figure legend; its necessity could be reconsidered.

Response: To improve the clarity and professional presentation of our study design, we have removed the cartoon from Figure 1 as suggested. The revised figure now focuses strictly on the experimental timeline and the procedural workflow, ensuring that the visual emphasis remains on the scientific methodology.

Changes: The rat cartoon has been deleted from Figure 1 in the revised manuscript. The figure legend has also been updated to reflect the streamlined schematic, which now more clearly illustrates the 17-day experimental schedule.

Point 24: Line 269: The information in brackets appears unnecessary in this context.

Response: In the revised manuscript, we have thoroughly streamlined the legend for Figure 2 to provide a more concise and professional description of the behavioral data. All unnecessary bracketed information has been removed, and the terminology has been standardized across all behavioral metrics (e.g., OT%, OE%, and TIC).

Changes: The legend for Figure 2 has been updated as follows:

"Fig. 2. Sex-Specific Behavioral Phenotypes in Aged PTSD Rats. Behavioral assessments of aged male and female rats (n = 6 per group). (A) Representative movement trajectories in the EPM. (B-D) Quantification of (B) percentage of time spent in open arms (OT%), (C) percentage of open arm entries (OE%), and (D) closed arm entries (CAE) in the EPM. (E) Representative trajectories in the OFT. (F-H) Quantification of (F) total distance traveled (TD), (G) central area distance (CR), and (H) time spent in the center (TIC) in the OFT. (I-J) FC test results: (I) distance traveled during fear learning and (J) freezing time during fear memory recall. Data are presented as mean ± SEM. *P < 0.05*, **P* < 0.01*."

Point 25: Fig. 2: The abbreviation BDNF should be defined. Some functional descriptions of BDNF may not be directly relevant to peripheral measurements and could be omitted from the legend.

Response: In the revised manuscript, we have explicitly defined the abbreviation BDNF (Brain-Derived Neurotrophic Factor) upon its first mention in the figure legend. Furthermore, we have removed the peripheral-unrelated functional descriptions to ensure that the legend strictly describes the presented endocrine and biomarker data. We have also clarified the specific statistical tests (unpaired t-test vs. Two-way ANOVA) used for each panel to enhance methodological transparency.

Changes: The legend for Figure 3 (formerly Figure 2) has been streamlined as follows:

"Fig. 3. Systemic endocrine and neurotrophic factor profiles following traumatic stress. Plasma endocrine and biomarker profiles of rats (n = 6 per group). (A) Plasma

testosterone (T) levels in male rats. (B) Plasma estradiol (E2) levels in female rats. (C) Plasma corticosterone (CORT) levels across the four experimental groups. (D) Circulating brain-derived neurotrophic factor (BDNF) levels in both male and female rats. Data are presented as mean ± SEM. $P < 0.05$. Statistical significance was determined by unpaired Student's t-test (A, B) or Two-way ANOVA followed by Tukey's post-hoc test (C, D)."

Point 26: Fig. 5: Please clarify abbreviations (e.g., BP, CC, MF) that are not shown directly in the figure.

Response: In the revised manuscript, we have updated the legend for the GO enrichment analysis (now Figure 7) to explicitly define the abbreviations for the three main ontologies. Specifically, we have clarified that BP stands for Biological Process, CC for Cellular Component, and MF for Molecular Function.

Changes: The legend for Figure 7 has been revised as follows:

Fig. 7. GO functional enrichment analysis of genes related to different brain regions of PTSD model rats. (A-C) Enrichment results in the PFC (A), Hipp (B), and Amyg (C). The analysis incorporates differentially expressed genes from both male and female samples, categorized into Biological Process (BP), Cellular Component (CC), and Molecular Function (MF). The X-axis represents $-\log_{10}(\text{padj})$, where larger values indicate more significant enrichment.

Point 27: Fig. S1: The schematic is currently of limited informativeness. Adding more details (e.g., timing of stressors, age and sex of animals used, experimental timeline) would increase its value. Please also consider consistent terminology (e.g., 'restraint' vs. 'bondage')."

Response: We have completely redesigned this figure (now promoted to Figure 1 in the main manuscript) to serve as a comprehensive visual guide for our methodology.

The revised Figure 1 now incorporates the following critical details:

1. Experimental Timeline: A clear 17-day schedule, including adaptation, SPS+FS modeling, incubation, behavioral testing, and PET scanning phases.

2. Detailed Modeling Procedure: Specification of the Single Prolonged Stress plus Footshock (SPS+FS) protocol, including precise timings (e.g., 2h restraint, 20min swimming, 1mA 4s footshock).

3. Animal Characteristics: Explicit indication of the use of 15-month-old aged rats of both sexes (♂/♀).

4. Neuroimaging Protocols: Detailed breakdowns of both static and dynamic FDG-PET scanning sequences.

5. Standardized Terminology: As suggested, we have corrected the terminology, replacing informal terms like "bondage" with the standardized academic term "restraint stress.".

Changes: The previous supplementary schematic has been replaced by the updated Figure 1 in the main text. The figure legend has been expanded to provide a complete description of the multi-stage experimental workflow shown in panels A–D.

Point 28: Additionally, the authors are encouraged to clarify why hippocampal and amygdala transcriptomic data were moved to the Supplementary section. Given that the behavioral paradigm primarily assesses contextual fear memory, hippocampal alterations may be central, while PFC and amygdala changes could be interpreted as modulatory rather than secondary or less important.

Response: We fully agree that the Hippocampus (Hipp) and Amygdala (Amyg) are not secondary, but rather primary nodes in the neural circuitry governing contextual fear memory and its dysregulation in PTSD.

In the revised manuscript, we have re-integrated the complete transcriptomic datasets for both the Hippocampus and Amygdala into the main Results section (now detailed in Section 3.6). By presenting the molecular alterations of the PFC, Hipp, and Amyg side-by-side, we now offer a more holistic and integrated view of the brain-

wide metabolic and transcriptomic disruptions. This structural change allows us to better discuss how hippocampal deficits (central to memory) and amygdala hyperactivation (modulatory to fear expression) synergistically contribute to the sex-dependent phenotypes observed in our aged rat model.

Changes: The transcriptomic analysis of the Hippocampus and Amygdala has been moved from the Supplementary Material back to the main text.

Discussion

Point 29: Line 427: The use of the term 'aging' may be misleading when only a single age group is analysed; rephrasing is recommended."

Response: We agree with the reviewer that the term "aging" typically implies a longitudinal process or a comparison across multiple age cohorts, which was not the primary design of this study. To ensure scientific precision, we have revised our terminology throughout the manuscript, particularly in the concluding remarks.

We have replaced "aging" with more accurate descriptors such as "the aged brain" or "15-month-old aged rats" to clarify that our findings reflect the neurobiological state at a specific late-adult developmental stage. This rephrasing correctly frames our conclusions within the context of the established aged PTSD model rather than the biological process of aging itself.

Changes: The concluding sentence of the Results/Discussion transition (formerly Line 427) has been revised to:

"By integrating macro-scale neuroimaging with transcriptomics, this study offers a potential framework for understanding the neuroenergetic basis of sex-specific PTSD phenotypes in the aged brain. Our observations suggest that 15-month-old female rats may exhibit heightened behavioral vulnerability..."

Point 30: Lines 431–432: Please acknowledge the lack of changes in peripheral hormone levels, if applicable.

Response: In the revised Discussion, we have explicitly acknowledged and addressed the relative stability of certain peripheral hormone levels in the aged female

PTSD model, providing a biological context for these observations.

Specifically, we have clarified that while male PTSD rats exhibited significant elevations in plasma Corticosterone (CORT) and Testosterone (T), these fluctuations were notably absent in the female cohort. We interpret the stable CORT levels in 15-month-old female rats as a reflection of age-related alterations in HPA axis reactivity, where senescent females may maintain a baseline-like endocrine state despite traumatic stress. Furthermore, we have acknowledged that the stable but low levels of Estradiol (E2) (as shown in Fig. 3B-C) likely represent the exhaustion of classical neuroprotective buffers due to reproductive senescence. This lack of peripheral endocrine flexibility in females stands in contrast to the compensatory responses seen in males and underscores the unique bioenergetic vulnerability of the aged female brain.

Changes: The Discussion section (Section 4) has been expanded to include a dedicated analysis of these peripheral endocrine profiles. We have integrated references to our results (Figure 3) and relevant literature [43-46] to justify why this lack of hormonal change is a critical feature of the aged female stress response.

Point 31: Line 438: The data do not fully support direct conclusions regarding abnormal neuroplasticity; this claim should be moderated.

Response: We agree with the reviewer that our current datasets, while comprehensive in terms of neuroimaging and transcriptomics, do not provide direct structural or electrophysiological evidence of altered neuroplasticity. In accordance with this suggestion, we have removed the definitive claim regarding "abnormal neuroplasticity" to maintain a more rigorous and evidence-based narrative.

In the revised Discussion, we have re-framed this section to focus on the "potential for neuroanatomical and functional remodeling" as inferred from the significant dysregulation of metabolism-related genes and regional glucose utilization. By softening the language and characterizing these changes as "precursors to" or "indicators of" compromised synaptic integrity rather than confirmed plasticity deficits, we ensure that our conclusions remain strictly within the scope of the presented data.

Changes: The text in the Discussion (formerly Line 438) has been thoroughly

moderated.

Point 32: Line 443: Phrases such as 'stark contrast' could be replaced with more neutral scientific language."

Response: We agree that objective and neutral language is essential for a rigorous presentation of comparative data. In the revised manuscript, we have conducted a thorough linguistic review of the entire text to ensure a more balanced and professional tone.

Specifically, we have replaced subjective phrases such as "stark contrast" with more precise, neutral alternatives like "notable divergence," "significant difference," or "distinct patterns." In the context of our sex-dependent findings, we now describe the results by highlighting the specific directions and magnitudes of change in each cohort, allowing the statistical evidence to speak for itself without over-emphasizing the degree of difference through qualitative adjectives.

Changes: The phrasing in Line 443 (and throughout the Discussion) has been updated. For instance, the comparison between male and female metabolic responses now uses more formal academic structures, such as "In contrast to the patterns observed in males, female rats exhibited..." or "A significant divergence was noted between..."

Point 33: Line 452: The potential role of estradiol in aged females and its reduced neuroprotective effects after menopause should be discussed more cautiously.

Response: We agree that the neuroprotective efficacy of estradiol (E2) is highly dependent on the reproductive stage and the functional state of estrogen receptors.

In the revised Discussion, we have moderated our claims to reflect the biological reality of reproductive senescence. We now explicitly discuss that while E2 is conventionally neuroprotective in younger cohorts, this efficacy appears to diminish significantly in our 15-month-old female model. By highlighting our observation of stable but low serum E2 levels (Fig. 3C), we suggest that the aged female brain may have

passed the "window of opportunity" for classical neuroprotection, leaving it more vulnerable to traumatic injury. This refined narrative avoids overstating the protective role of residual E2 and instead focuses on the age-related attenuation of endocrine buffering, which aligns with the observed metabolic and behavioral deficits.

Changes: The Discussion section (Section 4) has been updated with a more nuanced analysis of the E2 data.

Point 34: Lines 498–510: Statements regarding neuroinflammation appear speculative, as no direct measures were performed; this section should be toned down.

Response: We fully agree with the reviewer's assessment that without direct histological or biochemical markers of neuroinflammation (such as Iba1 or cytokine assays), conclusions in this area remain speculative. Consequently, we have removed all speculative statements regarding neuroinflammation from the Discussion.

In the revised manuscript, we have refocused this section (Lines 498–510 in the original, now expanded in Section 4) on the mechanistic interpretation of our transcriptomic and PET imaging data, for which we have direct evidence. We now provide a detailed analysis of the central insulin and AMPK signaling networks. Specifically, we discuss how the coordinated dysregulation of key metabolic sensors and enzymes (e.g., *Gsk3b*, *Hk1*, *Ppargc1a*, and *Prkaa2*) reflects a state of "metabolic emergency" and mitochondrial strain. By framing our findings within the context of bioenergetic reprogramming and metabolic signaling "tuning," we offer a more grounded and evidence-based explanation for the observed sex-dependent PTSD phenotypes.

Changes: The section discussing neuroinflammation has been deleted. It has been replaced with a rigorous discussion of the Insulin-AMPK axis as a central metabolic-behavioral nexus. This revised narrative integrates our findings on rate-limiting glycolytic enzymes and energy sensors to explain the potential molecular substrates of the observed metabolic deficits, supported by relevant literature [50–60].

Point 35: Lines 511–523: The conclusion would benefit from a clearer sum-

mary of the main findings rather than focusing primarily on future research directions. Highlighting the limitations of the present study is also encouraged.

Response: In the revised manuscript, we have completely restructured the concluding paragraph to provide a concise synthesis of our primary findings while maintaining an objective perspective on the study's constraints.

The revised conclusion now clearly highlights:

1. Core Findings: The demonstration of sex-dependent PTSD-like phenotypes in 15-month-old rats, characterized by a unique decoupling from peripheral hormonal status.
2. Multimodal Integration: The alignment between macroscopic metabolic suppression (via FDG-PET) and molecular-level reprogramming of the central Insulin-AMPK signaling axis (specifically involving Gsk3b, Hk1, and Prkaa2).
3. Study Limitations: We have added a dedicated discussion on the inherent limitations of our study, including the use of bulk tissue transcriptomics (which lacks cell-type resolution), the cross-sectional design (precluding longitudinal tracking), and the need for protein-level functional validation.
4. By acknowledging these limitations and focusing on the identified neuroenergetic framework, we believe the conclusion now provides a more balanced and rigorous final statement.

Changes: The Conclusion section (Lines 511–523 in the original, now updated at the end of Section 4) has been rewritten to reflect this comprehensive summary.

Response to Reviewers

Manuscript Title: Sex-Dependent Neurobiological and Metabolic Dysfunction in an Aged Rat Model of Post-Traumatic Stress Disorder

Manuscript ID: [COMMSBIO-25-11210-T]

Dear Editor and Reviewers,

We would like to express our sincere gratitude to the reviewers for their insightful comments and constructive suggestions, which have significantly improved the quality and rigor of our manuscript. We have carefully addressed each point raised by the reviewers and revised the manuscript accordingly.

Reviewer #2 (Remarks to the Author):

Introduction

Point 1: For which reason a number of genes mentioned in the introduction since an open approach was adopted? They should be removed.

Response: We completely agree with the reviewer's observation. Since this study employs an unbiased, open-ended transcriptomic approach to identify molecular signatures of metabolic dysfunction, mentioning specific candidate genes in the Introduction was premature and logically inconsistent with an exploratory discovery pipeline.

To rectify this, we have removed all references to specific genes (such as *Gsk3b*, *Hk1*, etc.) from the Introduction. The revised Introduction now focuses on the broader biological significance of neuroenergetic failure in PTSD and the rationale for using a multi-omics approach to bridge the gap between macroscopic imaging and microscopic molecular shifts. This adjustment ensures that our study's "discovery-based" nature is accurately reflected in the narrative flow.

Changes: The Introduction has been streamlined to focus on the research gaps in aged PTSD models and metabolic signaling, without pre-specifying gene targets. All specific gene names previously mentioned have been deleted to maintain the integrity of the open-approach framework.

Materials and Methods

Point 2 & 3: Methods: The overall number of animals should be mentioned.The reason why controls were singly housed should be detailed as well as which procedures were carried out on them."

Response: We thank the reviewer for these important methodological queries. We have now provided comprehensive details in the revised Materials and Methods (Section 2.1) to ensure full transparency regarding animal welfare and experimental control.

Changes: The "Animals " section has been expanded to include the total count, the justification for single housing, and the specific handling protocols for the control cohorts.

Point 4 & 5: I understand that the rats were acquired as 15 mo old and the stress carried out immediately after acclimatation, is this correct?"And when was the pet scan carried out? Maybe supplementary figure S1 could be expanded to include timelines of all activities."

Response: The reviewer's understanding is correct. We utilized 15-month-old aged rats, and the stress induction (SPS+FS protocol) was initiated on Day 8, immediately following a 7-day acclimatization period. To provide a clear and comprehensive overview of all experimental activities, we have completely redesigned the schematic diagram. This figure has been promoted from the supplementary material to the main text as Figure 1.

As illustrated in the expanded Figure 1A, the experimental sequence was as follows:

1. Days 1–7 (Adaptation): Following acquisition at 15 months of age, all rats underwent 7 days of environment and handling acclimatization.
2. Day 8 (SPS+FS Modeling): The stress protocol was performed.
3. Days 9–15 (Incubation): A 7-day post-stress incubation period to allow for the development of PTSD-like phenotypes.
4. Day 16 (Behavioral Assessment): EPM, OFT, and Fear Conditioning tests were conducted.

5. Day 17 (PET Scanning): FDG-PET neuroimaging (including both static and dynamic protocols, as detailed in Figure 1D) was performed on the final experimental day.

Changes: We have replaced the previous supplementary schematic with a much more detailed and informative **Figure 1** in the main manuscript. This new figure integrates the timelines for modeling, behavioral testing, and neuroimaging assessments into a single, intuitive framework.

Point 6 & 7: Details of the professional video tracking system analysis software are to be included. Dimensions of the open field arena need specification.

Response: In the revised Materials and Methods (Sections 2.3), we have now included the specific details of the video tracking system and the precise dimensions of all behavioral apparatuses to ensure full methodological transparency.

Specifically, the following information has been added:

1. Video Tracking Software: All animal movements, trajectories, and freezing behaviors were recorded and quantified using the DigiBehv professional video tracking system (Shanghai Jiliang Software Technology Co., Ltd., China). This system allowed for automated, objective analysis of key metrics such as distance traveled, zone-specific residence time, and freezing duration.

2. OFT Dimensions: The Open Field Test was conducted in a white, square arena with dimensions of 100 × 100 × 50 cm.

3. EPM and Fear Box Details: We have also specified that the EPM was elevated 50 cm above the floor and detailed the definition of freezing behavior used by the software (absence of all movement except for respiration) to quantify contextual fear memory.

Changes: The behavioral assessment sections have been expanded to include the manufacturer details of the tracking software and the exact physical dimensions of the OFT and EPM apparatuses.

Point 8: Line 149 should be 'entries', not 'time'.

Response: We have corrected the terminology in the revised Materials and Methods (Section 2.3) The previous wording incorrectly used "time" in a context where "entries" was the appropriate metric. In the updated manuscript, we have replaced "time" with "entries" to accurately reflect the quantification of animal movements into the maze arms.

The parameters analyzed now clearly distinguish between the number of open arm entries, time spent in open arms, and closed arm entries. These raw metrics were subsequently used to calculate the percentage of open arm entries (OE%) and the percentage of time spent in open arms (OT%) as standardized measures of anxiety-like behavior.

Changes: The description of EPM parameters has been corrected. The revised sentence now reads: Parameters analyzed included the number of open arm entries, time spent in open arms, and closed arm entries."

Point 9: Centrifugation speed should be reported in g, not rmp.

Response: We agree that reporting centrifugation intensity in relative centrifugal force (g) is essential for the reproducibility of the protocol. In the revised manuscript, we have added a dedicated section (Section 2.6 Tissue Processing and RNA Extraction) and updated all centrifugation parameters to g.

Changes: A new subsection on tissue processing and RNA extraction has been integrated into the Materials and Methods. All references to "rpm" have been removed and replaced with the corresponding "g" values to comply with standard laboratory reporting practices.

Point 10: The method for ELISA assays is poorly described. Since manufacturer's instructions are not available in English on the web, the authors are invited to describe the procedure in detail, in particular including the amount of plasma and dilution, how 'the actual concentration of each analyte was calculated based on the established standard curve', which curve fitting model was adopted.

Response: We recognize that international reproducibility depends on clear procedural descriptions. In the revised Materials and Methods (Section 2.2), we have now fully detailed the ELISA protocols for T, E2, CORT, and BDNF.

Key technical details added to the manuscript include:

1. Sampling and Dilution: 10 μL of plasma sample was added to 40 μL of sample diluent, achieving a 5-fold dilution for each assay.

2. Standard Curve Fitting: To ensure the highest accuracy in quantifying analyte concentrations, we utilized a four-parameter logistic (4PL) regression model to generate the standard curves. This non-linear model is the gold standard for ELISA data as it accounts for the sigmoidal relationship between optical density (OD) and concentration.

3. Measurement Parameters: We have included the detection wavelength (450 nm) and the specific Catalog Numbers for the kits from Shanghai Affandi Industrial Co., Ltd. (T: A109234; E2: A108219; CORT: A107689; BDNF: A107739).

4. Temporal Control: We also clarified that blood collection was restricted to a specific time window (09:00–11:00) to control for circadian variations in hormone levels.

Changes: The "Endocrine and Biomarker Analysis" section has been significantly expanded to incorporate these procedural details, ensuring that the methodology is transparent and reproducible despite the limited online availability of the original manufacturer's manual in English.

Point 11: The method for dissection of the three brain regions should be explained.

Response: Precise anatomical dissection is indeed critical for the validity of our region-specific transcriptomic findings. In the revised Materials and Methods (Section 2.6) we have explicitly described the dissection procedure.

Changes: Section 2.6 has been updated to include the anatomical reference used for dissection and the specific thermal conditions maintained during tissue collection.

Point 12: Which is 'appropriate statistical software' mentioned at line 229?

Response: In the revised manuscript, we have replaced the vague term "appropriate statistical software" with the specific versions of the platforms employed for our analyses. All primary statistical analyses and graphing were performed using GraphPad Prism (v9.0) and R (v4.2.1).

Changes: Section 2.8 has been rewritten to explicitly name the software used and detail the comprehensive statistical workflow, ensuring the rigor and reproducibility of our data interpretation.

Point 13: The method for p value adjustment in transcriptomic analysis is either non-descript or missing. If the latter, the results are likely to contain a high number of false discoveries, therefore the value of the results is questionable.

Response: We fully acknowledge the reviewer's concern regarding the risk of false discoveries in high-throughput transcriptomic data. Rigorous statistical correction is indeed essential for the validity of differential expression analysis. In the revised manuscript, we have now explicitly detailed our bioinformatics pipeline and the specific methods used to control the False Discovery Rate (FDR).

Changes: Section 2.7.2 has been expanded to include the specific bioinformatics tools (fastp, HISAT2, FeatureCounts, and DESeq2) and the exact parameters used for *P* value adjustment and DEG filtering.

Results

Point 14 & 15: Results: for all results, the statistical results in the two-way ANOVA tests for should be reported as F and degrees of freedom.To evaluate EPM, OT% and OE% should be displayed and analysed.

Response: We fully agree with the reviewer that reporting the complete statistical parameters (*F* values and degrees of freedom) is essential for a transparent and rigorous interpretation of the data. In the revised manuscript, we have updated all behavioral, PET, and biochemical results to include these values.

As requested, we have also refined our analysis of the Elevated Plus Maze (EPM)

by focusing on the standard anxiety indicators: the percentage of time spent in open arms (OT%) and the percentage of open arm entries (OE%).

Changes: Figures 2B and 2C have been updated to display OT% and OE%, respectively. The corresponding Results text and figure legends now include the full *F* values, *df*, and *P* values for all Two-way ANOVA and post-hoc comparisons.

Point 16: Whereas the pathway enrichment is clearly described, the genes discussed as regulated appear to have been manually selected without a specified criterion.

Response: We agree that a transparent, criteria-based pipeline is essential to avoid selection bias. In the revised manuscript, we have now explicitly defined the three-stage hierarchical filtering process used to identify the key genes for discussion. As detailed in Section 2.7.2.

Changes: Section 2.7.2 has been rewritten to formally state these selection criteria. We believe this systematic approach ensures that the discussed genes represent the most statistically robust and functionally central regulators of the observed metabolic dysfunction.

Data Availability

Point 17: Transcriptomic data should be made available on dedicated databases.

Response: We fully agree with the importance of data accessibility and transparency in genomic research. In accordance with the reviewer's recommendation, the raw and processed transcriptomic datasets generated in this study have been deposited in the Gene Expression Omnibus (GEO) database.

The record is currently in private status to facilitate anonymous peer review, with a scheduled public release date of **April 5, 2027**. During the review process, the data can be accessed by editors and reviewers using the following secure token:

- GEO Accession Number: GSE327211

- Reviewer Access Token: qdetumeavhytduh
- Access Link: https://www.ncbi.nlm.nih.gov/geo/query/acc.cgi?acc=GSE327211

To review the record, please navigate to the link above and enter the provided token into the required field. This ensures that our molecular findings are fully traceable and verifiable.

Changes: A **Data Availability Statement** has been added to the manuscript, specifying the GEO accession number and confirming that all transcriptomic data will be publicly available upon publication or by the specified release date.

Discussion

Point 18: Discussion of available data from the literature about transcriptomics in PTSD rat models should be carried out.

Response: In the revised Discussion (Section 4), we have integrated a comparative analysis between our results and existing literature on rodent PTSD models.

Specifically, we have discussed how our findings align with and expand upon current knowledge:

1. Conserved Pathways: We acknowledge that multi-species transcriptomic meta-analyses and hippocampal-specific profiling in other PTSD models have consistently identified disruptions in bioenergetic and synaptic pathways [53, 54].
2. Unique Contribution of the Aged Model: While previous studies have laid the groundwork, our data in 15-month-old rats uniquely underscore a systemic reprogramming of insulin-AMPK metabolic signaling cascades.
3. Mechanistic Alignment: We discuss our observed dysregulation of the Insulin-AMPK axis (e.g., Gsk3b, Hk1, Prkaa2) in the context of "metabolic emergency" and mitochondrial insufficiency. This aligns with emerging evidence that PTSD is characterized by a fundamental failure in neuroenergetic regulation, but adds a novel layer regarding the sexual dimorphism of these shifts in the aged brain.

Changes: The Discussion section has been expanded to include references to key transcriptomic studies [53-60], establishing a clearer link between our region-

specific results and the established molecular signatures of PTSD in the literature.

Response to Reviewers

Manuscript Title: Sex-Dependent Neurobiological and Metabolic Dysfunction in an Aged Rat Model of Post-Traumatic Stress Disorder

Manuscript ID: [COMMSBIO-25-11210-T]

Dear Editor and Reviewers,

We would like to express our sincere gratitude to the reviewers for their insightful comments and constructive suggestions. Their feedback has been instrumental in significantly strengthening the scientific rigor and clarity of our study.

Please note that in light of the comprehensive feedback, we have performed a substantial and systemic overhaul of the entire manuscript. These extensive modifications encompass the refinement of the experimental framework, the integration of multi-brain region datasets, and the standardization of our statistical reporting. Given the global nature of these revisions, the original narrative has been significantly reshaped to ensure a more cohesive and rigorous presentation. While specific changes are documented in the "Tracked Changes" version, we highly recommend that the reviewers refer to the final clean version of the manuscript for the most accurate and integrated representation of our revised work.

Reviewer #1 (Remarks to the Author):

General Response

We are deeply grateful for the reviewers' professional and constructive feedback, which has provided a vital roadmap for improving our manuscript. In light of these comments, we have meticulously revised the entire paper from the Introduction to the Conclusion. Notably, we have implemented major changes to our data analysis framework and transcriptomic interpretations, integrating detailed statistical parameters (*F* values, *df*, and *P* values) to ensure maximum transparency. We believe these comprehensive revisions have fundamentally strengthened the scientific narrative and the overall rigor of the study.

Abstract

Point 1: Line 12: The phrase 'pioneering study' appears somewhat overstated; a more cautious wording would be preferable.

Response: We agree. The word 'pioneering' has been removed to maintain a more cautious tone.

Changes: Line 12 now reads: 'This study systematically investigated...'

Introduction

Point 2: Lines 73–81: This section does not substantially add to the Introduction and could be shortened or omitted.

Response: We have significantly shortened this section, removing redundant gene-specific descriptions to focus on the broader metabolic and signaling framework.

Changes: The redundant descriptive section in the Introduction has been removed, resulting in a more focused and impactful opening narrative.

Materials and Methods

Point 3: Line 116: Please clarify the meaning of the '$' symbol.

Response: We sincerely apologize for this typographical error. The '$' symbol was an unintended character and has been removed in the revised manuscript. We have clarified that the sample size for each group is n = 6.

Changes: The erroneous symbol has been deleted, and the sample size is explicitly stated in Section 2.1 (Line 93).

Point 4: Line 119: The term 'constructed' does not seem optimal in this context and could be replaced with more appropriate wording.

Response: We have replaced 'constructed' with 'established.'

Changes: Line 104 now reads: 'a PTSD rat model was established...'

Point 5: Lines 142–149: Please specify which software/system was used to evaluate animal behavior in the EPM and freezing behavior during fear conditioning. Overall, this section would benefit from additional methodological clarification. The authors may consider adding a brief explanatory sentence clarify-

ing the role of fear conditioning within the PTSD model and behavioral evaluation (e.g., that contextual fear conditioning was used to assess trauma-associated fear memory, while OFT and EPM assessed general anxiety-like behavior and locomotion).

Response: We sincerely appreciate the reviewer's suggestion to enhance the methodological clarity of our behavioral assessments. We have updated the Materials and Methods section to provide a comprehensive description of the behavioral battery, including the specific software used and the functional rationale for each test.

To systematically assess the impact of trauma, all animal movements and freezing behaviors were recorded and analyzed using the DigiBehv professional video tracking system (Shanghai Jiliang Software Technology Co., Ltd., China). Furthermore, we have added a clarifying paragraph to define the distinct roles of each test within our PTSD model:

1. Contextual Fear Conditioning (CFC) was employed to specifically evaluate trauma-associated fear memory and freezing behavior (defined as the absence of all muscle movements except for respiration).

2. The Open Field Test (OFT) and Elevated Plus Maze (EPM) were utilized to assess general anxiety-like behavior and locomotor activity.

All behavioral tests were performed in a dedicated, soundproofed room under uniform lighting conditions to ensure environmental consistency. We have also included detailed parameters for each test, such as arena dimensions for the OFT and the calculation of OT% and OE% for the EPM, to ensure the reproducibility of our findings.

Changes: These methodological clarifications and functional definitions have been integrated into the revised Section 2.3 (Behavioral Assessments).

Point 6: Lines 157–171: Please provide catalog numbers and manufacturers for the ELISA kits used. Additionally, the level of detail for the ELISA description appears disproportionate compared to other methods and could be streamlined.

Response: In the revised manuscript, we have provided the full manufacturer information and specific catalog numbers for all ELISA kits used in this study. Furthermore, we have significantly streamlined the description of the ELISA procedure to focus on critical methodological parameters, such as sample volume, dilution ratios, and the mathematical model used for concentration calculation. These adjustments ensure that the level of detail is proportionate to other sections of the Materials and Methods.

Changes: The revised Section 2.4 (Endocrine Analysis) now includes the following details:

1. Manufacturer & Catalog Nos: Shanghai Affandi Industrial Co., Ltd., China (T, A109234; E2, A108219; CORT, A107689; BDNF, A107739).

2. Streamlined Procedure: We specified the 5-fold sample dilution (10 μL plasma in 40 μL diluent) and the use of a four-parameter logistic (4PL) regression model for data interpolation.

3. Units Consistency: The concentrations for T, E2, CORT, and BDNF are now explicitly expressed in nmol/L, ng/L, μg/mL, and ng/mL, respectively, ensuring clarity and adherence to the manufacturer's specifications.

Point 7: Lines 199–210: Information on brain tissue processing and homogenization prior to RNA extraction is missing and should be included.

Response: We have now provided a comprehensive description of the brain tissue processing and RNA extraction protocols in the revised manuscript. Specifically, we have added details regarding the anatomical landmarks used for dissection, the mechanical parameters for tissue homogenization, the specific chemical reagents employed, and the stringent quality control metrics used to verify RNA integrity. These additions ensure the reproducibility of our transcriptomic findings.

Changes: Detailed methodological information has been integrated into Section 2.6 (Tissue Processing and RNA Extraction). Key updates include:

Anatomical Precision: Specification that the PFC, Hipp, and Amyg were dissected on ice following the Paxinos and Watson rat brain atlas.

Homogenization Parameters: The use of a high-throughput homogenizer set at 60 Hz for 120 s with TRNzol Universal Reagent (TIANGEN, Cat. DP424).

Refined Extraction Protocol: Detailed centrifugation steps (reported in g) and the use of chloroform/isopropanol for phase separation and RNA precipitation.

Quality Control: Confirmation that all samples met strict integrity standards (RIN > 8.0 and 28S/18S > 1.8) using an Agilent 2100 Bioanalyzer prior to library construction.

Point 8: Lines 221–227: It is unclear whether this information is necessary in the Materials and Methods section and its relevance could be reconsidered.

Response: We agree with the reviewer that the previous description in this section lacked focus and contained redundant information. In response, we have completely restructured the Transcriptomics Analysis section (now Sections 2.7.1 and 2.7.2). We removed the unnecessary general descriptions and replaced them with a highly detailed, step-by-step bioinformatic pipeline.

The revised section now explicitly details the mRNA enrichment, library construction (Fast RNA-seq Lib Prep Kit V2), and the high-throughput sequencing platform (Illumina NovaSeq 6000). Crucially, we have clarified our systematic approach to gene selection: rather than manual picking, we constructed a targeted background gene set for glucose metabolism (retrieved from QuickGO and KEGG) and performed intersection analysis with identified DEGs. We also detailed the Benjamini-Hochberg (BH) procedure for FDR control and the PPI network clustering methods (MCL and K-means) used to isolate hub genes. These updates provide a robust and transparent methodological framework for our molecular findings.

Changes: The redundant information has been removed, and the comprehensive new protocol has been integrated into Section 2.7.1 (Library Preparation and Sequencing) and Section 2.7.2 (Bioinformatics and Differential Expression Analysis).

Point 9: Lines 227–235: The statistical methods require clarification. It is not always clear whether the described analyses were consistently applied across

datasets. In particular, clarification is needed regarding the use of two-way ANOVA in sex-specific hormonal analyses when data are presented for only one sex. Please also specify whether assumptions of normality and homogeneity of variance were tested.

Response: We have thoroughly revised the Statistical Analysis section to ensure clarity and consistency across all datasets.

Specifically, we have clarified the application of Two-way ANOVA (Sex × Group), which was exclusively used for datasets involving both sexes (Behavioral, PET, and shared biochemical data). For sex-restricted hormonal analyses (Testosterone and Estradiol), where data are intrinsically available for only one sex, we have now correctly applied and specified the use of unpaired Student's t-tests.

Furthermore, we have explicitly stated that the assumptions of normality (Shapiro-Wilk test) and homogeneity of variance (Levene's test) were rigorously verified prior to any parametric testing. For ANOVA results, we have now included all corresponding F values and degrees of freedom (df) to ensure full transparency, as suggested.

Changes: These methodological refinements have been integrated into the revised Section 2.8 (Statistical Analysis).

Results

Point 10: Across multiple analyses, essential statistical details (e.g., F values, p values, main effects, and interactions) are missing. The Results section would benefit from a more comprehensive and transparent statistical description.

Response: We entirely agree with the reviewer's assessment that statistical transparency is paramount for the validation of our findings. In the revised manuscript, we have performed a comprehensive overhaul of the Results section to ensure that every biological claim is supported by a complete set of statistical parameters.

Specifically, we have systematically integrated F values, degrees of freedom (df), p values, and, where applicable, the results of main effects and Sex × Group interactions for all Two-way ANOVA analyses. For post-hoc comparisons and t-tests, exact p

values are now reported to provide a clear measure of significance. We believe these additions significantly enhance the transparency and rigor of our data reporting.

Changes: The Results section (Sections 3.1 to 3.4) has been thoroughly updated with detailed statistical reporting.

Point 11: Lines 243–245:Conclusions should not be based solely on graphs. Results corresponding to Fig. 1B–D and 1F–H should be explicitly described in the text, including non-significant findings.

Response: We entirely agree with the reviewer that the Results section should provide a self-contained and transparent narrative. In the revised manuscript, we have added detailed textual descriptions for all data presented in the behavioral figures (now referenced as Figure 2 in the updated manuscript).

We have explicitly described both significant and non-significant findings, ensuring that the reader is informed of the statistical outcomes (F values, df, and P values) directly within the body of the text rather than relying solely on visual interpretations of the graphs. Specifically, for the EPM and OFT data (original Fig. 1B–D and 1F–H), we have detailed the main effects of Sex and Group, as well as their interaction, providing a more rigorous basis for our conclusions.

Changes: The Results section (specifically Section 3.1: Behavioral Alterations) has been expanded to include comprehensive descriptions of the behavioral phenotypes.

Point 12: Line 252: The reported results are neither shown in the figure nor statistically described.

Response: In the revised manuscript, we have ensured that all reported behavioral results are both visually represented and statistically supported.

Changes: We have updated the Results section (Section 3.1: Behavioral Alterations) to include explicit references to Figure 2A and the corresponding statistical parameters.

Point 13: Lines 254–257: Statistical details are missing and effects are not clearly represented in the figures.

Response: We have thoroughly revised this section to include all necessary statistical parameters and have ensured that the textual descriptions precisely align with the effects shown in the figures.

Changes: The statistical details for the fear conditioning test have been integrated into Section 3.1 (Behavioral Alterations), and the corresponding figure legends (Figure 2I-J) have been updated for clarity.

Point 14: Line 275: Please clarify whether testosterone levels were measured in females; if not, provide a brief justification. It would also be helpful to clarify whether menopause was confirmed in aged females and whether estradiol levels were compared between sexes.

Response: In the revised manuscript, we have provided a detailed justification for our hormone assay strategy and clarified the reproductive status of the aged female rats.

Hormone Selection & Cross-sex Comparisons: Testosterone (T) in females and estradiol (E2) in males were not measured because their concentrations are inherently negligible in 15-month-old aged rats. Consequently, cross-sex comparisons for these dominant steroids were not performed as they would not yield biologically meaningful results in this specific aged cohort.

Reproductive Status: The 15-month-old female rats used in this study represent a peri- or post-reproductive state, characterized by the senescence-associated cessation of regular estrous cycling typical of this age in Sprague-Dawley rats. This endocrine background is a critical component of our aged PTSD model.

Statistical Reporting: We have updated the Results section to include independent analysis of sex-dominant hormones. In males, T levels showed a significant elevation following trauma ($t_{(10)} = 2.384$, $P = 0.0383$), whereas in females, E2 levels remained statistically stable ($t_{(10)} = 0.3749$, $P = 0.7156$).

Changes: These clarifications and the corresponding statistical results (*t* values, *df,* and *P* values) have been integrated into Section 3.2 (Plasma Endocrine Profiling)

and are reflected in Figure 3B-C.

Point 15: Lines 284–285: The described effect is not clearly visible in the bar graphs; full statistical details should be provided.

Response: Upon re-evaluating the systemic BDNF data and the corresponding figures, we agree that the previously described 'increase' was statistically overstated. In the revised manuscript, we have corrected this narrative to reflect the statistical reality and have provided the full suite of statistical parameters as requested.

Our revised analysis confirms that while there was a slight numerical elevation in plasma BDNF levels in the PTSD groups (mean 62.08 ng/mL) compared to controls (mean 58.51 ng/mL), this difference did not reach statistical significance. We now explicitly state that peripheral BDNF levels remained statistically stable following the traumatic paradigm in both sexes. This corrected interpretation ensures that our conclusions are strictly grounded in the quantitative evidence.

Changes: The Results section (Section 3.2: Plasma Endocrine Profiling) has been updated to include full Two-way ANOVA details for BDNF.

Point 16: Lines 314–323: The described effects appear to be female-specific based on the figures. This should be clarified in the text and supported with appropriate figure references.

Response: In the revised manuscript, we have thoroughly updated the PET results section to distinguish between shared and sex-dependent metabolic patterns, supported by systematic figure references and Two-way ANOVA results.

Specifically, while we observed a general Group effect of metabolic suppression in the PFC and Hippocampus for both sexes, our quantitative analysis confirmed that the Amygdala (Amyg) hyperactivation is indeed a female-specific metabolic phenotype. Statistical analysis revealed a significant main effect of Sex in the Amyg ($F_{(1, 20)}$ = 16.38, P = 0.0006), and post-hoc comparisons explicitly showed significant hyperactivation only in female PTSD rats (P = 0.0151), whereas no significant change was detected in males (P = 0.1876). We have also relocated the interpretative sentences to

the Discussion section to maintain an objective Results narrative.

Changes: These clarifications, along with the detailed statistical parameters (*F* values, *df*, and *P* values), have been integrated into Section 3.3 (Cerebral Glucose Metabolic Alterations). We have also ensured that all claims are directly supported by references to Figure 4 (A–F) and the corresponding voxel-level data in Tables S2 and S3.

Point 17: Lines 319–320: This sentence appears more suitable for the Introduction or Discussion.

Response: We have removed the interpretative and background-related sentences from the Results section to ensure a more objective and concise presentation of our experimental findings. As suggested, these contents have been relocated to the Discussion section, where they now serve as a foundation for a more in-depth exploration of the neurobiological implications of our findings.

Changes: The corresponding sentences (previously in Lines 319–320) have been integrated into the revised Discussion (Section 4). In the updated narrative, we have expanded these points to discuss how the observed circuit-level metabolic alterations—specifically the divergence in amygdala activity—relate to broader neurobiological frameworks of sex-dependent stress vulnerability in the brain.

Point 18: Line 347: Please clarify whether the reported downward trend is statistically significant and provide the corresponding p value.

Response: In the revised manuscript, we have performed a comprehensive statistical analysis of the temporal dynamics and metabolic rates to clarify these trends.

Specifically, two-way repeated measures ANOVA for the Time-Activity Curves (TACs) identified a highly significant global effect of Group ($F_{(3, 180)} = 21.54$, $P < 0.0001$). For the quantified CMRglu, we observed a robust significant main effect of Group ($F_{(1, 20)} = 16.07$, $P = 0.0007$). Regarding the sex-specific post-hoc comparisons: while male PTSD rats exhibited a non-significant downward trend ($P = 0.3684$), the metabolic reduction in female PTSD rats was more pronounced and approached sta-

tistical significance (*P* = 0.0576). We have explicitly included these *F* values, degrees of freedom, and exact *p* values in the text to provide a transparent representation of the metabolic decline.

Changes: The Results section (Section 3.4 Cerebral Glucose Metabolic Alterations) and the legend for Figure 5 have been updated to include these detailed statistical parameters. The text now clearly distinguishes between the significant global group effect and the specific p-values for sex-based comparisons.

Point 19: Line 350: If correlation analyses were performed, please report the methods and outcomes.

Response: Correlation analyses are indeed crucial for linking our macroscopic metabolic findings to the observed behavioral phenotypes. In the revised manuscript, we have now explicitly detailed the methods and outcomes of these analyses.

We employed Pearson's correlation to evaluate the relationship between regional glucose metabolism (SUV) and the core PTSD-like symptom, namely freezing behavior during the contextual recall test. Our results revealed a robust functional dissociation: metabolic activity in the Hippocampus and PFC was negatively correlated with freezing time across both sexes, suggesting that higher executive and mnemonic metabolic integrity is associated with lower fear expression. Conversely, Amygdala metabolic activity showed a significant positive correlation with freezing behavior, reinforcing its role in fear potentiation. We have also included non-significant correlations (e.g., between whole-brain CMRglu and freezing) in the supplementary material to provide a balanced overview.

Changes: The methodology for correlation analysis has been added to Section 2.8 (Statistical Analysis), and the detailed results (*r* and *P* values) have been integrated into a new Section 3.5(Correlation of Regional Metabolism with Fear Memory). These findings are visually supported by the scatter plots in Figure 6 (A–C) and Figure S4.

**Point 20: Lines 364–371: This section does not fit well within the Results and

appears to repeat information from other sections; would be encourage to improve clarity.

Response: The previously included section (Lines 364–371) primarily described the methodological framework for gene screening and metabolic background set construction, which was redundant as these details are already comprehensively covered in the Materials and Methods (Section 2.7.2).

To improve the clarity and narrative flow of the Results section, we have removed this entire repetitive segment. The updated text now transitions directly from the metabolic deficits observed in PET imaging to the specific transcriptomic alterations, ensuring that the Results section remains focused on our experimental findings rather than methodological justifications.

Changes: The redundant description has been deleted from the revised manuscript. The transition to the transcriptomic results (now in Section 3.6 has been streamlined to provide a more concise and logical presentation.

Point 21: Line 373: If sex differences are claimed, the corresponding main effects or post hoc results should be reported; otherwise, the wording should be softened.

Response: In response, we have overhauled the entire transcriptomic Results section (now Section 3.6) to provide a more rigorous and balanced narrative. Rather than making broad claims of "sex differences" without secondary interaction statistics, we have softened our wording to describe the "sex-specific regulatory patterns" and "spatial heterogeneity" observed across the PFC, Hipp, and Amyg.

Our revised reporting of the transcriptomic pipeline now encompasses:

1. Metabolic Targeting: We clarify how DEGs were intersected with a predefined glucose metabolism gene set to isolate functionally relevant candidates.

2. Functional Profiles: We describe the GO enrichment (BP, CC, and MF) across regions, noting that while many core metabolic processes are shared, their enrichment magnitude and specific gene constituents vary between sexes (e.g., more prominent metabolic signaling enrichment in the male PFC vs. the female Amygdala).

3. Network Architecture: We present the PPI network clustering (MCL and K-means), identifying specific hub genes (as shown in Fig. 8) that orchestrate these metabolic shifts in a region- and sex-dependent manner.

4. By shifting the focus to "comparative enrichment profiles" and "divergent molecular architectures," we believe the manuscript now more accurately represents the high-dimensional nature of the transcriptomic data.

Changes: Section 3.6 has been completely rewritten to reflect this comprehensive analysis. The terminology throughout this section has been softened from "significant sex differences" to "sex-specific patterns" and "region-dependent heterogeneity."

Point 22: Lines 406 and 424: References to the appropriate figures are missing, and some phrasing is not suitable for the Results section.

Response: In our comprehensive revision of the transcriptomics section (now Section 3.6), we have addressed these concerns by ensuring that every biological observation is directly linked to its corresponding visual data.

Changes: Section 3.6 has been systematically updated with precise figure citations. We have also refined the terminology to be more suitable for a Results section, focusing on "differential expression profiles," "network connectivity," and "clustering coefficients" rather than qualitative conclusions.

Figures

Point 23: Fig. 1: Please clarify the purpose of the rat cartoon and explain its meaning in the figure legend; its necessity could be reconsidered.

Response: To improve the clarity and professional presentation of our study design, we have removed the cartoon from Figure 1 as suggested. The revised figure now focuses strictly on the experimental timeline and the procedural workflow, ensuring that the visual emphasis remains on the scientific methodology.

Changes: The rat cartoon has been deleted from Figure 1 in the revised manuscript. The figure legend has also been updated to reflect the streamlined schematic, which now more clearly illustrates the 17-day experimental schedule.

Point 24: Line 269: The information in brackets appears unnecessary in this context.

Response: In the revised manuscript, we have thoroughly streamlined the legend for Figure 2 to provide a more concise and professional description of the behavioral data. All unnecessary bracketed information has been removed, and the terminology has been standardized across all behavioral metrics (e.g., OT%, OE%, and TIC).

Changes: The legend for Figure 2 has been updated as follows:

"Fig. 2. Sex-Specific Behavioral Phenotypes in Aged PTSD Rats. Behavioral assessments of aged male and female rats (n = 6 per group). (A) Representative movement trajectories in the EPM. (B-D) Quantification of (B) percentage of time spent in open arms (OT%), (C) percentage of open arm entries (OE%), and (D) closed arm entries (CAE) in the EPM. (E) Representative trajectories in the OFT. (F-H) Quantification of (F) total distance traveled (TD), (G) central area distance (CR), and (H) time spent in the center (TIC) in the OFT. (I-J) FC test results: (I) distance traveled during fear learning and (J) freezing time during fear memory recall. Data are presented as mean ± SEM. *P < 0.05*, ***P* < 0.01*."

Point 25: Fig. 2: The abbreviation BDNF should be defined. Some functional descriptions of BDNF may not be directly relevant to peripheral measurements and could be omitted from the legend.

Response: In the revised manuscript, we have explicitly defined the abbreviation BDNF (Brain-Derived Neurotrophic Factor) upon its first mention in the figure legend. Furthermore, we have removed the peripheral-unrelated functional descriptions to ensure that the legend strictly describes the presented endocrine and biomarker data. We have also clarified the specific statistical tests (unpaired t-test vs. Two-way ANOVA) used for each panel to enhance methodological transparency.

Changes: The legend for Figure 3 (formerly Figure 2) has been streamlined as follows:

"Fig. 3. Systemic endocrine and neurotrophic factor profiles following traumatic stress. Plasma endocrine and biomarker profiles of rats (n = 6 per group). (A) Plasma

testosterone (T) levels in male rats. (B) Plasma estradiol (E2) levels in female rats. (C) Plasma corticosterone (CORT) levels across the four experimental groups. (D) Circulating brain-derived neurotrophic factor (BDNF) levels in both male and female rats. Data are presented as mean ± SEM. $P < 0.05$. Statistical significance was determined by unpaired Student's t-test (A, B) or Two-way ANOVA followed by Tukey's post-hoc test (C, D)."

Point 26: Fig. 5: Please clarify abbreviations (e.g., BP, CC, MF) that are not shown directly in the figure.

Response: In the revised manuscript, we have updated the legend for the GO enrichment analysis (now Figure 7) to explicitly define the abbreviations for the three main ontologies. Specifically, we have clarified that BP stands for Biological Process, CC for Cellular Component, and MF for Molecular Function.

Changes: The legend for Figure 7 has been revised as follows:

Fig. 7. GO functional enrichment analysis of genes related to different brain regions of PTSD model rats. (A-C) Enrichment results in the PFC (A), Hipp (B), and Amyg (C). The analysis incorporates differentially expressed genes from both male and female samples, categorized into Biological Process (BP), Cellular Component (CC), and Molecular Function (MF). The X-axis represents $-\log_{10}$(padj), where larger values indicate more significant enrichment.

Point 27: Fig. S1: The schematic is currently of limited informativeness. Adding more details (e.g., timing of stressors, age and sex of animals used, experimental timeline) would increase its value. Please also consider consistent terminology (e.g., 'restraint' vs. 'bondage')."

Response: We have completely redesigned this figure (now promoted to Figure 1 in the main manuscript) to serve as a comprehensive visual guide for our methodology.

The revised Figure 1 now incorporates the following critical details:

1. Experimental Timeline: A clear 17-day schedule, including adaptation, SPS+FS modeling, incubation, behavioral testing, and PET scanning phases.

2. Detailed Modeling Procedure: Specification of the Single Prolonged Stress plus Footshock (SPS+FS) protocol, including precise timings (e.g., 2h restraint, 20min swimming, 1mA 4s footshock).

3. Animal Characteristics: Explicit indication of the use of 15-month-old aged rats of both sexes (♂/♀).

4. Neuroimaging Protocols: Detailed breakdowns of both static and dynamic FDG-PET scanning sequences.

5. Standardized Terminology: As suggested, we have corrected the terminology, replacing informal terms like "bondage" with the standardized academic term "restraint stress.".

Changes: The previous supplementary schematic has been replaced by the updated Figure 1 in the main text. The figure legend has been expanded to provide a complete description of the multi-stage experimental workflow shown in panels A–D.

Point 28: Additionally, the authors are encouraged to clarify why hippocampal and amygdala transcriptomic data were moved to the Supplementary section. Given that the behavioral paradigm primarily assesses contextual fear memory, hippocampal alterations may be central, while PFC and amygdala changes could be interpreted as modulatory rather than secondary or less important.

Response: We fully agree that the Hippocampus (Hipp) and Amygdala (Amyg) are not secondary, but rather primary nodes in the neural circuitry governing contextual fear memory and its dysregulation in PTSD.

In the revised manuscript, we have re-integrated the complete transcriptomic datasets for both the Hippocampus and Amygdala into the main Results section (now detailed in Section 3.6). By presenting the molecular alterations of the PFC, Hipp, and Amyg side-by-side, we now offer a more holistic and integrated view of the brain-

wide metabolic and transcriptomic disruptions. This structural change allows us to better discuss how hippocampal deficits (central to memory) and amygdala hyperactivation (modulatory to fear expression) synergistically contribute to the sex-dependent phenotypes observed in our aged rat model.

Changes: The transcriptomic analysis of the Hippocampus and Amygdala has been moved from the Supplementary Material back to the main text.

Discussion

Point 29: Line 427: The use of the term 'aging' may be misleading when only a single age group is analysed; rephrasing is recommended."

Response: We agree with the reviewer that the term "aging" typically implies a longitudinal process or a comparison across multiple age cohorts, which was not the primary design of this study. To ensure scientific precision, we have revised our terminology throughout the manuscript, particularly in the concluding remarks.

We have replaced "aging" with more accurate descriptors such as "the aged brain" or "15-month-old aged rats" to clarify that our findings reflect the neurobiological state at a specific late-adult developmental stage. This rephrasing correctly frames our conclusions within the context of the established aged PTSD model rather than the biological process of aging itself.

Changes: The concluding sentence of the Results/Discussion transition (formerly Line 427) has been revised to:

"By integrating macro-scale neuroimaging with transcriptomics, this study offers a potential framework for understanding the neuroenergetic basis of sex-specific PTSD phenotypes in the aged brain. Our observations suggest that 15-month-old female rats may exhibit heightened behavioral vulnerability..."

Point 30: Lines 431–432: Please acknowledge the lack of changes in peripheral hormone levels, if applicable.

Response: In the revised Discussion, we have explicitly acknowledged and addressed the relative stability of certain peripheral hormone levels in the aged female

PTSD model, providing a biological context for these observations.

Specifically, we have clarified that while male PTSD rats exhibited significant elevations in plasma Corticosterone (CORT) and Testosterone (T), these fluctuations were notably absent in the female cohort. We interpret the stable CORT levels in 15-month-old female rats as a reflection of age-related alterations in HPA axis reactivity, where senescent females may maintain a baseline-like endocrine state despite traumatic stress. Furthermore, we have acknowledged that the stable but low levels of Estradiol (E2) (as shown in Fig. 3B-C) likely represent the exhaustion of classical neuroprotective buffers due to reproductive senescence. This lack of peripheral endocrine flexibility in females stands in contrast to the compensatory responses seen in males and underscores the unique bioenergetic vulnerability of the aged female brain.

Changes: The Discussion section (Section 4) has been expanded to include a dedicated analysis of these peripheral endocrine profiles. We have integrated references to our results (Figure 3) and relevant literature [43-46] to justify why this lack of hormonal change is a critical feature of the aged female stress response.

Point 31: Line 438: The data do not fully support direct conclusions regarding abnormal neuroplasticity; this claim should be moderated.

Response: We agree with the reviewer that our current datasets, while comprehensive in terms of neuroimaging and transcriptomics, do not provide direct structural or electrophysiological evidence of altered neuroplasticity. In accordance with this suggestion, we have removed the definitive claim regarding "abnormal neuroplasticity" to maintain a more rigorous and evidence-based narrative.

In the revised Discussion, we have re-framed this section to focus on the "potential for neuroanatomical and functional remodeling" as inferred from the significant dysregulation of metabolism-related genes and regional glucose utilization. By softening the language and characterizing these changes as "precursors to" or "indicators of" compromised synaptic integrity rather than confirmed plasticity deficits, we ensure that our conclusions remain strictly within the scope of the presented data.

Changes: The text in the Discussion (formerly Line 438) has been thoroughly

moderated.

Point 32: Line 443: Phrases such as 'stark contrast' could be replaced with more neutral scientific language."

Response: We agree that objective and neutral language is essential for a rigorous presentation of comparative data. In the revised manuscript, we have conducted a thorough linguistic review of the entire text to ensure a more balanced and professional tone.

Specifically, we have replaced subjective phrases such as "stark contrast" with more precise, neutral alternatives like "notable divergence," "significant difference," or "distinct patterns." In the context of our sex-dependent findings, we now describe the results by highlighting the specific directions and magnitudes of change in each cohort, allowing the statistical evidence to speak for itself without over-emphasizing the degree of difference through qualitative adjectives.

Changes: The phrasing in Line 443 (and throughout the Discussion) has been updated. For instance, the comparison between male and female metabolic responses now uses more formal academic structures, such as "In contrast to the patterns observed in males, female rats exhibited..." or "A significant divergence was noted between..."

Point 33: Line 452: The potential role of estradiol in aged females and its reduced neuroprotective effects after menopause should be discussed more cautiously.

Response: We agree that the neuroprotective efficacy of estradiol (E2) is highly dependent on the reproductive stage and the functional state of estrogen receptors.

In the revised Discussion, we have moderated our claims to reflect the biological reality of reproductive senescence. We now explicitly discuss that while E2 is conventionally neuroprotective in younger cohorts, this efficacy appears to diminish significantly in our 15-month-old female model. By highlighting our observation of stable but low serum E2 levels (Fig. 3C), we suggest that the aged female brain may have

passed the "window of opportunity" for classical neuroprotection, leaving it more vulnerable to traumatic injury. This refined narrative avoids overstating the protective role of residual E2 and instead focuses on the age-related attenuation of endocrine buffering, which aligns with the observed metabolic and behavioral deficits.

Changes: The Discussion section (Section 4) has been updated with a more nuanced analysis of the E2 data.

Point 34: Lines 498–510: Statements regarding neuroinflammation appear speculative, as no direct measures were performed; this section should be toned down.

Response: We fully agree with the reviewer's assessment that without direct histological or biochemical markers of neuroinflammation (such as Iba1 or cytokine assays), conclusions in this area remain speculative. Consequently, we have removed all speculative statements regarding neuroinflammation from the Discussion.

In the revised manuscript, we have refocused this section (Lines 498–510 in the original, now expanded in Section 4) on the mechanistic interpretation of our transcriptomic and PET imaging data, for which we have direct evidence. We now provide a detailed analysis of the central insulin and AMPK signaling networks. Specifically, we discuss how the coordinated dysregulation of key metabolic sensors and enzymes (e.g., *Gsk3b*, *Hk1*, *Ppargc1a*, and *Prkaa2*) reflects a state of "metabolic emergency" and mitochondrial strain. By framing our findings within the context of bioenergetic reprogramming and metabolic signaling "tuning," we offer a more grounded and evidence-based explanation for the observed sex-dependent PTSD phenotypes.

Changes: The section discussing neuroinflammation has been deleted. It has been replaced with a rigorous discussion of the Insulin-AMPK axis as a central metabolic-behavioral nexus. This revised narrative integrates our findings on rate-limiting glycolytic enzymes and energy sensors to explain the potential molecular substrates of the observed metabolic deficits, supported by relevant literature [50–60].

Point 35: Lines 511–523: The conclusion would benefit from a clearer sum-

mary of the main findings rather than focusing primarily on future research directions. Highlighting the limitations of the present study is also encouraged.

Response: In the revised manuscript, we have completely restructured the concluding paragraph to provide a concise synthesis of our primary findings while maintaining an objective perspective on the study's constraints.

The revised conclusion now clearly highlights:

1. Core Findings: The demonstration of sex-dependent PTSD-like phenotypes in 15-month-old rats, characterized by a unique decoupling from peripheral hormonal status.
2. Multimodal Integration: The alignment between macroscopic metabolic suppression (via FDG-PET) and molecular-level reprogramming of the central Insulin-AMPK signaling axis (specifically involving Gsk3b, Hk1, and Prkaa2).
3. Study Limitations: We have added a dedicated discussion on the inherent limitations of our study, including the use of bulk tissue transcriptomics (which lacks cell-type resolution), the cross-sectional design (precluding longitudinal tracking), and the need for protein-level functional validation.
4. By acknowledging these limitations and focusing on the identified neuroenergetic framework, we believe the conclusion now provides a more balanced and rigorous final statement.

Changes: The Conclusion section (Lines 511–523 in the original, now updated at the end of Section 4) has been rewritten to reflect this comprehensive summary.

Response to Reviewers

Manuscript Title: Sex-Dependent Neurobiological and Metabolic Dysfunction in an Aged Rat Model of Post-Traumatic Stress Disorder

Manuscript ID: [COMMSBIO-25-11210-T]

Dear Editor and Reviewers,

We would like to express our sincere gratitude to the reviewers for their insightful comments and constructive suggestions, which have significantly improved the quality and rigor of our manuscript. We have carefully addressed each point raised by the reviewers and revised the manuscript accordingly.

Reviewer #2 (Remarks to the Author):

Introduction

Point 1: For which reason a number of genes mentioned in the introduction since an open approach was adopted? They should be removed.

Response: We completely agree with the reviewer's observation. Since this study employs an unbiased, open-ended transcriptomic approach to identify molecular signatures of metabolic dysfunction, mentioning specific candidate genes in the Introduction was premature and logically inconsistent with an exploratory discovery pipeline.

To rectify this, we have removed all references to specific genes (such as *Gsk3b*, *Hk1*, etc.) from the Introduction. The revised Introduction now focuses on the broader biological significance of neuroenergetic failure in PTSD and the rationale for using a multi-omics approach to bridge the gap between macroscopic imaging and microscopic molecular shifts. This adjustment ensures that our study's "discovery-based" nature is accurately reflected in the narrative flow.

Changes: The Introduction has been streamlined to focus on the research gaps in aged PTSD models and metabolic signaling, without pre-specifying gene targets. All specific gene names previously mentioned have been deleted to maintain the integrity of the open-approach framework.

Materials and Methods

Point 2 & 3: Methods: The overall number of animals should be mentioned.The reason why controls were singly housed should be detailed as well as which procedures were carried out on them."

Response: We thank the reviewer for these important methodological queries. We have now provided comprehensive details in the revised Materials and Methods (Section 2.1) to ensure full transparency regarding animal welfare and experimental control.

Changes: The "Animals " section has been expanded to include the total count, the justification for single housing, and the specific handling protocols for the control cohorts.

Point 4 & 5: I understand that the rats were acquired as 15 mo old and the stress carried out immediately after acclimatation, is this correct?"And when was the pet scan carried out? Maybe supplementary figure S1 could be expanded to include timelines of all activities."

Response: The reviewer's understanding is correct. We utilized 15-month-old aged rats, and the stress induction (SPS+FS protocol) was initiated on Day 8, immediately following a 7-day acclimatization period. To provide a clear and comprehensive overview of all experimental activities, we have completely redesigned the schematic diagram. This figure has been promoted from the supplementary material to the main text as Figure 1.

As illustrated in the expanded Figure 1A, the experimental sequence was as follows:

1. Days 1–7 (Adaptation): Following acquisition at 15 months of age, all rats underwent 7 days of environment and handling acclimatization.
2. Day 8 (SPS+FS Modeling): The stress protocol was performed.
3. Days 9–15 (Incubation): A 7-day post-stress incubation period to allow for the development of PTSD-like phenotypes.
4. Day 16 (Behavioral Assessment): EPM, OFT, and Fear Conditioning tests were conducted.

5. Day 17 (PET Scanning): FDG-PET neuroimaging (including both static and dynamic protocols, as detailed in Figure 1D) was performed on the final experimental day.

Changes: We have replaced the previous supplementary schematic with a much more detailed and informative **Figure 1** in the main manuscript. This new figure integrates the timelines for modeling, behavioral testing, and neuroimaging assessments into a single, intuitive framework.

Point 6 & 7: Details of the professional video tracking system analysis software are to be included. Dimensions of the open field arena need specification.

Response: In the revised Materials and Methods (Sections 2.3), we have now included the specific details of the video tracking system and the precise dimensions of all behavioral apparatuses to ensure full methodological transparency.

Specifically, the following information has been added:

1. Video Tracking Software: All animal movements, trajectories, and freezing behaviors were recorded and quantified using the DigiBehv professional video tracking system (Shanghai Jiliang Software Technology Co., Ltd., China). This system allowed for automated, objective analysis of key metrics such as distance traveled, zone-specific residence time, and freezing duration.

2. OFT Dimensions: The Open Field Test was conducted in a white, square arena with dimensions of 100 × 100 × 50 cm.

3. EPM and Fear Box Details: We have also specified that the EPM was elevated 50 cm above the floor and detailed the definition of freezing behavior used by the software (absence of all movement except for respiration) to quantify contextual fear memory.

Changes: The behavioral assessment sections have been expanded to include the manufacturer details of the tracking software and the exact physical dimensions of the OFT and EPM apparatuses.

Point 8: Line 149 should be 'entries', not 'time'.

Response: We have corrected the terminology in the revised Materials and Methods (Section 2.3) The previous wording incorrectly used "time" in a context where "entries" was the appropriate metric. In the updated manuscript, we have replaced "time" with "entries" to accurately reflect the quantification of animal movements into the maze arms.

The parameters analyzed now clearly distinguish between the number of open arm entries, time spent in open arms, and closed arm entries. These raw metrics were subsequently used to calculate the percentage of open arm entries (OE%) and the percentage of time spent in open arms (OT%) as standardized measures of anxiety-like behavior.

Changes: The description of EPM parameters has been corrected. The revised sentence now reads: Parameters analyzed included the number of open arm entries, time spent in open arms, and closed arm entries."

Point 9: Centrifugation speed should be reported in g, not rmp.

Response: We agree that reporting centrifugation intensity in relative centrifugal force (g) is essential for the reproducibility of the protocol. In the revised manuscript, we have added a dedicated section (Section 2.6 Tissue Processing and RNA Extraction) and updated all centrifugation parameters to g.

Changes: A new subsection on tissue processing and RNA extraction has been integrated into the Materials and Methods. All references to "rpm" have been removed and replaced with the corresponding "g" values to comply with standard laboratory reporting practices.

Point 10: The method for ELISA assays is poorly described. Since manufacturer's instructions are not available in English on the web, the authors are invited to describe the procedure in detail, in particular including the amount of plasma and dilution, how 'the actual concentration of each analyte was calculated based on the established standard curve', which curve fitting model was adopted.

Response: We recognize that international reproducibility depends on clear procedural descriptions. In the revised Materials and Methods (Section 2.2), we have now fully detailed the ELISA protocols for T, E2, CORT, and BDNF.

Key technical details added to the manuscript include:

1. Sampling and Dilution: 10 μL of plasma sample was added to 40 μL of sample diluent, achieving a 5-fold dilution for each assay.
2. Standard Curve Fitting: To ensure the highest accuracy in quantifying analyte concentrations, we utilized a four-parameter logistic (4PL) regression model to generate the standard curves. This non-linear model is the gold standard for ELISA data as it accounts for the sigmoidal relationship between optical density (OD) and concentration.
3. Measurement Parameters: We have included the detection wavelength (450 nm) and the specific Catalog Numbers for the kits from Shanghai Affandi Industrial Co., Ltd. (T: A109234; E2: A108219; CORT: A107689; BDNF: A107739).
4. Temporal Control: We also clarified that blood collection was restricted to a specific time window (09:00–11:00) to control for circadian variations in hormone levels.

Changes: The "Endocrine and Biomarker Analysis" section has been significantly expanded to incorporate these procedural details, ensuring that the methodology is transparent and reproducible despite the limited online availability of the original manufacturer's manual in English.

Point 11: The method for dissection of the three brain regions should be explained.

Response: Precise anatomical dissection is indeed critical for the validity of our region-specific transcriptomic findings. In the revised Materials and Methods (Section 2.6) we have explicitly described the dissection procedure.

Changes: Section 2.6 has been updated to include the anatomical reference used for dissection and the specific thermal conditions maintained during tissue collection.

Point 12: Which is 'appropriate statistical software' mentioned at line 229?

Response: In the revised manuscript, we have replaced the vague term "appropriate statistical software" with the specific versions of the platforms employed for our analyses. All primary statistical analyses and graphing were performed using GraphPad Prism (v9.0) and R (v4.2.1).

Changes: Section 2.8 has been rewritten to explicitly name the software used and detail the comprehensive statistical workflow, ensuring the rigor and reproducibility of our data interpretation.

Point 13: The method for p value adjustment in transcriptomic analysis is either non-descript or missing. If the latter, the results are likely to contain a high number of false discoveries, therefore the value of the results is questionable.

Response: We fully acknowledge the reviewer's concern regarding the risk of false discoveries in high-throughput transcriptomic data. Rigorous statistical correction is indeed essential for the validity of differential expression analysis. In the revised manuscript, we have now explicitly detailed our bioinformatics pipeline and the specific methods used to control the False Discovery Rate (FDR).

Changes: Section 2.7.2 has been expanded to include the specific bioinformatics tools (fastp, HISAT2, FeatureCounts, and DESeq2) and the exact parameters used for *P* value adjustment and DEG filtering.

Results

Point 14 & 15: Results: for all results, the statistical results in the two-way ANOVA tests for should be reported as F and degrees of freedom.To evaluate EPM, OT% and OE% should be displayed and analysed.

Response: We fully agree with the reviewer that reporting the complete statistical parameters (*F* values and degrees of freedom) is essential for a transparent and rigorous interpretation of the data. In the revised manuscript, we have updated all behavioral, PET, and biochemical results to include these values.

As requested, we have also refined our analysis of the Elevated Plus Maze (EPM)

by focusing on the standard anxiety indicators: the percentage of time spent in open arms (OT%) and the percentage of open arm entries (OE%).

Changes: Figures 2B and 2C have been updated to display OT% and OE%, respectively. The corresponding Results text and figure legends now include the full *F* values, *df*, and *P* values for all Two-way ANOVA and post-hoc comparisons.

Point 16: Whereas the pathway enrichment is clearly described, the genes discussed as regulated appear to have been manually selected without a specified criterion.

Response: We agree that a transparent, criteria-based pipeline is essential to avoid selection bias. In the revised manuscript, we have now explicitly defined the three-stage hierarchical filtering process used to identify the key genes for discussion. As detailed in Section 2.7.2.

Changes: Section 2.7.2 has been rewritten to formally state these selection criteria. We believe this systematic approach ensures that the discussed genes represent the most statistically robust and functionally central regulators of the observed metabolic dysfunction.

Data Availability

Point 17: Transcriptomic data should be made available on dedicated databases.

Response: We fully agree with the importance of data accessibility and transparency in genomic research. In accordance with the reviewer's recommendation, the raw and processed transcriptomic datasets generated in this study have been deposited in the Gene Expression Omnibus (GEO) database.

The record is currently in private status to facilitate anonymous peer review, with a scheduled public release date of **April 5, 2027**. During the review process, the data can be accessed by editors and reviewers using the following secure token:

- GEO Accession Number: GSE327211

- Reviewer Access Token: qdetumeavhytduh
- Access Link: https://www.ncbi.nlm.nih.gov/geo/query/acc.cgi?acc=GSE327211

To review the record, please navigate to the link above and enter the provided token into the required field. This ensures that our molecular findings are fully traceable and verifiable.

Changes: A **Data Availability Statement** has been added to the manuscript, specifying the GEO accession number and confirming that all transcriptomic data will be publicly available upon publication or by the specified release date.

Discussion

Point 18: Discussion of available data from the literature about transcriptomics in PTSD rat models should be carried out.

Response: In the revised Discussion (Section 4), we have integrated a comparative analysis between our results and existing literature on rodent PTSD models.

Specifically, we have discussed how our findings align with and expand upon current knowledge:

1. Conserved Pathways: We acknowledge that multi-species transcriptomic meta-analyses and hippocampal-specific profiling in other PTSD models have consistently identified disruptions in bioenergetic and synaptic pathways [53, 54].
2. Unique Contribution of the Aged Model: While previous studies have laid the groundwork, our data in 15-month-old rats uniquely underscore a systemic reprogramming of insulin-AMPK metabolic signaling cascades.
3. Mechanistic Alignment: We discuss our observed dysregulation of the Insulin-AMPK axis (e.g., Gsk3b, Hk1, Prkaa2) in the context of "metabolic emergency" and mitochondrial insufficiency. This aligns with emerging evidence that PTSD is characterized by a fundamental failure in neuroenergetic regulation, but adds a novel layer regarding the sexual dimorphism of these shifts in the aged brain.

Changes: The Discussion section has been expanded to include references to key transcriptomic studies [53-60], establishing a clearer link between our region-

specific results and the established molecular signatures of PTSD in the literature.
