# Supplementary material for: Sex-dependent neurobiological and metabolic dysfunction in an aged rat model of post-traumatic stress disorder
Source: Commun Biol. 2026 Jun 19;9:1300. doi: 10.1038/s42003-026-10536-x (PMC13642903; doi:10.1038/s42003-026-10536-x)
Supplement: Supplementary file 2 [file 42003_2026_10536_MOESM2_ESM.pdf]

Supplementary Information

Sex-Dependent Neurobiological and Metabolic Dysfunction in an Aged Rat Model of Post-Traumatic Stress Disorder

*Yubo Hu, Wentao Hu, Yueru Zhong, Yu Liu, Peng Xiao**

Department of Biomedical Engineering, Huazhong University of Science and Technology, Wuhan 430074, PR China.

E-mail: xiaopeng@hust.edu.cn (Peng Xiao)

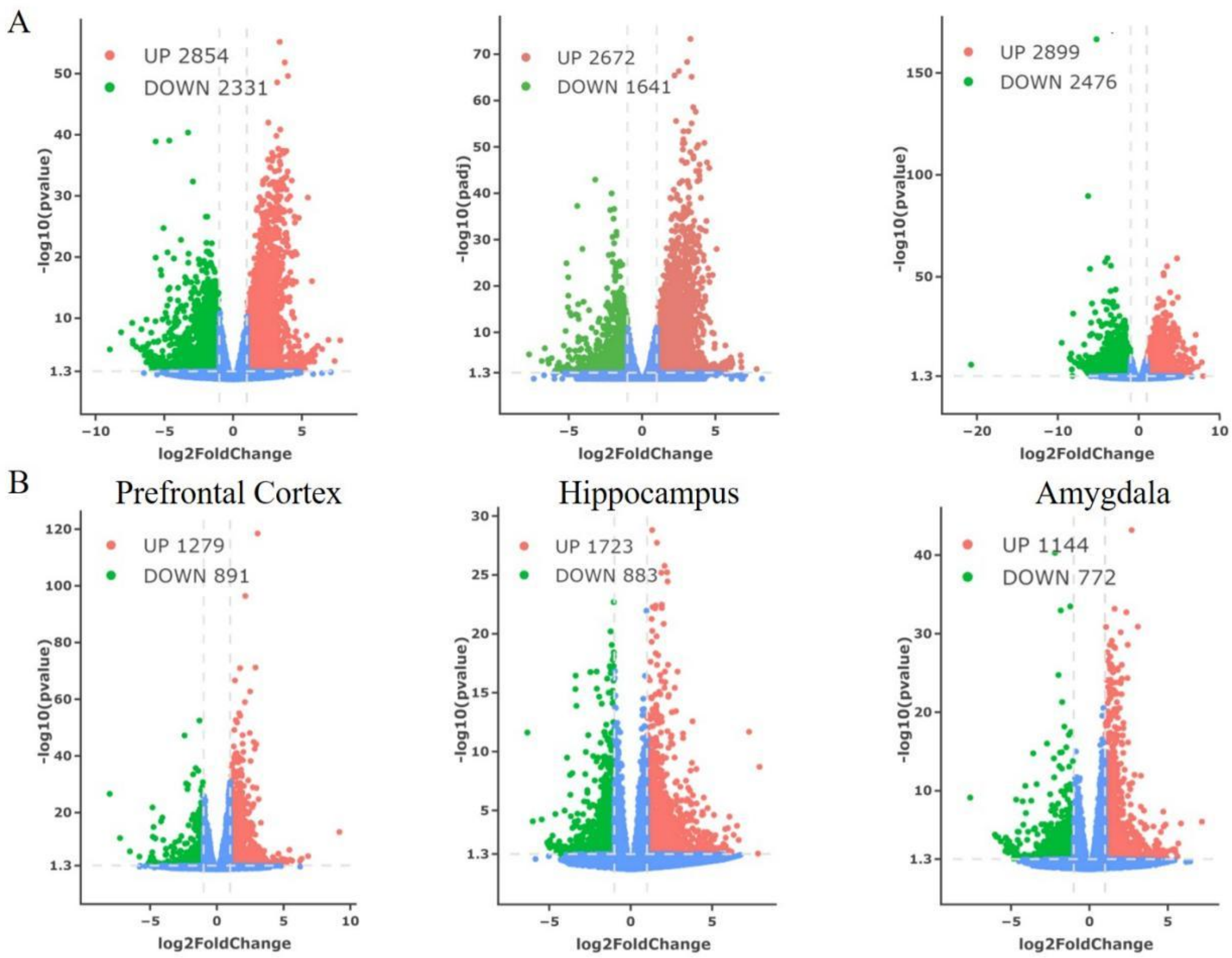

Fig. S1. Screening of differentially expressed genes in brain regions using the DEGs standard. (A is male, B is female. From left to right: prefrontal cortex, hippocampus, amygdala)

Table S1. Blood glucose measurement in rats.

SD Rat	male		female	
	CK	PTSD	CK	PTSD
Blood sugar level (mmol/L)	5.25±0.45	6.28±0.81	4.3±0.05	5.16±0.38

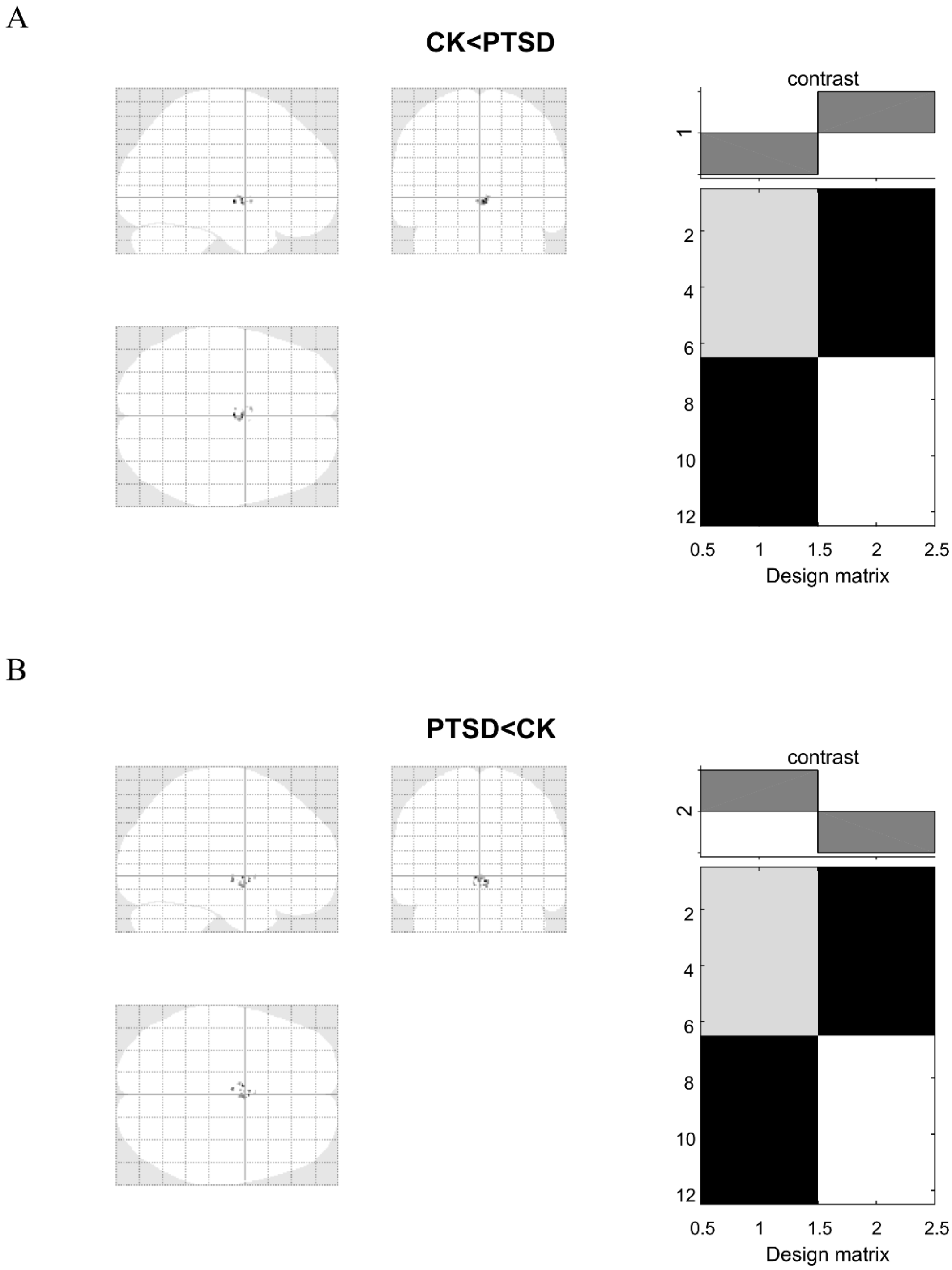

Fig. S2. Contrast analysis of glucose metabolism differences in male PTSD rats. The statistical threshold was set at a T value of 4.1, corresponding to $p<0.001$ (uncorrected). Panel A (glass brain) presents the PTSD > Control metabolic contrast (−1 1 vector), which reveals brain regions with significantly increased glucose metabolism (functional hyperactivity). The primary significant difference is localized to the amygdala (Amyg). Panel B (glass brain) presents the Control > PTSD metabolic contrast (1 −1 vector), which identifies regions where the PTSD model group showed significantly reduced function (metabolic suppression). The major significant differences are concentrated in the prefrontal cortex (PFC) and the hippocampus (Hipp).

Table S2. Significant differences and location of key brain regions in males.

Brain region	Cluster-level			Peak-level		Coordinates		
	FWE corr.	uncorr.	voxels	FWE corr.	uncorr.			
Prefrontal Cortex	0.069	0.030	717	0.540	<0.001	-0.97	3.16	-4.67
Hippocampus	0.009	0.004	1452	0.049	<0.001	3.40	-4.90	-3.35
Amygdala	0.068	0.029	722	0.512	<0.001	3.47	-3.85	-9.68

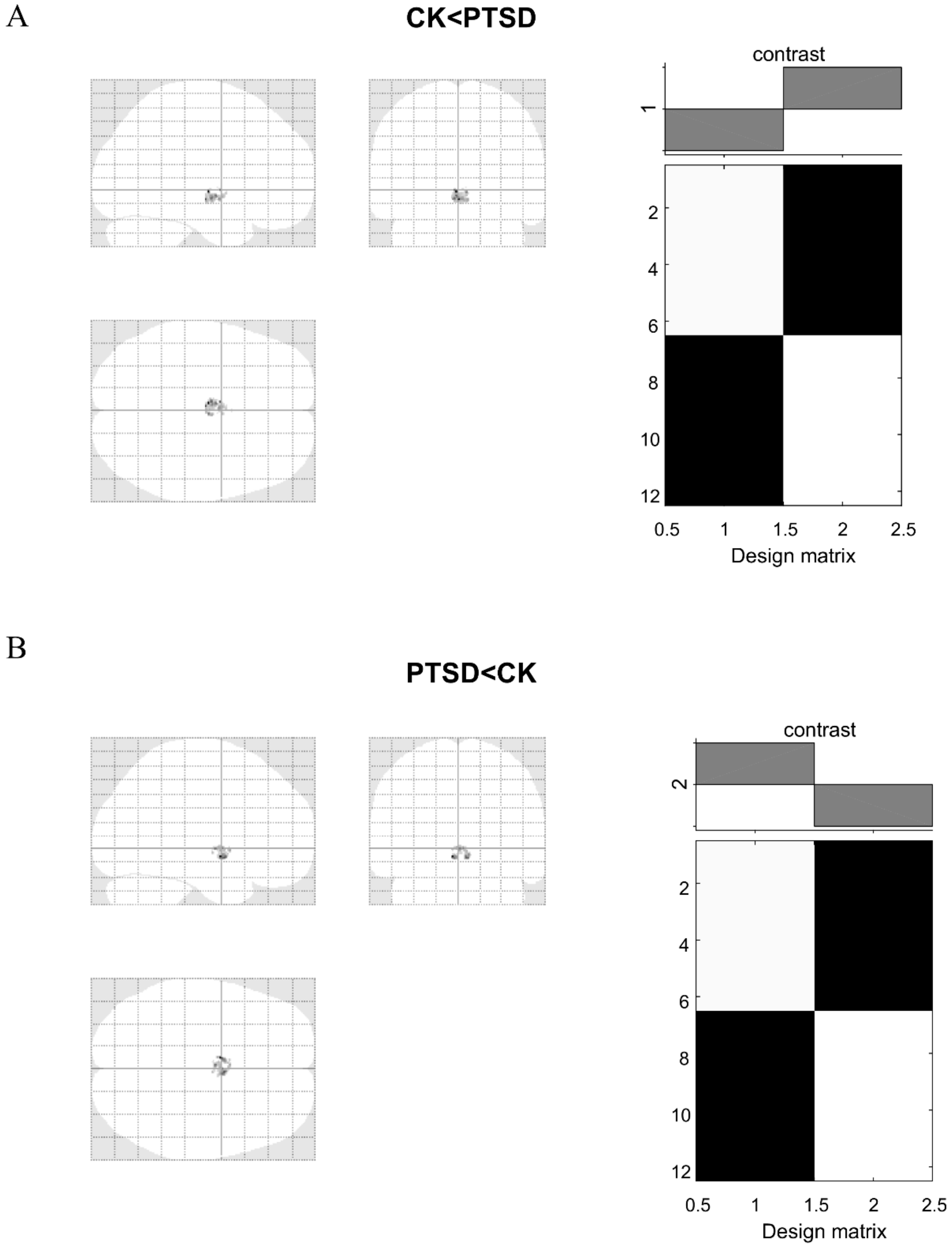

Fig. S3. Contrast analysis of glucose metabolism differences in female PTSD rats. The statistical

threshold for all panels was set at a T value of 4.1, corresponding to p<0.001 (uncorrected). Panel A (glass brain) presents the PTSD > Control metabolic contrast (−1 1 contrast vector), which clearly reveals brain regions with significantly increased glucose metabolism (functional hyperactivity) in the PTSD group. The primary significant difference is localized to the amygdala (Amyg). Panel B (glass brain) presents the Control > PTSD metabolic contrast (1 −1 contrast vector), which identifies regions where the PTSD model group showed significantly reduced function (metabolic suppression). The major significant differences are concentrated in the prefrontal cortex (PFC) and the hippocampus (Hipp).

Table S3. Significant differences and location of key brain regions in females.

Brain region	Cluster-level			Peak-level		Coordinates		
	FWE corr.	uncorr.	voxels	FWE corr.	uncorr.			
Prefrontal Cortex	0.349	0.189	251	0.052	<0.001	1.03	1.98	-4.53
Hippocampus	<0.001	<0.001	7244	<0.001	<0.001	-4.80	-6.29	-5.92
Amygdala	<0.001	<0.001	3123	<0.001	<0.001	5.07	-2.81	-8.50

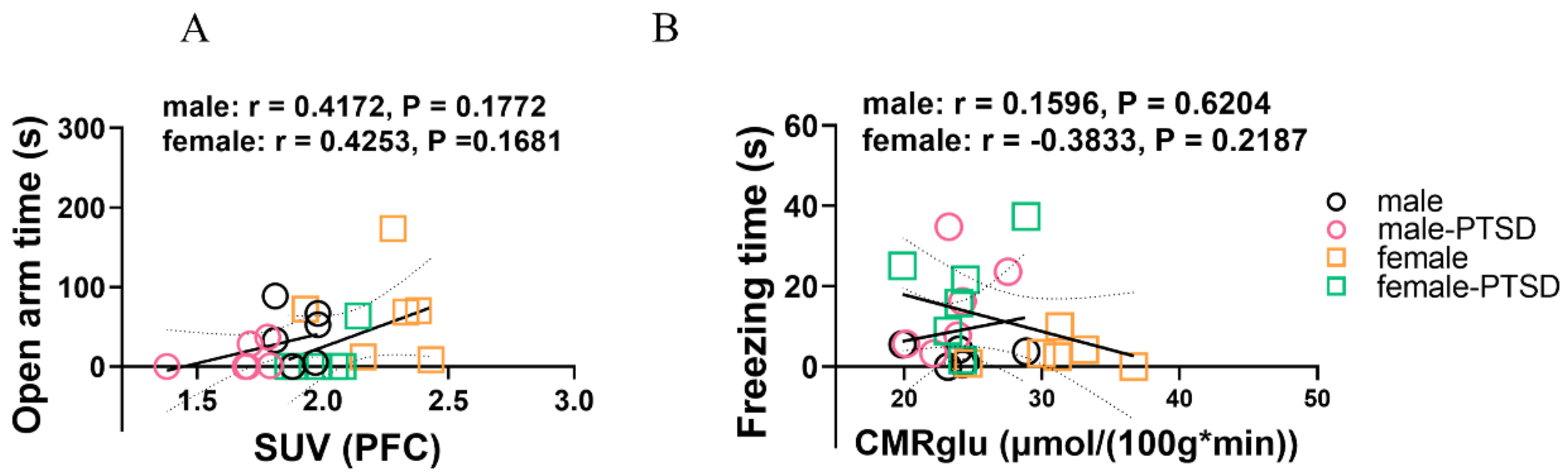

Fig. S4. Correlation between macroscopic metabolic deficits and PTSD-like behavioral phenotypes. (A) Pearson correlation analysis revealed that Standardized Uptake Value (SUV) in the prefrontal cortex (PFC) was not significantly correlated with the percentage of time spent in the open arms during the Elevated Plus Maze (EPM) test in 15-month-old rats. (B) Similarly, no significant correlation was observed between whole-brain Cerebral Metabolic Rate of glucose (CMRglu) and freezing behavior during the Contextual Fear Conditioning (CFC) test.

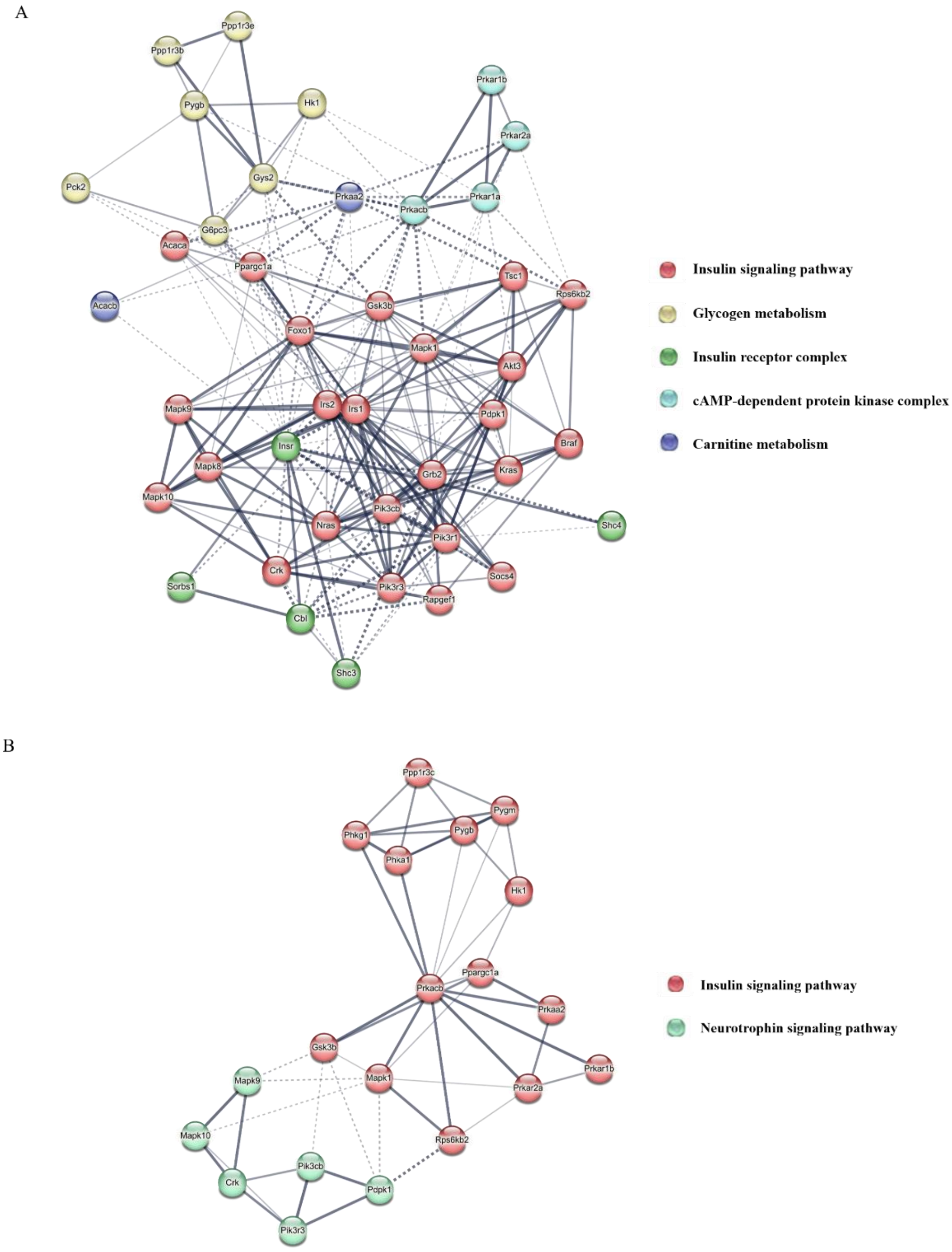
A
Ppp1r3e
Ppp1r3b
Pygb
Hk1
Prkar1b
Prkar2a
Pck2
Gys2
Prkaa2
Prkacb
Prkar1a
G6pc3
Acaca
Ppargc1a
Tsc1
Rps6kb2
Acacb
Gsk3b
Foxo1
Mapk1
Akt3
Mapk9
Irs2
Irs1
Pdpk1
Insr
Braf
Mapk8
Grb2
Kras
Mapk10
Pik3cb
Shc4
Nras
Pik3r1
Crk
Socs4
Sorbs1
Pik3r3
Rapgef1
Cbl
Shc3
Insulin signaling pathway
Glycogen metabolism
Insulin receptor complex
cAMP-dependent protein kinase complex
Carnitine metabolism
B
Ppp1r3c
Pygm
Phkg1
Pygb
Phka1
Hk1
Ppargc1a
Prkacb
Prkaa2
Gsk3b
Prkar1b
Mapk9
Mapk1
Prkar2a
Mapk10
Rps6kb2
Pik3cb
Crk
Pdpk1
Pik3r3
Insulin signaling pathway
Neurotrophin signaling pathway

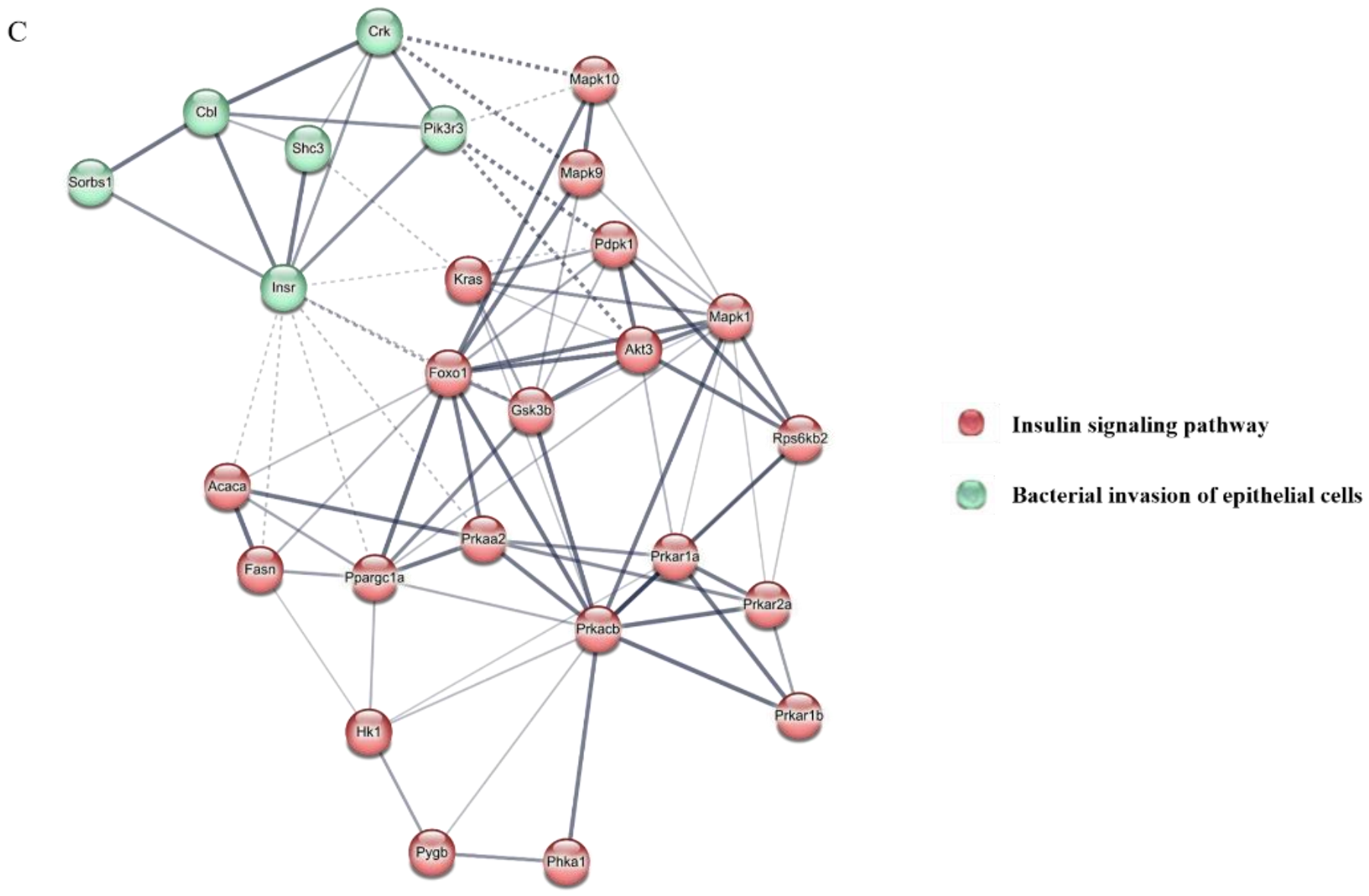
C
Crk
Mapk10
Cbl
Pik3r3
Shc3
Sorbs1
Mapk9
Pdpk1
Insr
Kras
Mapk1
Akt3
Foxo1
Gsk3b
Rps6kb2
Acaca
Prkaa2
Prkar1a
Fasn
Ppargc1a
Prkar2a
Prkacb
Prkar1b
Hk1
Pygb
Phka1
Insulin signaling pathway
Bacterial invasion of epithelial cells

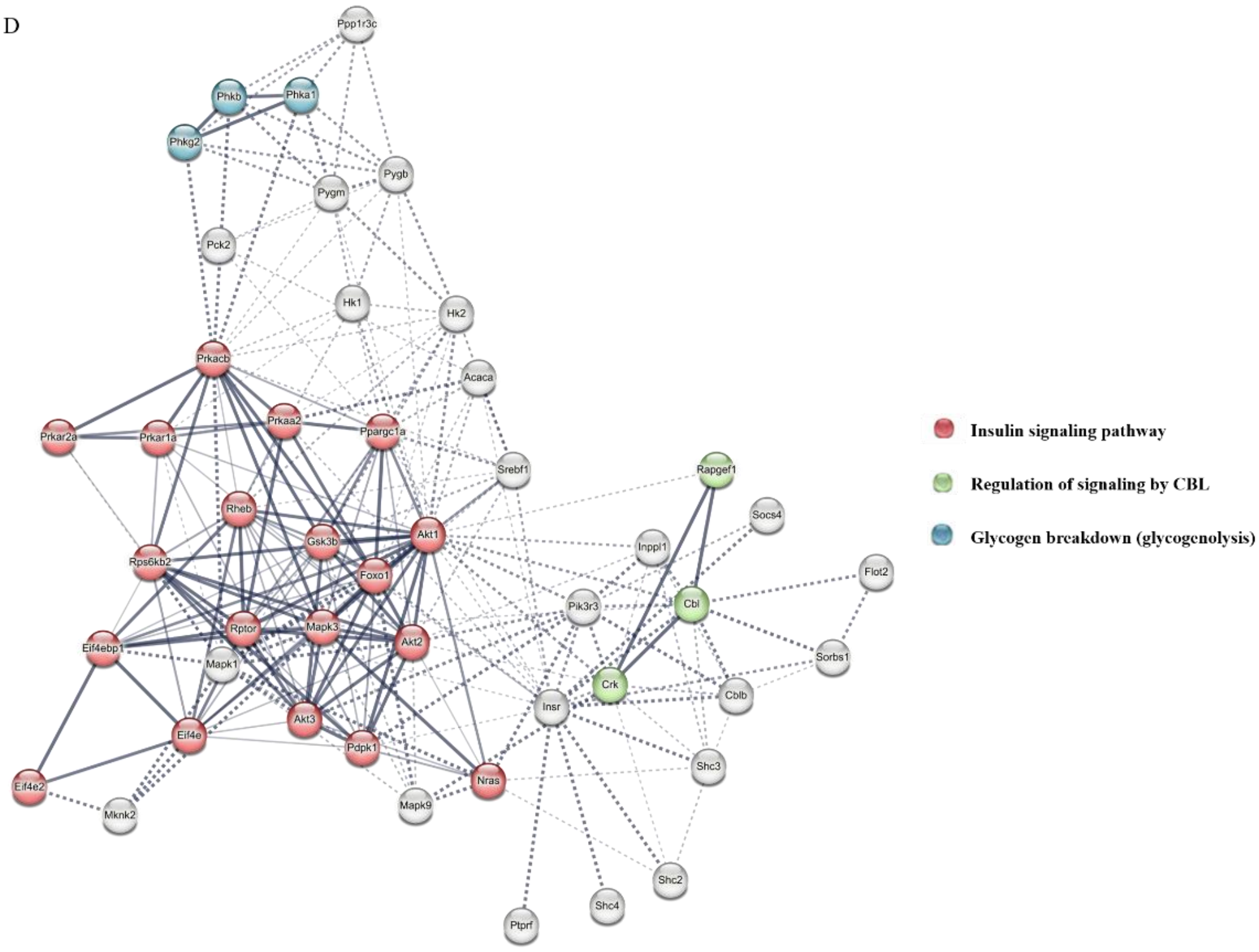
D
Ppp1r3c
Phkb
Phka1
Phkg2
Pygm
Pygb
Pck2
Hk1
Hk2
Prkacb
Acaca
Prkar2a
Prkar1a
Prkaa2
Ppargc1a
Srebf1
Rapgef1
Socs4
Rheb
Akt1
Inppl1
Flot2
Rps6kb2
Gsk3b
Foxo1
Pik3r3
Cbl
Rptor
Mapk3
Akt2
Sorbs1
Eif4ebp1
Mapk1
Crk
Cblb
Insr
Eif4e
Akt3
Pdpk1
Shc3
Nras
Eif4e2
Mknk2
Mapk9
Shc2
Shc4
Ptprf
Insulin signaling pathway
Regulation of signaling by CBL
Glycogen breakdown (glycogenolysis)

E

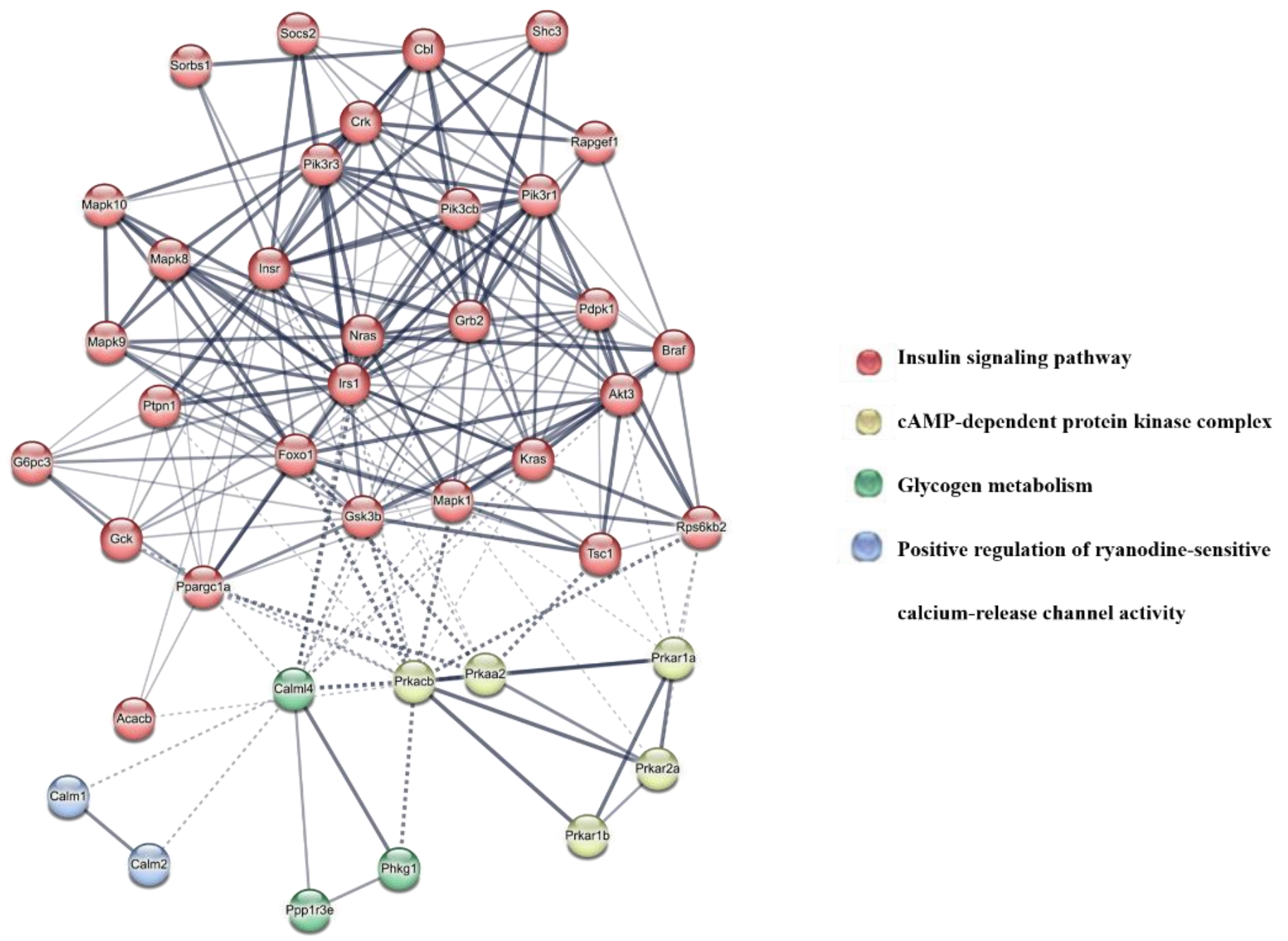

F

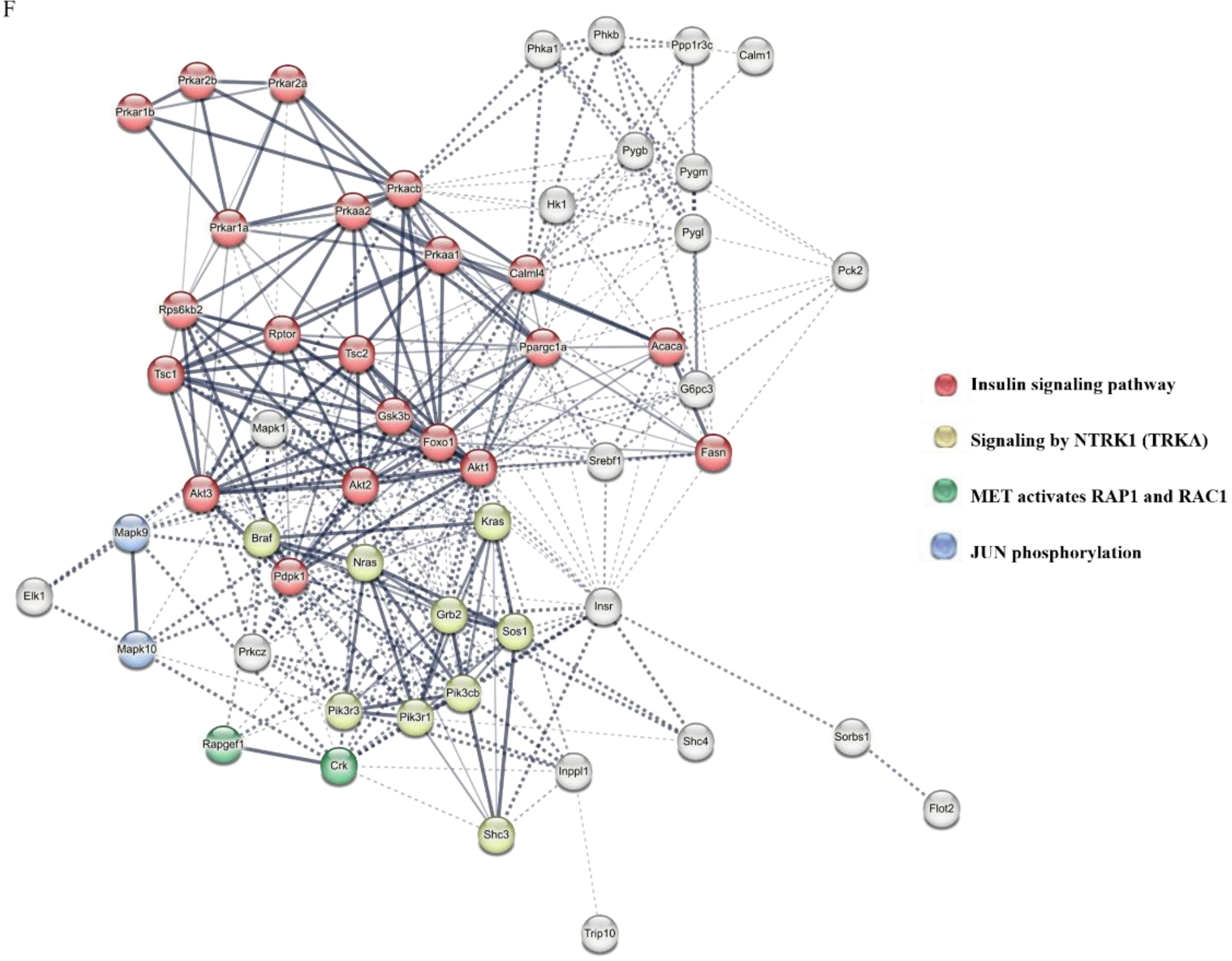

Fig. S5. Protein-protein interaction (PPI) networks of the insulin signaling pathway across core brain regions. (A-F) Targeted PPI networks constructed from DEGs mapped to the insulin cascade in the PFC (male, A; female, B), Hipp (C, D), and Amyg (E, F). Nodes represent proteins or molecular complexes, with edges denoting documented functional or physical interactions. Molecular nodes are color-coded by their association with primary metabolic pathways; specifically,

uncolored (white) nodes represent associated DEGs that interact with the core network but did not meet the statistical enrichment threshold for the highlighted functional categories.
