# Supplementary material for: Sex-dependent neurobiological and metabolic dysfunction in an aged rat model of post-traumatic stress disorder
Source: Commun Biol. 2026 Jun 19;9:1300. doi: 10.1038/s42003-026-10536-x (PMC13642903; doi:10.1038/s42003-026-10536-x)
Supplement: Supplementary file 3 [file 42003_2026_10536_MOESM5_ESM.pdf]

nature portfolio

Corresponding author(s): Peng Xiao

Last updated by author(s): Jun 4, 2026

Reporting Summary

Nature Portfolio wishes to improve the reproducibility of the work that we publish. This form provides structure for consistency and transparency in reporting. For further information on Nature Portfolio policies, see our Editorial Policies and the Editorial Policy Checklist.

Statistics

For all statistical analyses, confirm that the following items are present in the figure legend, table legend, main text, or Methods section.

n/a	Confirmed	
☐	☒	The exact sample size (*n*) for each experimental group/condition, given as a discrete number and unit of measurement
☐	☒	A statement on whether measurements were taken from distinct samples or whether the same sample was measured repeatedly
☐	☒	The statistical test(s) used AND whether they are one- or two-sided<br>*Only common tests should be described solely by name; describe more complex techniques in the Methods section.*
☒	☐	A description of all covariates tested
☐	☒	A description of any assumptions or corrections, such as tests of normality and adjustment for multiple comparisons
☐	☒	A full description of the statistical parameters including central tendency (e.g. means) or other basic estimates (e.g. regression coefficient) AND variation (e.g. standard deviation) or associated estimates of uncertainty (e.g. confidence intervals)
☐	☒	For null hypothesis testing, the test statistic (e.g. *F*, *t*, *r*) with confidence intervals, effect sizes, degrees of freedom and *P* value noted<br>*Give P values as exact values whenever suitable.*
☒	☐	For Bayesian analysis, information on the choice of priors and Markov chain Monte Carlo settings
☒	☐	For hierarchical and complex designs, identification of the appropriate level for tests and full reporting of outcomes
☐	☒	Estimates of effect sizes (e.g. Cohen's *d*, Pearson's *r*), indicating how they were calculated

Our web collection on statistics for biologists contains articles on many of the points above.

Software and code

Policy information about availability of computer code

Data collection	Behavioral data: DigiBehv professional video tracking system (Shanghai Jiliang Software Technology Co., Ltd., China).PET/CT imaging: Discoverist 180 small-animal PET/CT scanner integrated acquisition software.Transcriptomics: Illumina NovaSeq 6000 platform control software.ELISA: Microplate reader integrated software for absorbance measurement.
Data analysis	Statistical Analysis: GraphPad Prism (v9.0) and R (v4.2.1).Neuroimaging processing: Statistical Parametric Mapping (SPM) toolbox , SPM-based Anatomical Toolbox (SAMIT) , Amide, and MATLAB.PET Kinetic Modeling: Patlak analysis (calculated via MATLAB/Amide).Bioinformatics pipeline: FastQC (quality control) , HISAT2 (alignment) , Feature-Counts (quantification) , and DESeq2 (differential expression analysis).Network Analysis: STRING database for protein-protein interaction (PPI) networks , including MCL and K-means clustering algorithms.

For manuscripts utilizing custom algorithms or software that are central to the research but not yet described in published literature, software must be made available to editors and reviewers. We strongly encourage code deposition in a community repository (e.g. GitHub). See the Nature Portfolio guidelines for submitting code & software for further information.

Data

Policy information about availability of data

All manuscripts must include a data availability statement. This statement should provide the following information, where applicable:

- Accession codes, unique identifiers, or web links for publicly available datasets
- A description of any restrictions on data availability
- For clinical datasets or third party data, please ensure that the statement adheres to our policy

The RNA-sequencing datasets (raw and processed files) generated and analyzed during this study have been deposited in the Gene Expression Omnibus (GEO) repository under accession number GSE327211. Other raw data supporting the findings of this study, including behavioral and PET imaging datasets, are available from the corresponding author upon reasonable request.

Research involving human participants, their data, or biological material

Policy information about studies with human participants or human data. See also policy information about sex, gender (identity/presentation), and sexual orientation and race, ethnicity and racism.

Reporting on sex and gender	The study did not involve human participants.
Reporting on race, ethnicity, or other socially relevant groupings	The study did not involve human participants.
Population characteristics	The study did not involve human participants.
Recruitment	The study did not involve human participants.
Ethics oversight	The study did not involve human participants.

Note that full information on the approval of the study protocol must also be provided in the manuscript.

Field-specific reporting

Please select the one below that is the best fit for your research. If you are not sure, read the appropriate sections before making your selection.

☒ Life sciences ☐ Behavioural & social sciences ☐ Ecological, evolutionary & environmental sciences

For a reference copy of the document with all sections, see nature.com/documents/nr-reporting-summary-flat.pdf

Life sciences study design

All studies must disclose on these points even when the disclosure is negative.

Sample size	No statistical methods were used to pre-determine sample size. The sample size (n = 6 per group, total n = 24) was chosen based on established literature in small-animal FDG-PET and transcriptomic studies to ensure adequate statistical power for identifying regional metabolic and molecular differences while minimizing animal use.
Data exclusions	No data were excluded from the analyses.
Replication	All experimental findings were consistently observed across individuals within each group. Behavioral and PET assessments were performed in a single large cohort to ensure environmental and procedural consistency. All attempts at replication were successful.
Randomization	Rats were randomly allocated into four experimental groups (CK male, PTSD male, CK female, PTSD female) balanced by body weight within each sex to ensure a balanced distribution of baseline characteristics.
Blinding	Investigators were not blinded to group allocation during PET scanning and tissue collection. However, behavioral data were automatically recorded and quantified using the DigiBehv video tracking system to minimize observer bias. PET ROI quantification was performed using standardized, automated anatomical templates (SAMIT/SPM) to ensure objective analysis.

Reporting for specific materials, systems and methods

We require information from authors about some types of materials, experimental systems and methods used in many studies. Here, indicate whether each material, system or method listed is relevant to your study. If you are not sure if a list item applies to your research, read the appropriate section before selecting a response.

Materials & experimental systems

n/a	Involved in the study
☒	☐ Antibodies
☒	☐ Eukaryotic cell lines
☒	☐ Palaeontology and archaeology
☐	☒ Animals and other organisms
☒	☐ Clinical data
☒	☐ Dual use research of concern
☒	☐ Plants

Methods

n/a	Involved in the study
☒	☐ ChIP-seq
☒	☐ Flow cytometry
☒	☐ MRI-based neuroimaging

Animals and other research organisms

Policy information about studies involving animals; ARRIVE guidelines recommended for reporting animal research, and Sex and Gender in Research

Laboratory animals	15-month-old male and female Sprague-Dawley (SD) rats (supplied by Wuhan Luobin Life Science Co., Ltd., China).
Wild animals	The study did not involve wild animals.
Reporting on sex	Both male and female rats were included in the study design to investigate sex-dependent vulnerabilities in PTSD. Findings apply to both sexes, and sex-based analyses were performed using two-way ANOVA (Sex × Group) to identify specific interactions. Group sizes were balanced (n = 6 per sex per group, total n = 24).
Field-collected samples	The study did not involve samples collected from the field.
Ethics oversight	All experimental protocols adhered to the guidelines of the National Animal Ethics Committee and received formal approval from the Ethics Committee of Huazhong University of Science and Technology.

Note that full information on the approval of the study protocol must also be provided in the manuscript.

Plants

Seed stocks	The study did not involve plant material.
Novel plant genotypes	The study did not involve plant material.
Authentication	The study did not involve plant material.
