# Supplementary material for: Sex-dependent neurobiological and metabolic dysfunction in an aged rat model of post-traumatic stress disorder
Source: Commun Biol. 2026 Jun 19;9:1300. doi: 10.1038/s42003-026-10536-x (PMC13642903; doi:10.1038/s42003-026-10536-x)
Supplement: Supplementary file 4 [file 42003_2026_10536_MOESM3_ESM.docx]

Description of Additional Supplementary Files

File name: Supplementary Data 1

### Description:

The standalone spreadsheet uploaded alongside our manuscript is formally registered as Supplementary Data 1. This consolidated file comprises multiple separate tabs explicitly organized to fully disclose the underlying raw numerical individual values for the multi-level longitudinal behavioral assessments, peripheral endocrine ELISA markers, and subregional continuous micro-PET dynamic trajectories presented within Main Figures 2, 3, 4, 5, and 6, as well as the paired individual data for the Pearson correlation analyses outlined in Supplementary Figures 4A and 4B.
